# Short-range quorum sensing controls horizontal gene transfer at micron scale in bacterial communities

**DOI:** 10.1038/s41467-021-22649-4

**Published:** 2021-04-19

**Authors:** Jordi van Gestel, Tasneem Bareia, Bar Tenennbaum, Alma Dal Co, Polina Guler, Nitzan Aframian, Shani Puyesky, Ilana Grinberg, Glen G. D’Souza, Zohar Erez, Martin Ackermann, Avigdor Eldar

**Affiliations:** 1 Department of Environmental Systems Science, ETH Zürich, Zürich, Switzerland; 2 Department of Environmental Microbiology, Swiss Federal Institute of Aquatic Science and Technology (Eawag), Dübendorf, Switzerland; 3 Department of Evolutionary Biology and Environmental Studies, University of Zürich, Zürich, Switzerland; 4 Swiss Institute of Bioinformatics, Lausanne, Switzerland; 5 The Shmunis School of Biomedicine and Cancer Research, Tel-Aviv University, Tel-Aviv, Israel; 6 School of Engineering and Applied Sciences, Harvard University, Cambridge, MA USA; 7 Department of Molecular Genetics, Weizmann Institute of Science, Rehovot, Israel; 8 Present Address: Department of Microbiology and Immunology, University of California, San Francisco, San Francisco, CA USA

**Keywords:** Bacteria, Microbial communities

## Abstract

In bacterial communities, cells often communicate by the release and detection of small diffusible molecules, a process termed quorum-sensing. Signal molecules are thought to broadly diffuse in space; however, they often regulate traits such as conjugative transfer that strictly depend on the local community composition. This raises the question how nearby cells within the community can be detected. Here, we compare the range of communication of different quorum-sensing systems. While some systems support long-range communication, we show that others support a form of highly localized communication. In these systems, signal molecules propagate no more than a few microns away from signaling cells, due to the irreversible uptake of the signal molecules from the environment. This enables cells to accurately detect micron scale changes in the community composition. Several mobile genetic elements, including conjugative elements and phages, employ short-range communication to assess the fraction of susceptible host cells in their vicinity and adaptively trigger horizontal gene transfer in response. Our results underscore the complex spatial biology of bacteria, which can communicate and interact at widely different spatial scales.

## Abstract (web summary)

Bacterial cells often communicate by the release and detection of small diffusible molecules, a process termed quorum-sensing. Here, the authors compare the communication range of quorum-sensing systems, and identify systems that enable cells to accurately detect micron-scale changes in community composition.

## Introduction

**Fig. 1:**
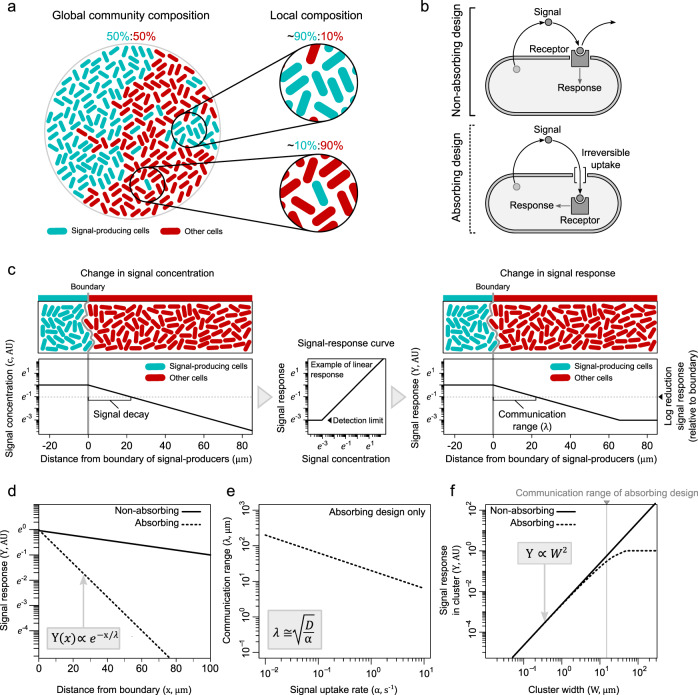
Predicted communication ranges in absorbing and nonabsorbing quorum-sensing systems. **a** Differences in community composition at local and global spatial scales. **b** Quorum-sensing designs (see also Supplementary Fig. 4). **c** Model setup with clusters of signal-producing cells (blue) embedded in a community of nonproducers (red). In the case of linearity in both signal response and uptake, the spatial decay in the signal concentration (left) corresponds to that in signal response (right). We define the communication range ($\lambda$) as the distance from the boundary of signal producers over which the signal response reduces one order of magnitude (on a natural log scale; dashed horizontal line) (see Supplementary Discussion, Section 1.3 and Supplementary Fig. 1). **d** Decline in signal response ($Y$) in absorbing (dashed line) and nonabsorbing (solid line) systems as a function of the distance ($x$, in μm) from the boundary of signal producers. **e** Communication range as a function of signal uptake rate ($\alpha$) in absorbing systems (for nonabsorbing systems $\alpha =0$). Uptake rates used here are of realistic values for RNPP systems^29,30^ and the signal diffusion rate used is 400 μm^2^ s^−1^ (Supplementary Discussion, Section 1.4.2). **f** Average signal response in clusters of signal producers with different widths ($W$). Equations in (**c**–**e**) show analytical modeling predictions for the absorbing system. For a more detailed mathematical comparison of different quorum-sensing designs see Supplementary Discussion, Section 2.

Many bacteria live in densely packed bacterial communities, where spatial growth results in highly structured and dynamic environments^1^. The genetic composition of those environments can be widely different across spatial scales: communities that are diverse at a large scale can be dominated by single species locally (Fig. 1a). Bacteria can employ quorum-sensing systems to navigate themselves in these complex spatial environments^2–5^. Signal molecules are generally thought to broadly diffuse in space, providing information about the overall composition of the community. At the same time, many quorum-sensing systems are known to regulate traits that strongly depend on the local cell composition, like conjugative transfer^6–8^, which suggests that cells may profit from limiting their communication range to nearby cells. Here, we determine how the regulatory design of quorum-sensing systems affects the range of communication, and how this range, in turn, affects the functional benefits of quorum sensing.

The communication range of quorum-sensing systems depends on both the distance over which signal molecules propagate in space and the ability of cells to respond to those molecules (see Fig. 1c). When examining how signal molecules propagate away from a cluster of signal producers, the exponential decline in signal concentration can be described by the signal decay length scale, which is the distance from over which the signal concentration reduces one order of magnitude (Fig. 1c and Supplementary Discussion, Section 1.3). When cells respond linearly to the signal concentration (Fig. 1c), the reduction in signal concentration directly translates into a similar reduction in the signal response, bound by possible detection limits of the cells. For the purpose of comparing different quorum-sensing systems, we, therefore, define the communication range as the distance from the boundary of signal producers over which the signal response reduces one order of magnitude (see Supplementary Discussion, Section 1.3 and Supplementary Figs. 1–3 for a detailed discussion on the communication range, including the impact of nonlinear signal response curves on the communication range).

In bacterial communities, signal propagation may be attenuated by signal uptake or degradation^9,10^ (Supplementary Discussion, Section 1.3). With regard to signal uptake, we can broadly categorize quorum-sensing systems into two types of design^11–13^ (Fig. 1b, Supplementary Fig. 4, and Supplementary Discussion, Section 1.1). In the first type, here called the nonabsorbing design, signaling molecules can continue to propagate after sensing. This applies to systems where the signal molecules are sensed externally by membrane-bound receptors, like in many Vibrio species^14^ and Gram-positive bacteria^15^, and also to systems where the signal is sensed intracellularly and is subsequently secreted, like in most Gram-negative bacteria using the acyl homoserine lactone-based quorum-sensing systems^16^. In the second type, the absorbing design, signal molecules are irreversibly taken up prior to sensing, preventing them from further propagating in space. In this type of design, signal sensing is coupled to signal sequestering. This regulatory design mainly includes the superfamily of peptide-based RNPP (Rap, NprR, PrgX, PlcR) systems found in Gram-positive bacteria^17^ (Supplementary Discussion, Section 1.1).

Although the communication range has been studied in several nonabsorbing systems before, both synthetic^18–25^ and natural^4,26–28^, it is unclear how these systems compare to signal-absorbing quorum-sensing systems. Here, we show that absorbing systems operate on a micron-scale due to the irreversible uptake of their signal molecules. We show that this results in a highly localized regulation of horizontal transfer in phages and integrative and conjugative elements (ICEs).

## Results

### Absorbing systems are predicted to mediate short-range communication

To compare the absorbing and nonabsorbing quorum-sensing designs, we first constructed a spatially explicit model that simulates the communication between clusters of signal-producing cells within a community of nonproducing cells (Fig. 1b, c and Supplementary Discussion, Section 2). Assuming a linear signal-response curve without detection limits, the model shows that absorbing quorum-sensing systems produce well-defined communication ranges that depend on both the diffusion and uptake rates. We draw three key experimental predictions from the model: first, signal-absorbing systems have much shorter communication ranges than nonabsorbing systems, due to the irreversible signal uptake (Fig. 1d and Supplementary Fig. 5), and assuming that signal molecules are taken up by most cells in the community, like expected for the peptide-based RNPP systems (see Supplementary Discussion, Section 1.1.3). Second, in extension of the first prediction, higher uptake rates shorten the communication range (Fig. 1e and Supplementary Fig. 5). Based on existing estimates for the rates of signal diffusion and uptake^29,30^, we predict that signal propagation is limited to a few microns only in signal-absorbing systems (Supplementary Discussion, Section 1.4.2), which starkly contrast the millimeter-range signal propagation observed in nonabsorbing systems^4,18–28^. Third, in both absorbing and nonabsorbing systems, the signal concentration provides information about the size of the signal-producing cluster (Fig. 1f). While for nonabsorbing systems, the signal concentration is effectively unbounded, strengthening the signal response with cluster size, in signal-absorbing systems the concentration saturates when the cluster radius is larger than the communication range (Supplementary Discussion, Section 2). In other words, absorbing systems provide local information only.

### Communication ranges in synthetic communities of *Bacillus subtilis* cells

**Fig. 2:**
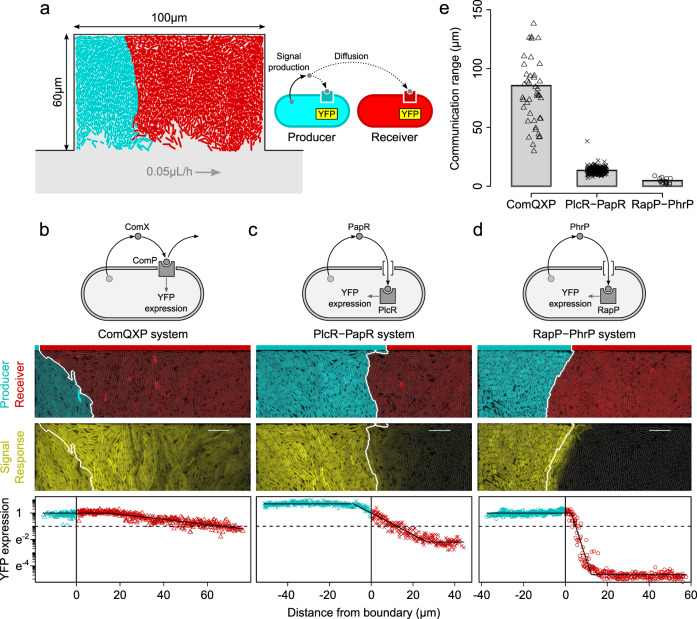
Communication range in absorbing and nonabsorbing quorum-sensing systems. **a** Representation of growth chamber with signal-producing (blue, marked by constitutive BFP) and signal-receiving cells (red, marked by constitutive RFP). Role of producers and receivers is illustrated on the right. Communication ranges in **b** ComQXP system, **c** PlcR-PapR system, and **d** RapP-PhrP system, from top to bottom: schematic depiction of quorum-sensing system (see also Supplementary Fig. 9), microscopy image with the distribution of signal producers (blue) and receivers (red) in microfluidic chamber, microscopy image of signal response (YFP expression; scale bar = 10 μm), and signal response as a function of the distance of cells to the boundary of signal producers (thick white line in microscopy images). Signal-producing cells are marked in blue with negative distances, while receivers are marked in red with positive distances. Black line marks best-fit curves (see “Methods”). Horizontal dotted line shows one order of magnitude reduction in signal response (on natural log scale). Solid vertical line in shows boundary. **e** Communication ranges of ComQXP, PlcR-PapR, and RapP-PhrP quorum-sensing systems (${\chi }_{(2)}^{2}=141.98,\;{p} < {10}^{-16}$). Source data are provided in Supplementary Data 1.

We test our modeling predictions in an experimental setup that enables us to quantify communication ranges, at a single-cell resolution, in synthetic communities of quorum-sensing bacteria. For this, we make use of the Gram-positive model species *B. subtilis*. We grow *B. subtilis* cells in microfluidic chambers of 100 μm × 60 μm × 0.83 μm (Fig. 2a and Supplementary Fig. 6, see “Methods”), where they are exposed to constant environmental conditions and form a monolayer. Chambers open on one side into a main flow channel, which provides a constant supply of nutrients and serves as a sink for waste products and signal molecules. The cell density in a fully occupied chamber reaches a volume fraction of $70 \pm 1 \%$ (mean ± s.d., $n=135$ chambers; Supplementary Fig. 7a), which is comparable to the density of cells in natural bacterial communities^31^ ($1477\pm 113$ cells/chamber, mean ± s.d., $n=362$ chambers; equivalent to a local density of $\sim 5\times {10}^{11}$ cells/ml; Supplementary Fig. 7b). Using fluorescent markers, we tracked different genotypes in the community (Fig. 2a). These genotypes typically grew as cell clusters, whose size and shape changed in time (Supplementary Fig. 8). Our microfluidic setup, therefore, includes all key attributes that shape quorum-sensing signal gradients in bacterial communities: cluster formation, signal production, accumulation, and elimination by uptake, degradation, and flow.

We start by analyzing three quorum-sensing systems (see also Supplementary Discussion, Section 1.2 and Supplementary Fig. 9): one with a nonabsorbing design, the ComQXP system^32^; and two with an absorbing design, the RapP-PhrP^33^ and PlcR-PapR systems^34^. The first two quorum-sensing systems are endogenous to *B. subtilis*, while PlcR-PapR is an exogenous system (from *Bacillus thuringiensis*) that we transformed into the same *B. subtilis* background (PY79) as the other two systems. For each system, we constructed a synthetic bacterial community of two genotypes: a signal-producing genotype (producer), which contains the entire quorum-sensing system; and a signal-receiving genotype (receiver) that only contains the genes required for signal reception (Fig. 2a, see “Methods”). Both genotypes express a yellow fluorescent protein (YFP) reporter upon sensing the signal molecule, but only the producer can secrete the signal, which makes it possible to quantify the distance over which signal molecules diffuse in space (see “Methods”). We use this experimental setup to test our three modeling predictions.

### Absorbing systems facilitate short-range communication

We start by testing the first modeling prediction that nonabsorbing systems have a longer communication range than absorbing systems. For this purpose, we focus on chambers where genotypes separate into two distinct regions: a region of producers and a region of receivers (Fig. 2b–d, see “Methods”). In the region of producers, all systems show a saturated signal response, indicating high signal concentrations (YFP expression; Fig. 2b–d and Supplementary Fig. 10). In the region of receivers, the signal response gradually declines with distance. For the PlcR-PapR system, this decline is evident from the boundary (i.e., vertical solid line in Fig. 2b–d) between signal producers and receivers, for the other two systems, the decline occurs after a certain range over which cells express a saturated signal response. Since we define the communication range as the spatial distance over which the signal response (YFP expression) reduces one order of magnitude (on a natural log scale; horizontal dashed line in Fig. 2b–d), the communication ranges of the ComQXP and RapP-PhrP systems are composed of two parts, the saturated signal response and the response decay (see Supplementary Figs. 1 and 10 for details).

In agreement with previous studies^4,26–28^, we find that the nonabsorbing ComQXP system supports long-range communication ($87.0\pm 6.1\,{\upmu}{\mathrm{m}}$, mean±s.e., $n=42$ chambers from ten flow channels; see Supplementary Movie 1), consistent with a free-diffusion model with no significant uptake or degradation of signal molecules (Supplementary Discussion, Section 2). In contrast, the absorbing systems give rise to highly localized communication (Fig. 2): $13.4\pm 0.2\,{\upmu}{\mathrm{m}}$ for the PlcR-PapR system ($n=218$ chambers from $14$ flow channels; see Supplementary Movie 2) and $4.7\pm 0.6\,{\upmu}{\mathrm{m}}$ for RapP-PhrP ($n=16$ chambers from three flow channels; see Supplementary Movie 3). To confirm that these short communication ranges result from limited signal propagation, as predicted by the model, and not from the way cells respond to different signal concentrations, we measured the signal-response curves of both absorbing systems by exposing cells to different concentrations of synthetic signal molecules (Supplementary Fig. 11). Using the slopes of these curves (i.e., Hill coefficient; Supplementary Discussion, Section 1.3 and Supplementary Fig. 11), we can derive the signal decay length scales (see also Supplementary Discussion, Section 1.4.3). Indeed, as expected with irreversible signal uptake, both absorbing quorum-sensing systems show strongly limited signal propagation: $12.6\pm 0.2\,{\upmu}{\mathrm{m}}$ for the PlcR-PapR system (Hill coefficient $\approx 0.92$; $n=218$ chambers from $14$ flow channels) and $6.8\pm 0.8\,{\upmu}{\mathrm{m}}$ for RapP-PhrP (Hill coefficient $\approx 2.26$; $n=16$ chambers from three flow channels). We next examine the role of signal uptake in more detail.

### Signal uptake determines the range of communication in an absorbing system

**Fig. 3:**
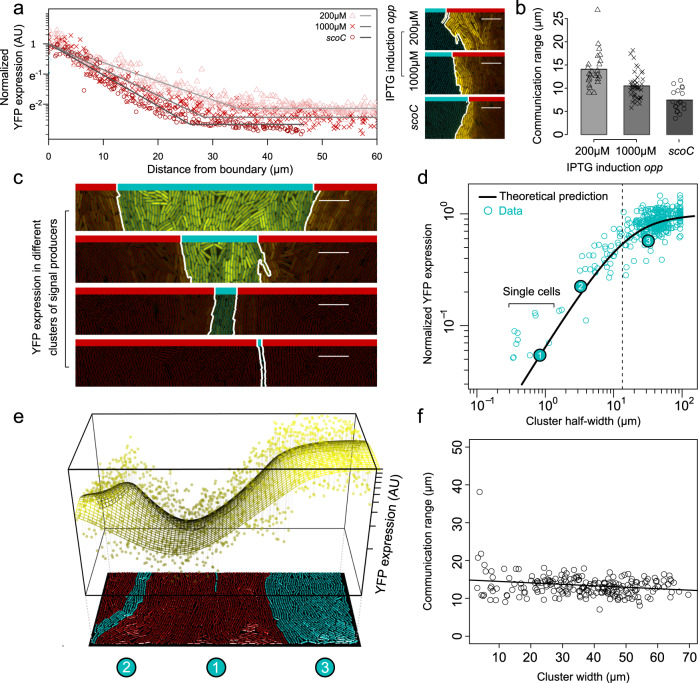
Signal uptake leads to a shorter communication range in absorbing PlcR-PapR system. **a** Normalized YFP expression in signal receivers as a function of their distance to the producers in IPTG-inducible *opp* system and *scoC* knockout mutant (right, representative microscopy images). Signal producers in (**a**) do not express YFP. Gray lines show best fits (see “Methods”). **b** Communication range decreases for higher IPTG concentrations in the IPTG-inducible *opp* system ($U=825,\;{p}=2.7\times {10}^{-5}$) and decreases even further in the *scoC* knockout ($U=648,\;{p}=1.5\times {10}^{-4}$) (200 μM IPTG, $n=24$; 1000 μM IPTG, ${n}=43$; *scoC*, $n=19$; statistics show two-sided Mann–Whitney *U* tests without adjustments for multiple comparisons). **c** Representative microscopy images of differently sized clusters of signal producers. **d** Normalized YFP expression in isolated clusters of signal producers as a function of their half-width (blue dots), observed YFP expression ($n=362$ chambers). YFP levels are normalized to their maximal levels; black line, theoretical prediction based on the measured communication range (see “Methods”); vertical dashed line, the communication range of PlcR-PapR system. Enlarged data points 1–3 correspond to three co-occurring clusters of signal producers visualized in (**e**). **e** Observed (dots) and predicted (grid surface) YFP expression in chamber with three co-occurring clusters of signal-producing cells. **f** Communication range as a function of the cluster size for clusters wider than 3 μm (linear regression: intersect = $14.8\pm 0.5\,{\upmu} {\mathrm{m}}\;({\mathrm{s.e.}})$, slope = $-0.04\pm 0.01\,{\upmu} {\mathrm{m}}$, $p=0.0014,\;{n}=213$ chambers). Microscopy images: blue bars = region of signal producers; red bars = region of signal receivers; yellow = signal response; scale bar = 10 μM. Source data are provided in Supplementary Data 1.

The second model prediction is that higher signal uptake rates would shorten the communication range. We test this prediction in the exogeneous PlcR-PapR system. This system shows an approximately linear-response curve for a wide range of signal concentrations without saturation, which enables us to accurately determine the communication range for a large range of signal uptake rates (in contrast, the endogenous RapP-PhrP system shows a nonlinear signal response with strong saturation; Fig. 2c, d and Supplementary Fig. 11). We modulate the signal uptake rate by inducing the expression of the oligopeptide permease (Opp) system, using a strain where the native *opp* promoter is replaced by an isopropyl-β-d-thiogalactopyranoside (IPTG)-inducible promoter^35^. In this way, cells only express the Opp system in the presence of IPTG, and we can fully control the rate of signal uptake (Supplementary Fig. 12): without IPTG, there is no signal uptake, and with high IPTG concentrations, the uptake rate is high. Besides this inducible strain, we also examine a knockout strain of the major transcriptional repressor (*scoC*) of the *opp* operon. This knockout strain strongly overexpresses the Opp system^36^ (Supplementary Fig. 12), without affecting the slope of the signal-response curve (Supplementary Fig. 11). As expected, increasing the signal uptake rate, by inducing Opp expression, significantly shortens the communication range ($200\,{\upmu}{\mathrm{M}}$ IPTG: $14.1\pm 0.8\,{\upmu}{\mathrm{m}}$, $n=24$ chambers from two flow channels; and $1000\,{\upmu}{\mathrm{M}}$ IPTG: $10.5\pm 0.4\,{\upmu}{\mathrm{m}}$, $n=43$ chambers from four flow channels; Fig. 3a, b). The communication range is shortest in the *scoC* knockout mutant: $7.4\pm 0.5\,{\upmu}{\mathrm{m}}$ ($n=19$ chambers from three flow channels). Conversely, by coculturing the IPTG-inducible *opp* system with a strong signal producer, we can also measure the signal response for much lower signal uptake rates than those observed in the wild type ($40\,{\upmu}{\mathrm{M}}$ IPTG; Supplementary Fig. 12). As expected, lowering the uptake rate substantially increases the communication range ($22.5\pm 1.4\,{\upmu}{\mathrm{m}}$, $n=11$ chambers from three flow channels; Supplementary Fig. 13).

### Short-range communication provides local neighborhood information

The third and final model prediction is that signal molecules inform the cells about the size of the signal-producing cluster: higher concentrations indicate larger clusters. In the case of short-range communication, this information is bounded (Fig. 1f): when clusters have a radius equal to or larger than the communication range, the signal concentration is predicted to saturate. To test this prediction, we examine the signal response in the PlcR-PapR system for clusters of varying sizes (Fig. 3c). Figure 3d shows the predicted and observed signal response in clusters ranging from $\sim 0.5$ (single cell) to 100 μm (full chamber) in radius (see also “Methods, ‘Cluster size and signal response analysis’”). In close agreement with our theoretical predictions (Supplementary Discussion, Section 2.1.3), the signal response saturates in clusters with a radius larger than the communication range (13.4 μm in this case). In other words, cells do not sense the entire cluster, but rather the density of signal-producing cells within their local neighborhood. Signal-producing cells outside this communication range are not detected. The lack of crosstalk is illustrated in Fig. 3e, where three co-occurring clusters of signal producers show YFP expression levels that are equal to that of clusters in isolation (Fig. 3d).

Finally, we tested how the cluster size affects the communication range. In contrast to the signal uptake rate, the cluster size and hence the total signal production rate is predicted to have no effect on the communication range (Supplementary Discussion, Section 2). This was found to be the case for the vast majority of clusters, which show communication ranges of ~15 μm (Fig. 3f). Only for very small clusters (<3 μm), consisting of a few cells, we found deviating communication ranges that could be attributed to a nonlinear signal response at low signal concentrations^37^ (see Supplementary Discussion, Sections 1.3.2 and 1.4.5 and Supplementary Figs. 14, 15, 16, and 22 for details). The fact that—apart from these small clusters—the communication range is constant with cluster size (Fig. 3f) entails that the signal decay does not change with cluster size (Fig. 3d). Given that larger clusters produce more signal than small clusters, while the signal decay is constant, one expects that for large clusters the signal concentration would be above the detection limit for a wider spatial range (see Supplementary Discussion, Section 1.3 and Supplementary Fig. 17 for details). This is indeed what we observe (Supplementary Fig. 17).

### Short-range communication adaptively controls horizontal gene transfer

**Fig. 4:**
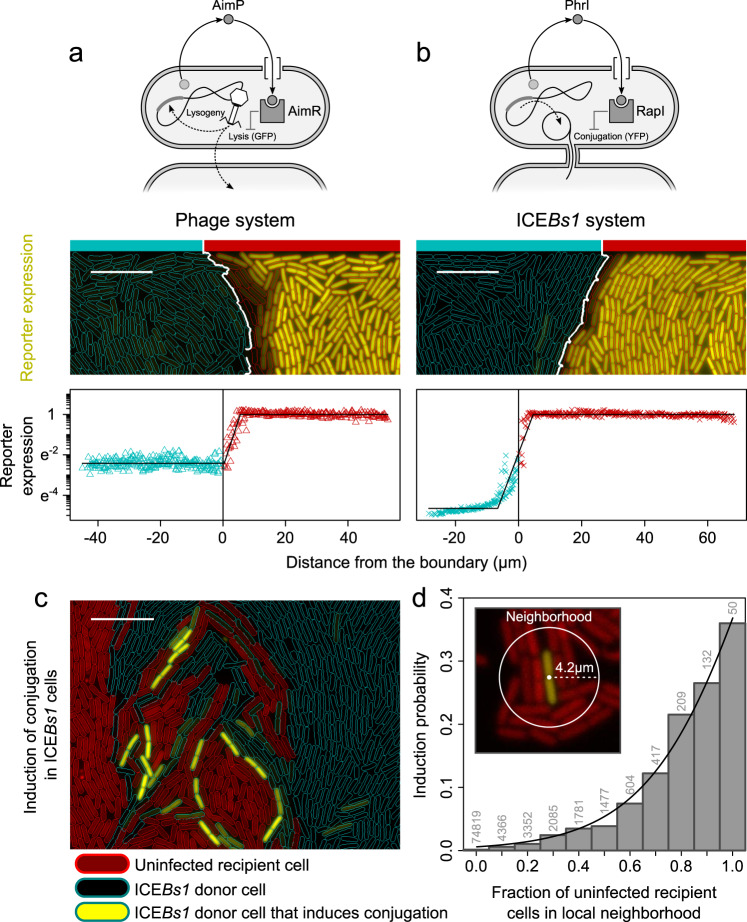
Short-range communication regulates horizontal gene transfer. Quorum-sensing systems of **a** phage system (φ3T) and **b** integrative conjugative element (ICE*Bs1*). From top to bottom: scheme of quorum-sensing system, distribution of signal producers (blue outlines) and receivers (red outlines) and their reporter expression, GFP expression for φ3T system (artificially colored yellow), and YFP expression for *ICEBs1* system (scale bar = 10 μm), and reporter expression as a function of the distance of cells to the boundary of signal producers. Reporter gene expression indicates induction of horizontal gene transfer: lytic lifestyle for phage system and conjugation for ICE*Bs1* element. Black line marks best-fit curves (see “Methods”). The zone of inhibition is determined by the spatial distance from the boundary of signal producers, where we observe repression in reporter gene expression (Supplementary Fig. 19). **c** Representative microscopy image of coculture of Δ*conB* ICE*Bs1* donor cells (blue outline) and uninfected recipient cells (red outline) (scale bar = 10  μm). Donor cells that express YFP induce conjugation. **d** Induction probability as a function of fraction of uninfected recipient cells in the local neighborhood ($n={\mathrm{89,292}}$ cells; numbers above bars in plot show how cells are distributed across neighborhoods). Neighborhood size is determined by the zone of inhibition. Induction probability increases significantly with fraction of uninfected recipient cells in the local neighborhood (regression: $y=\frac{1}{1+{e}^{a\left(x+b\right)}}{;\; a}=-4.7\pm 0.2\;\left({\rm{s}}.{\rm{e}}.\right),\;{p}_{a} < {10}^{-9}{;\;b}=-1.1\pm 0.01,\;{p}_{b} < {10}^{-14}$; ${\rm{residual}}\; {\rm{s}}.{\rm{e}}.=0.01;\;{\rm{d.f.}}=9$). Source data are provided in Supplementary Data 1.

Since short-range communication provides information about the local community composition, we expect that cells could adaptively employ absorbing quorum-sensing systems to control phenotypic traits whose benefits depend on nearby cells. One key example of such phenotypes is horizontal gene transfer. Mobile genetic elements often require physical proximity between the donor and susceptible cells to effectively spread through a community. Plasmids and integrative conjugative elements (ICEs), for instance, rely on physical contact between cells for conjugation. Similarly, the restricted diffusion of phages inside biofilms limits their effective range of infection to nearby cells^38^. To regulate horizontal gene transfer, many mobile genetic elements make use of quorum-sensing systems of the absorbing design. This includes plasmids^6,7^, ICEs^8^, and temperate phages^39,40^ (Fig. 4a, b and Supplementary Fig. 18). In all these systems, signal molecules repress the infective form of the mobile genetic element. This was proposed to reduce infectivity when the majority of cells are already infected^8,39^. We hypothesize that short-range communication allows these mobile elements to specifically assess the pool of susceptible host cells in their immediate vicinity and trigger horizontal gene transfer when there are many uninfected cells. To test this hypothesis, we examine the arbitrium (AimR-AimP) quorum-sensing system of the phage φ3T (Fig. 4a), which regulates the lysis–lysogeny decision upon infection^39^ and the RapI-PhrI quorum-sensing system of the ICE*Bs1* element (Fig. 4b), which regulates conjugation^8^ (Supplementary Discussion, Section 1.2).

We start our analysis by quantifying the communication range of each quorum-sensing system using the same experimental setup as above. In the phage system, producers contain the genes necessary for signal production and reception, while receivers only contain genes necessary for signal reception. In the ICE*Bs1* system, producers contain the entire ICE*Bs1* element, with a *conB* knockout mutation to prevent actual transfer^41^, while receivers only contain the genes necessary for signal reception (see “Methods” for details and Supplementary Fig. 18). We use fluorescent reporters to monitor the induction of horizontal gene transfer: the lytic cycle in the phage φ3T system (Fig. 4a) and conjugation in the ICE*Bs1* system (Fig. 4b).

We found remarkably short communication ranges: $1.61\pm 0.08\,{\upmu} {\mathrm{m}}$ (mean ± s.e., $n=45$ chambers from two flow channels) for the arbitrium quorum-sensing system of phage φ3T and $1.98\pm 0.10\,{\upmu} {\mathrm{m}}$ ($n=14$ chambers from two flow channels) for the RapI-PhrI quorum-sensing system of the ICE*Bs1* element (Supplementary Fig. 19; note that here the signal response is repression of reporter gene expression, thus the communication range is determined by a log-fold increase in reporter gene expression). Besides the communication range, we also determined the zone of inhibition, which is the spatial distance from the boundary of signal producers where we observe repression in reporter gene expression: $6.6\pm 0.2\,{\upmu} {\mathrm{m}}$ (mean ± s.e., $n=45$ chambers from two flow channels) for the arbitrium quorum-sensing system of phage φ3T and $4.2\pm 0.2\,{\upmu} {\mathrm{m}}$ ($n=14$ chambers from two flow channels) for the RapI-PhrI quorum-sensing system of the ICE*Bs1* element (Fig. 4a, b; see “Methods,” Supplementary Fig. 19, and Supplementary Movies 4 and 5). Interestingly, even though all signal producers have a reporter, they rarely express it. In fact, in the phage system, the lytic cycle was always shut down, even when producers were surrounded by receivers only (Supplementary Fig. 20). This suggests that, in our experimental setup, single producer cells already accumulate sufficient signal to prevent horizontal gene transfer. In contrast, in the ICE*Bs1* system, we do observe frequent induction of horizontal gene transfer in producer cells, with an average induction probability of 0.6% ($n=89,292$ cells).

We hypothesized that the ICE*Bs1* element adaptively triggers conjugation in response to the fraction of susceptible recipient cells within their local neighborhood. To test this, we grew donor cells, carrying the ICE*Bs1* element (with *conB* mutation), together with uninfected recipient cells, lacking the ICE*Bs1* element (Fig. 4c). We then monitored which donor cells induce conjugation, based on the YFP reporter, and determined how the induction probability depends on the fraction of uninfected recipient cells in their local neighborhood. As for the neighborhood size, we use the zone of inhibition as shown in Fig. 4b and quantified in Supplementary Fig. 19b. We found that the induction probability varied more than 200-fold between neighborhoods without any recipient cells to those with only recipient cells (Fig. 4d and see Supplementary Movies 6 and 7). To verify that these results strictly depend on the RapI-PhrI quorum-sensing system, we replaced the full ICE*Bs1* system in donor cells with a minimal set of genes necessary for signaling (see “Methods”). This gave similar results (Supplementary Fig. 21 and see Supplementary Movies 6 and 7). Thus, our results show that short-range communication of absorbing quorum-sensing systems can be beneficial for regulating contact-dependent phenotypes inside bacterial communities.

## Discussion

We show that cells can communicate at dramatically different spatial scales in bacterial communities. Besides the long-range communication observed before^4,26–28,42^, we uncover a new mode of communication that acts at remarkably short spatial ranges of no more than a few microns. Short-range communication relies on a key design feature of absorbing quorum-sensing systems, where signal molecules are irreversibly taken up by cells. Although we focused on single-species communities, oligopeptide uptake systems are widespread in prokaryotes^43,44^ and are present in most bacteria, including many Gram-negative bacteria that do not employ peptide-based quorum-sensing signaling. We, therefore, expect that our results would apply as well to most multispecies communities found in nature (Supplementary Discussion, Section 1.6).

Several previous studies have quantified the communication range by measuring the maximum distance between individual cells or groups of cells over which communication could be established in the absence of intermittent cells (the so-called calling distance)^4,45^. Our study complements this approach by focusing on the range of communication within cell communities. Since the communication range in absorbing quorum-sensing systems is limited by signal uptake, short-range communication within communities does not exclude long-range communication between communities (see also Supplementary Discussion, Section 1.1), suggesting that quorum-sensing systems may act at different spatial scales depending on the spatial arrangement of cells. Our study also differs from previous studies in that we emulate a community with a constant cell density. In this environment, signal molecules do not inform the cell about the cell density, but about the community composition^30,46^, enabling cells to assess the fraction of signal-producing cells within their neighborhood as set by the communication range.

Intriguingly, in parallel to our findings, signal uptake was recently shown to limit communication during T cell maturation in mice^47^. The maturation of memory T cells depends on quorum-sensing signaling, through the release and detection of cytokines^48^. Receptor-mediated uptake of cytokines by cells within lymph nodes may therefore limit the range of cytokine-mediated quorum-sensing. In combination with our findings, these results suggest that signal uptake forms a general mechanism to limit the range of quorum-sensing across biological systems.

In addition to irreversible signal uptake, we expect that other mechanisms, such as enzymatic degradation of signal molecules, may further limit the range of communication (Supplementary Discussion, Section 1.5). More generally, our observation of the role of signal degradation and uptake in quorum-sensing systems falls in line with a long list of other critical functions of signal degradation. For example, in slime molds and yeasts, localized degradation of chemical attractants was shown to create strong signal gradients that facilitate directed motility or growth^49–51^. By the same token, during multicellular development, signal degradation affects both the width and shape of morphogen gradients^52^.

We posit that the relevant spatial scale of communication is ultimately determined by the phenotypic traits that are regulated by the quorum-sensing signals. In the case of horizontal gene transfer, local information about neighboring cells provides a better predictor than global information for whether conjugation will be successful. Similarly, short-range communication could be relevant when nearby cells strongly affect the environmental conditions to which cells are exposed in the community, due to, for example, resource consumption or production of waste products^9,53^. The spatial scale at which cells communicate may, therefore, reflect the scale at which cells interact. As such, we expect that highly localized communication, as revealed in this work, is not only important for horizontal gene transfer but could have functional implications for many bacterial phenotypes and thereby strongly impact the spatiotemporal organization of bacterial communities.

## Methods

### Growth conditions and strains

Routine *B. subtilis* growth was performed in Lysogeny Broth (LB): 1% tryptone (Difco), 0.5% yeast extract (Difco), and 0.5% NaCl. Sporulation-promoting media DSM and MSgg for ICE*Bs1* transconjugation were prepared as described before^33^. When preparing plates, the medium was solidified by the addition of 2% agar. Antibiotics were added (when necessary) at the following concentrations: spectinomycin: 100 µg ml^−1^, tetracycline: 10 µg ml^−1^, chloramphenicol: 5 µg ml^−^^1^, kanamycin: 10 µg ml^−^^1^, MLS: 3 µg ml^−^^1^ erythromycin + 25 µg ml^−1^ lincomycin, ampicillin for *Escherichia coli*: 100 µg ml^−1^. Experiments were performed using Spizizen minimal medium^54,55^ (final concentration: 2 g l^−1^ (NH_4_)_2_SO_4_, 14 g l^−1^ K_2_HPO_4_, 6 g l^−1^ KH_2_PO_4_, 1 g l^−1^ trisodium citrate, and 0.2 g l^−1^ MgSO_4_·7H_2_O), supplemented with 0.5% glucose and trace elements solution^55^ (final concentration: 125 mg l^−1^ MgCl_2_·6H_2_O, 5.5 mg l^−1^ CaCl_2_, 13.5 mg l^−1^ FeCl_2_·6H_2_O, 1 mg l^−1^ MnCl_2_·4H_2_O, 1.7 mg l^−1^ ZnCl_2_, 0.43 mg l^−1^ CuCl_2_·4H_2_O, 0.6 mg l^−1^ CoCl_2_·6H_2_O, and 0.6 mg l^−1^ Na_2_MoO_4_·2H_2_O). For microfluidic experiments, 0.01% Tween-20 (Polysorbate-20, Sigma-Aldrich) was added to the medium to prevent cells from sticking to the microfluidic device or tubing. In all cases, cells were grown at 37° C.

All quorum-sensing systems were cloned into the same genetic background of *B. subtilis* PY79. For details, see the next section and Supplementary Table 1. To ensure homogeneous and constitutive expression of the quorum-sensing system across the community, genes were expressed under either constitutive or IPTG (Sigma-Aldrich) inducible promoters (with exception of the phage system, whose native promoters support constitutive expression). Specifically, for the ComQXP, PlcR-PapR, RapP-PhrP systems, signal production was IPTG-inducible, using IPTG concentrations that lead to wild-type expression levels: 100 μM IPTG for the ComQXP system, 1 mM for both the PlcR-PapR and RapP-PhrP systems. For Fig. 3, where we examined the PlcR-PapR system with IPTG-inducible *opp* expression, signal production was expressed under a constitutive promoter. Finally, for the ICE*Bs1* system, where the native *rapI-phrI* operon was only weakly expressed under our growth conditions, we introduced an extra copy of the entire *rapI-phrI* operon with IPTG-inducible expression. We used slightly different induction levels for the ∆*conB* ICE*Bs1* (20 μM) and regulatory-only ICE*Bs1* systems (10 μM) to yield similar conjugation probabilities.

### Strain construction

All *B. subtilis* strains were constructed in PY79 background, unless otherwise indicated (for strain list, see Supplementary Table 1). Either standard transformation or SPP1 transduction methods were used for genomic integration and plasmid transformation^55^, and transformants were selected on plates with an appropriate antibiotic. Later bacterial DNA was purified and insertion of each construct into ectopic site in *B. subtilis* genome was verified by PCR using primers annealed outside the homologous regions (for primer list, see Supplementary Table 3). Moreover, we used a long flanking homology PCR method^56^ to delete either *scoC* or *appA* from PY79 chromosome. Both genes were replaced by a tetracycline resistance cassette. Primers used for each deletion are indicated in the primer list (Supplementary Table 3).

To replace the native promoter of the Opp operon (*spo0K*) with an IPTG-inducible one, DNA of strain AES6018 was purified, and *appA*::Tet was inserted into strain AES5829 by transformation. *appA* deletion was verified by PCR and positive colonies were grown in a sporulation-promoting medium (DSM) with and without IPTG. Colonies that have also integrated the P_spac_-inducible promoter through genomic linkage showed defective sporulation without IPTG, but sporulated well with IPTG.

To generate a PY79 donor, harboring the conjugative element ICE*Bs1* (AES6181), we cultured a donor strain (AES1574, ICEBs1-containing strain 3610, plasmid free), and a potential recipient PY79-derived strain (AES5919, ICEBs1-free strain) in minimal medium (SMM) until an OD of ~0.1. A minority (1%) of the donor strain was mixed with a majority (99%) of the recipient strain, then 5 µl was plated on MSgg plate. The plate was incubated for 72 h at 30 °C. Then, we re-suspended the colony in 5 ml sterile distilled deionized water (sDDW) and plated it on LB plates. Transconjugant colonies with a high blue fluorescent protein (BFP) and decreased YFP expression levels were selected after flow cytometry measurements.

### Plasmid construction

Standard protocols were used to construct new plasmids (for complete list of plasmids, see Supplementary Table 2). All constructed plasmids are either in DH12 or DH5α *E. coli* strains. T4 DNA ligase, T4 polynucleotide kinase, Phusion High-Fidelity DNA Polymerase, and all restriction enzymes were purchased from New England BioLabs.

In order to prepare pAEC1541, a DNA fragment containing P_*srfA*_-3xYFP was amplified from AES1334 using PTB361 and PTB453 primer pair (for primers, see Supplementary Table 3). *Kpn*I restriction enzyme site was added to pAEC1504 by PCR using back-to-back primers, PTB449 and PTB450. Both PCR products were digested by *Kpn*I-HF, *Eco*RI-HF and *Dpn*I, followed by ligation. To construct pAEC1526, the same digested vector was ligated with the insert, P_hyperspank_-*papR-lacI*, which was amplified from pAEC2148 using PTB330 and PTB373 primer pairs and digested with the same enzymes.

The *srfA* promoter in pAEC1003 was replaced by *plcA* promoter to construct pAEC1413. pAEC1003 was digested with *Nhe*I-HF and *EcoR*I-HF restriction enzymes to remove P_*srfA*_, while *plcA* promoter was amplified from pAEC1149 using PTB366 and PTB367 primer pairs. Then, it was digested by the same enzymes and both DNA fragments were ligated.

To generate pAEC1652, open-reading frames (ORFs) of *papR* were amplified from pAEC2148 using the primers PTB303 and PTB469, and digested by *Sph*I-HF, *Spe*I-HF, and *Dpn*I. To prepare the vector, pDL30 including P_*comQXP*_ was amplified from pAEC962 using PTB320 and PTB209 primer pairs. Later, the PCR product was digested by *Nhe*I-HF, *Sph*I-HF, and *Dpn*I enzymes, and both digested DNA fragments were ligated to generate the plasmid pDL30::P_*comQXP*_*-papR-*Spec.

Constructing pAEC1839 was done by amplifying *xis* promoter using PTB526 and PTB527 primer pairs. The PCR product was digested with *Bam*HI-HF and *Mfe*I-HF, and cloned into pAEC277 digested with *Eco*RI-HF and *Bam*HI-HF.

For pAEC1727 and pAEC1729 construction, ORFs of *rapI* only or *rapI*-*phrI* were amplified from *B. subtilis* NCIB3610 strain using PTB474 and either PTB475 or PTB476 primers. Both amplified DNA fragments were digested with *Sac*I-HF and *Nhe*I-HF and then were cloned into ECE174 contains P_hyperspank_-*lacI-*Cm, which was also digested by the same enzymes.

To generate pAEC1754, a DNA fragment containing the ORFs of *immR*, *immA*, and *xis* was amplified from *B. subtilis* NCIB3610 strain using PTB503 and PTB504 primer pairs. The PCR product was digested with *Bam*HI-HF and *Mfe*I-HF, and cloned into the plasmid pAEC277 digested with *EcoR*I-HF and *Bam*HI-HF. The final construct has the insert *immA-immR-xis*, followed by three YFP coding genes.

To generate the plasmid pAEC1932, which contains a coding gene for the PapR, the entire plasmid pAEC1526 without the *lacI* was amplified using the primers PTB303 and PTB449. The PCR product was treated with *Dpn*I restriction enzyme and then by T4 polynucleotide kinase, followed by self-ligation.

### Real-time quantitative PCR (qPCR)

Total RNA was extracted from *B. subtilis* PY79 cells using a High Pure RNA Isolation Kit (Roche). Cells growing in SMM with glucose and trace elements media and IPTG (if indicated) were centrifuged at OD_600_ ~0.2–0.3. One microgram of RNA was reverse transcribed to complementary DNA (cDNA) using qScript™ cDNA Synthesis Kit (Quanta BioSciences). Real-time qPCR was performed on a Step One Plus Real-Time PCR System (Applied Biosystems) using SYBR Green (Quanta BioSciences). The transcription level of *oppA*, our gene of interest, was normalized to the reference gene level: *rpoB*. Results were analyzed in the Step One™ V2.3 software. The primers used for the analysis were as follows: for *rpoB*, TCGTTACCTTGGCATTCACA and CACGGTTATCAAACGGCTCT, and for *oppA*, CGAGCAAGGACGGAAAGACA and GTCAAGCGCCCATTTCCAAG. Each strain was measured across at least three biological repeats.

### Flow cytometry

Flow cytometry was performed to quantify gene expression in Supplementary Figs. 11 and 15 at the single-cell level. We used a Beckman-Coulter Gallios flow cytometer equipped with four lasers (405 nm, 488 nm colinear with 561 nm, 638 nm). The emission filters used were BFP 450/50 nm, YFP 525/40 nm, and mCherry 620/30 nm. Strains were grown in SMM as described before^37^, and YFP levels were measured as a ratio between the measured strain and the autofluorescence of a PY79 in SMM medium. To distinguish between cocultured cells in Supplementary Fig. 15, constitutive mCherry and mTag2-BFP integrated into each strain. Supplementary Fig. 22 shows the gating procedure used for generating Supplementary Fig. 15.

For Supplementary Fig. 11, a synthetic PhrP 6-mer peptide (ADRAAT) was purchased from GL Biochem (Shanghai, China) at >98% purity, and a synthetic PapR 7-mer peptide (ADLPFEF) was purchased from BLAVATNIK CENTER for Drug Discovery (Tel-Aviv, Israel) at a purity of >95%. Lyophilized peptides were re-suspended with sterile distilled deionized water (sDDW) to prepare 10 mM aliquots. Both strains PapR and PhrP receivers were grown in minimal medium (SMM), until they reach an OD of ~0.07. Then, different concentrations of the appropriate signal peptide were added, and 3 h later, YFP expression levels were measured using flow cytometry.

### Microfluidic experiments

#### Design

All experiments were conducted with microfluidic devices that carry an identical design (Supplementary Fig. 6). There are eight parallel flow channels (200 μm wide, 22 μm deep). These main channels split into two smaller channels (100 μm wide, 22 μm deep) with 29 equally spaced chambers (100 μm wide, 60 μm deep, 0.83 μm height) on their lateral sides. Each chamber fits ~1500 cells and its narrow height ensures that cells grow in a monolayer without being pressed (Supplementary Figs. 7 and 8). The main flow channels have a single inlet and outlet, through which medium is pumped at a constant rate (see below for details; from inlet to outlet flow channels are ~2.4 cm long). For each microscopy experiment, microfluidic devices were freshly produced a day in advance (see below), using a reusable SU-8 mold that was made by a former lab member (Daan Kievit, ETH Zürich). This mold was constructed using a two-step photolithography process using SU-8 photoresist on a silicon wafer.

#### Production

Microfluidic devices were constructed by pouring polydimethylsioxane (Sylgard™ 184 Silicone Elastomer Kit, Dowsil) over the dust-free SU-8 mold, using a 1:10 mixture of the base and curing agent, following the supplier’s guidelines. Then, to remove bubbles, the freshly poured microfluidic device was placed in a desiccator for 1 h, after which it was baked at 80 °C for another hour (for curing). The microfluidic device was carefully removed from the mold using a scalpel and holes at the channel inlets and outlets were punched by hand, using a biopsy puncher (0.75 mm; WPI, No. 504529). The surface of microfluidic device was cleaned by repeatedly applying and removing the Scotch tape, which takes away most dust particles. A round cover glass (diameter = 50 mm; Menzel-Gläser, Braunschweig, Germany) was bound to the cleaned surface after treating both the cover glass and microfluidic device with oxygen plasma for 30 s at full power (PDC-32G-2 Plasma Cleaner, Harrick Plasma, New York, USA). Finally, the microfluidic device was heated on a plate at 100 °C for 1 min to remove moisture that was released during the binding process.

#### Loading

Each genotype of the synthetic community of signal producers and receivers was separately grown overnight in 4 ml SMM medium (supplemented with 0.01% Tween) at 37 °C (220 r.p.m.). The next day, the stationary phase cultures were mixed 50:50 and the resulting cell suspension was concentrated 5-fold by centrifugation (5000 × *g*, 5 min). Before loading the microfluidic device, the concentrated cell suspension was firmly re-suspended and vortexed to assure that cell aggregates were broken apart and genotypes were properly mixed. Cells were loaded into the chambers by pipetting 8 μl of concentrated cell suspension through the outlet of one of the main channels. The inlet was purposely avoided to minimize the chance of biofilm formation at the inlet. The loaded chip was subsequently placed on the pre-heated microscopy stage (37 °C) for 15–30 min, which allows cells to swim into the chambers. When the chambers were sufficiently full (>20 cells/chamber), the medium was pumped through the flow channels at a constant rate of 0.1 ml/h, thereby preventing cell growth in the main channel, while assuring laminar flow. To this end, we used syringe pumps (NE-1800, NewEra Pump Systems, New York, USA) on which we placed 50 ml syringes (Sanitex syringes, Huberlab No. 3.7470.14) with 20 ml of freshly made SMM medium. Syringes were equipped with a 0.2 μm filter (Filtropur S; Sarstedt VWR, No. 83.1826.001) to trap microscopic air bubbles in the medium, and a 20 G needle (70 mm long, OD 0.9 mm; Nipro Needle, No. HN-2070-ET). Two pieces of tubing were used to connect the syringe to the microfluidic device: (i) a thick piece of Saint-Gobain Tygon non-DEHP microbore tubing (ID 0.76 mm, OD 2.29 mm; Thermo Scientific. No. 15187044) was attached to the needle of the syringe pump and (ii) a thin and long piece of polytetrafluoroethylene tubing (ID 0.3 mm, OD 0.8 mm; Adtech Polymer Engineering Ltd, UK, No. 73692) was connected to both the thick tubing and the inlet of the flow channel. Another piece of thin tubing was used to connect the outlet of the flow channel to a waste container. Importantly, upon connecting the inlet tubing, a small air bubble was forced through the flow channel to remove cells that are not in the chambers. Cells were incubated for 36-48 h at 37 °C before image acquisition, which allowed cells to completely fill the chambers, even for chambers that experienced early washout of cells (i.e., when few cells are in the chambers, cells can swim out).

#### Microscopy

Microscopy was performed on two microscopes: (i) Olympus IX81 inverted microscope with Z-drift compensation v1 (Olympus), ORCA-flash 4.0 v2 sCMOS camera (Hamamatsu, Hamamatsu, Japan), and cellVivo Microscope Incubation System (Pecon GmbH, Germany); (ii) Olympus IX83 inverted microscope systems with Z-drift compensation v2 (Olympus), ORCA-flash 4.0 v4 sCMOS camera (Hamamatsu, Hamamatsu, Japan), and Cube Incubation System (Life Imaging Services, Switzerland). Both microscopes were equipped with a X-Cite120 120 W high-pressure metal halide arc lamp (Lumen Dynamics) along with CFP, Texas Red, and YFP fluorescent filters (Chroma), a motorized stage, automated stage controllers (Marzhauser Wetzlar) and shutters, and a UPFLN ×100 oil immersion objective (Olympus). Microscopes were controlled using the CellSens software.

### Image analysis

#### Data selection, data fitting, and parameter extraction

To determine the communication range, we analyzed all microscopy images with two clearly distinct regions of signal producers and receivers. Only for the PlcR-PapR system, we ignore clusters <3 μm in width, because these showed clear self-sensing (Supplementary Discussion, Section 1.4.5 and Supplementary Figs. 14–16). Cells were segmented using Matlab R2019a (Mathworks, Inc.), using both edge detection and thresholding of the fluorescent signal (RFP for signal receivers and BFP of signal producers). Further data analysis was limited to cells in the back (typically, one-third) of the chamber, where cells experience no flow or washouts. For every cell, we determined the average reporter gene expression, which we log-transformed, and the spatial coordinates of the centroid. These data are included in the manuscript as Supplementary Data 1. The centroid was used to determine the shortest distance to the boundary between signal producers and receivers for every cell. Distances are expressed relative to the boundary and expressed as negative values for signal producers and positive values for signal receivers. This yields a signal response gradient, where YFP expression of cells can be plotted in space along a single dimension (Figs. 2b–d, 3a, and 4). In order to subsequently quantify the communication gradient, we fitted a three-piece using the lsqcurvefit function in Matlab:

$$Y=\left\{\begin{array}{cc}{Y}_{\mathrm{max}} & \qquad x\,\le\, {x}_{\mathrm{min}}\hfill\\ {Y}_{\mathrm{max}}-\cdot \frac{({Y}_{\mathrm{max}}-{Y}_{\mathrm{min}})}{({x}_{\mathrm{max}}-{x}_{\mathrm{min}})}(x-{x}_{\mathrm{min}}) &\qquad {x}_{\mathrm{min}} \,<\, x\, <\, {x}_{\mathrm{max}}\\ {Y}_{\mathrm{min}} & x\,\ge\, {x}_{\mathrm{max}}\end{array}\right.$$

Here, $Y$ is the logarithm of the signal response and $x$ the distance of cells to the boundary of signal producers. We fit ${x}_{{\min }}$, that is, distance from the boundary at which cells show saturated signal response; ${x}_{{\max }}$, that is, distance from the boundary at which cells show no signal response; ${Y}_{{\min }}$, minimal reporter gene expression; and ${Y}_{{\max }}$, maximum reporter gene expression. The response decay length scale (${\lambda }_{\mathrm{response}}$) is given by $\frac{{Y}_{{\max }}-{Y}_{{\min }}}{{x}_{{\max }}-{x}_{{\min }}}$ (see also Supplementary Fig. 1 and Supplementary Discussion, Section 1.3). The communication range is defined as the distance from the boundary of signal producers at which the signal response best fit reduces one order of magnitude on a natural log scale compared to the signal response fit at the boundary. In other words, if there is no saturated signal response in the region of signal receivers (${x}_{{\min }} \, \le \, 0$), the communication range is equal to ${\lambda }_{\mathrm{response}}$. Conversely, when signal receivers do express saturated signal response (${x}_{{\min }} \, > \, 0$), the communication range is equal to ${{x}_{{\min }}+\lambda }_{\mathrm{response}}$ (see Supplementary Figs. 1 and 10). For the signal response gradients in Fig. 3a, where the response is studied in signal receivers only, we fitted a two-piece function, assuming ${x}_{{\min }}=0$. For the mobile genetic elements, we fit the inverse function (see below for details), where the signal response ($Y$) increases as a function of distance ($x$).

To assess the goodness of fit, we performed bootstrapping by resampling the signal response data and fitting the three-piece functions for 50 iterations. Based on these fits, we determine the average fitted response decay length scale, $\langle {\lambda }_{{\mathrm{response}}}\rangle$, and the associated standard deviation, ${\sigma }_{\lambda }$. We only considered images for further analysis when we could obtain robust parameter estimates for the response decay length scale, $\frac{{\sigma }_{\lambda }}{\left\langle {\lambda }_{{\mathrm{response}}}\right\rangle } \, < \, 0.4$. Although the same goodness-of-fit criterion was applied to all data, it mainly affected the analysis of the nonabsorbing ComQXP quorum-sensing system, where the relatively weak signal response gradients (i.e., long communication ranges, Fig. 2b) and sometimes noisy expression data made it difficult to get robust parameters estimates for a fraction of the images. Our estimated communication range in the ComQXP system, therefore, likely forms a slight underestimation of the real communication range (see also below). In contrast, for all the absorbing quorum-sensing systems, which show strong signal response gradients, fits nearly always satisfied our goodness-of-fit criteria. Bootstrapping also affected PlcR data with narrow producer clusters (Supplementary Fig. 14).

#### Cluster size and signal response analysis

In Fig. 3d, we determine the relationship between the average signal response in clusters of signal producers and their size (PlcR-PapR quorum-sensing system). The cluster size is determined by the average width of a cluster along its longitudinal axis, going from the back of a chamber to the front. Since the boundaries of the microfluidic chamber are reflective, clusters adjacent to the left or right side of the chamber will accumulate twice as much signal as clusters of similar size in the middle of the chamber. We, therefore, correct the cluster size for the location of a cluster, where the effective cluster width of clusters adjacent to the chamber side is twice the measured cluster width. The cluster half-width, shown on the *x*-axis in Fig. 3d, is simply half the (effective) cluster width.

Based on a 1D model that approximates our microfluidic setup (see Eq. (45) in Supplementary Discussion, Section 2.1.3), we can theoretically predict the normalized average signal response (${Y}_{{\mathrm{av}}}$) inside a cluster of signal producers (black line in Fig. 3d):

$${Y}_{{\mathrm{av}}}=\frac{\left[1+\frac{\lambda }{2{\rm{R}}}\left({\rm{exp }}\left(-\frac{2R}{\lambda }\right)-1\right)\right]}{\left[1+\frac{\lambda }{2 {{\rm{R}}}_{{\max }}}\left({\rm{exp }}\left(-\frac{2{R}_{{\max }}}{\lambda }\right)-1\right)\right]}$$

Here, $\lambda$ is the communication range, which in the PlcR-PapR system is equal to the response decay length scale (see Fig. 2b and Supplementary Figs. 1 and 10); $R$ is the cluster radius, in Fig. 3d approximated by the cluster half-width. The expected signal response is normalized to the signal response in the largest cluster (${R}_{{\max }}$). Figure 3d reveals that the expected signal response corresponds closely to the observed signal response.

#### The zone of inhibition and conjugation in the ICE*Bs1* system

In contrast to the quorum-sensing systems examined in Fig. 2, where signal molecules induce the expression of a reporter gene, for the quorum-sensing systems of the mobile genetic elements (Fig. 4) signal molecules repress gene expression. The range of communication is therefore determined by fitting the following three-part function to the expression data:

$$Y=\left\{\begin{array}{cc}{Y}_{\mathrm{min}} & x\,\le\, {x}_{\mathrm{min}}\\ {Y}_{\mathrm{min}}+\cdot \frac{({Y}_{\mathrm{max}}-{Y}_{\mathrm{min}})}{({x}_{\mathrm{max}}-{x}_{\mathrm{min}})}(x-{x}_{\mathrm{min}}) &\qquad {x}_{\mathrm{min}} \,<\, x\, <\, {x}_{\mathrm{max}}\\ {Y}_{\mathrm{max}} & x\,\ge\, {x}_{\mathrm{max}}\end{array}\right.$$

Here, $Y$ is reporter gene expression (after log normalization; AU) and $x$ the distance of cells to the boundary of signal producers. The response decay length scale (${\lambda }_{{\mathrm{response}}}$) is given by $\frac{{Y}_{\max}-{Y}_{\min }}{{x}_{\max}-{x}_{\min}}$ (Supplementary Fig. 19a) and the zone of inhibition is equal to ${x}_{{\max }}$ (see also Supplementary Fig. 19b). The zone of inhibition is the spatial distance from the boundary of signal producers over which signal molecules repress the induction of horizontal gene transfer (i.e., reporter gene expression). Cells beyond the zone of inhibition ($x\ge {x}_{{\max }}$) show maximum reporter gene expression—that is, induction of horizontal gene transfer (Fig. 4).

We use the zone of inhibition to define a cell’s neighborhood in the analysis in Fig. 4d. That is, all cells within a radius, ${x}_{{\max }}$, from a focal cell belong to the neighborhood of this cell (using the centroid *xy* coordinates of cells). In Fig. 4d, we examine how the fraction of potential recipient cells in the neighborhood of ICE*Bs1*-containing host cells affects the induction probability of conjugation. We monitor the induction using a reporter gene (YFP expression under the control of the *xis* promoter). Every host cell that expresses the reporter gene at a level 50-fold higher than the median expression is considered to induce conjugation (alternative expression thresholds give very similar results). For the analysis in Fig. 4d, we analyze all host cells in the community, with exception of cells that are 10 μm removed from the chamber outlet, because these cells often undergo abrupt changes in the neighborhood composition due to washout of cells.

### Reporting summary

Further information on research design is available in the Nature Research Reporting Summary linked to this article.

## Supplementary information

Supplementary Information

Description of Additional Supplementary Files

Supplementary Movie 1

Supplementary Movie 2

Supplementary Movie 3

Supplementary Movie 4

Supplementary Movie 5

Supplementary Movie 6

Supplementary Movie 7

Supplementary Data 1

Reporting Summary

## Data Availability

Source data are provided with this paper (Supplementary Data 1).

## Code availability

Matlab scripts are provided with this paper (Supplementary Data 1).
