# Supplementary material for: Short-range quorum sensing controls horizontal gene transfer at micron scale in bacterial communities
Source: Nat Commun. 2021 Apr 19;12:2324. doi: 10.1038/s41467-021-22649-4 (PMC8055654; doi:10.1038/s41467-021-22649-4)
Supplement: Supplementary file 1 — Supplementary Information [file 41467_2021_22649_MOESM1_ESM.pdf]

Supplementary information

Short-range quorum sensing controls horizontal gene transfer at micron scale in bacterial communities

Jordi van Gestel, Tasneem Bareia, Bar Tenennbaum, Alma Dal Co, Polina Guler, Nitzan Aframian, Shani Puyevsky, Ilana Grinberg, Glen G D'Souza, Zohar Erez, Martin Ackermann, Avigdor Eldar.

Contents

Supplementary Figures 4

Supplementary Tables 29

Supplementary Discussion 32

1 Quorum-sensing designs 32

1.1 Absorbing and non-absorbing quorum sensing systems 32

1.1.1 Non-absorbing design. Type A: Extracellular signaling 32

1.1.2 Non-absorbing design. Type B: Intracellular signaling 33

1.1.3 Absorbing design. Type A: Peptide-based irreversible uptake 33

1.1.4 Absorbing design. Type B: Non-peptide-based irreversible uptake 34

1.2 Absorbing and non-absorbing quorum-sensing systems discussed in the main text.... 35

1.3 The relation between the communication range and other spatial measures 37

1.3.1 Linear scenario 37

1.3.2 Non-linear scenario 39

1.4 Empirical considerations 42

1.4.1 Signal concentration is not expected to saturate *opp* uptake system 42

1.4.2 Predicted communication range based on previous parameter estimates 43

1.4.3 Signal-response curves of absorbing quorum-sensing systems 43

1.4.4 Peptide length, signal uptake and communication range 44

1.4.5 Reasons for the extended communication range of small clusters 45

1.5 Alternative mechanisms that could limit signal diffusion in space 46

1.6 The social context of absorbing quorum-sensing systems 47

2 Mathematical modeling of quorum-sensing designs 48

2.1 Signal gradient properties: Analytic solutions for various geometries 48
2.1.1 Three relevant cases 50
2.1.2 Analysis of three-dimensional spherical symmetry 51
2.1.3 Solving case 3 in one and two dimensions 54
2.2 The dependence of quorum-sensing signaling on cell density 56
2.3 Numerical analysis of signal gradient properties in microfluidics chambers 59
2.4 Absorbing systems and the averaging distance of neighbor frequency 60
References 61

Supplementary Figures

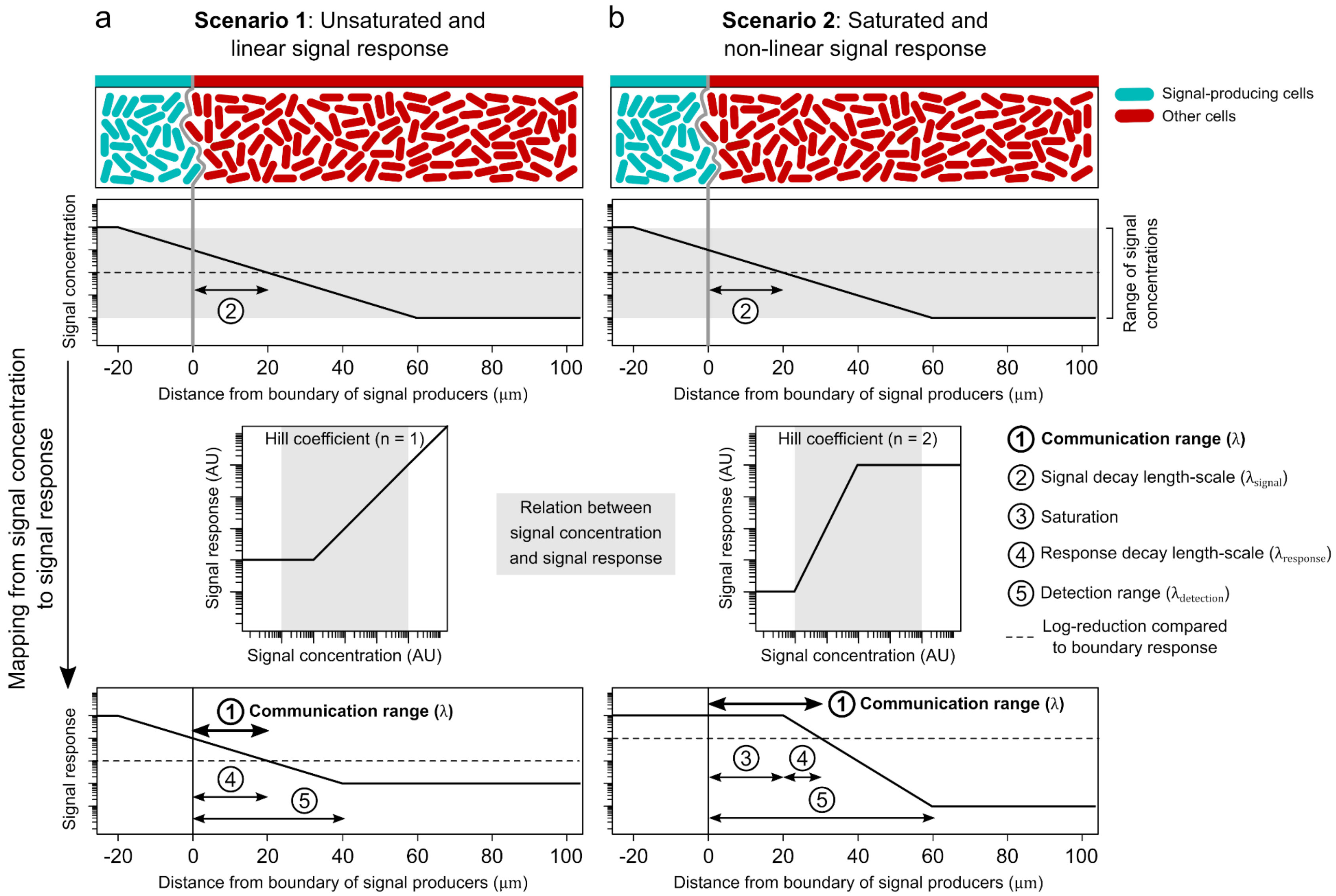

Supplementary Fig. 1. Components that determine communication range in quorum-sensing signaling. From top to bottom: spatial configuration of signal producers (blue) in community of non-producing cells (red); schematic signal concentration gradient in space, measured as distance to boundary of signal producers; signal response curves, showing relation between signal response (i.e. reporter gene expression) and signal concentration; and, finally, signal response as function of the distance of cells to the boundary of signal producers. a, scenario 1: unsaturated and linear signal response. b, scenario 2: saturated and non-linear signal response. Two components together determine the communication range: (1) signal diffusion in space (determined by signal decay length-scale) and (2) signal response (determined by saturated signal response and response decay length-scale). See further analysis in Supplementary Discussion, section 1.3.

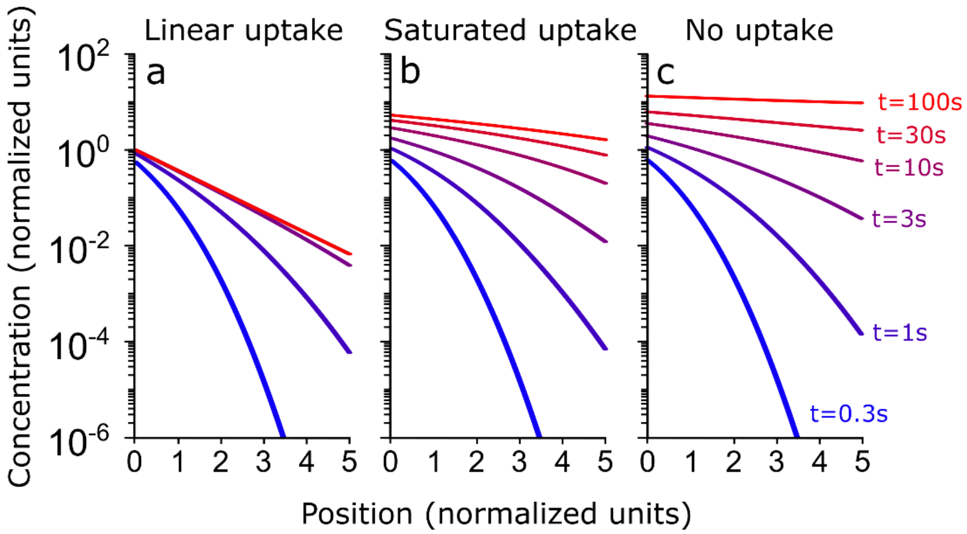

Supplementary Fig. 2. The impact of saturated uptake. Signal concentration as function of distance to boundary for (a) linear signal uptake (absorbing system), (b) saturated signal uptake (absorbing system) and (c) no signal uptake (non-absorbing quorum-sensing system). Different colors mark different times after the initiation of signal production. The signal dynamics follow equation [1], with different functional forms for the uptake rate: (a) constant uptake $\alpha_c = 1$; (b) saturated uptake, $\alpha_c = \frac{1}{0.1+c}$; (c) no uptake, $\alpha_c = 0$. All equations are solved with a constant diffusion rate, $D = 1$ and constant production rate, $\eta = 1$ and a zero flux boundary condition at $x = 10$. Some of lines in panel (a) are covered, as steady-state is reached earlier for linear signal uptake, due to which some of the lines overlap.

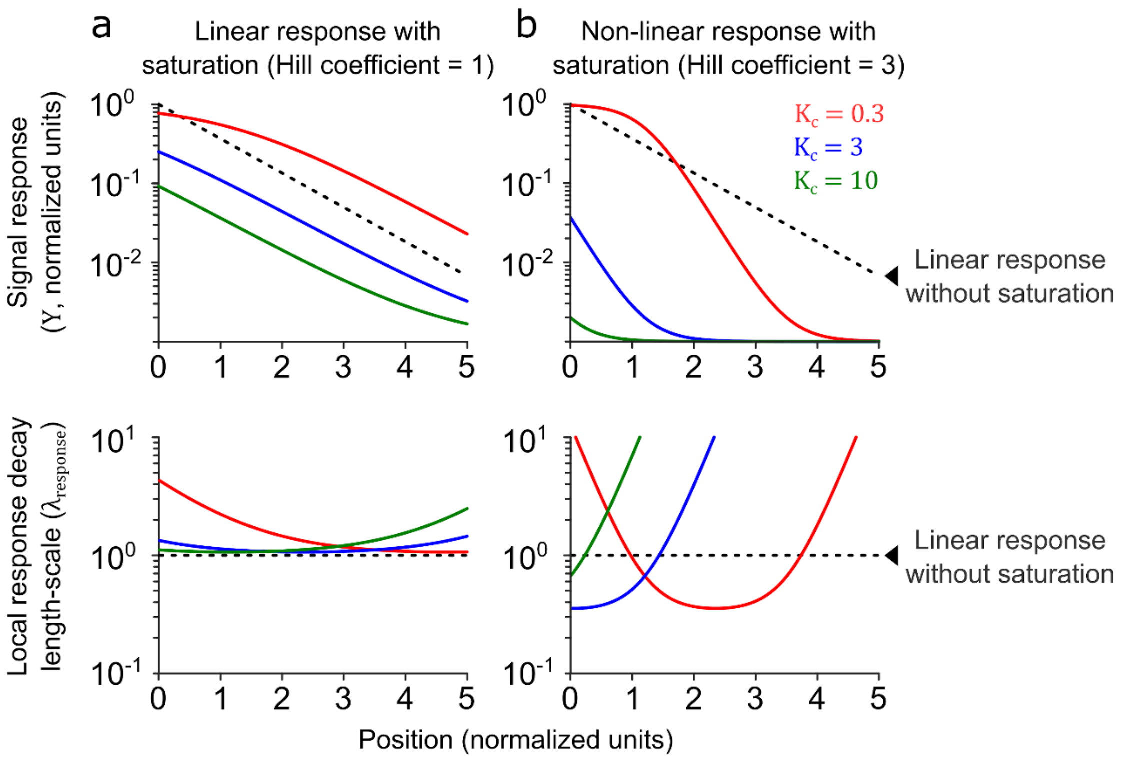

Supplementary Fig. 3. Impact of saturated and non-linear signal response. Response profiles (top panels) and local response decay length-scales (bottom panels) for an exponentially declining signal, $c = \exp(-x)$, and for different signal-response curves following equation [9]: (a) linear response (Hill coefficient, $n = 1$) and (b) non-linear response (Hill coefficient, $n = 3$) with $K_c = 0.3,3,10$ in red, blue and green lines respectively. For both (a) and (b), $Y_{min} = 0.001$ and $Y_{max} = 1$. Dashed line shows (top) signal response and (bottom) local response decay length-scale for linear unsaturated signal-response curve. Both close and far from the boundary of signal producers (i.e. high and low signal concentration), the local response decay length-scale of the non-linear and saturated signal response is higher than that of the linear signal response. At intermediate distances (i.e. intermediate signal concentrations), the non-linear response decay length-scale is lower than the linear response-decay length-scale, and approximately $1/n$.

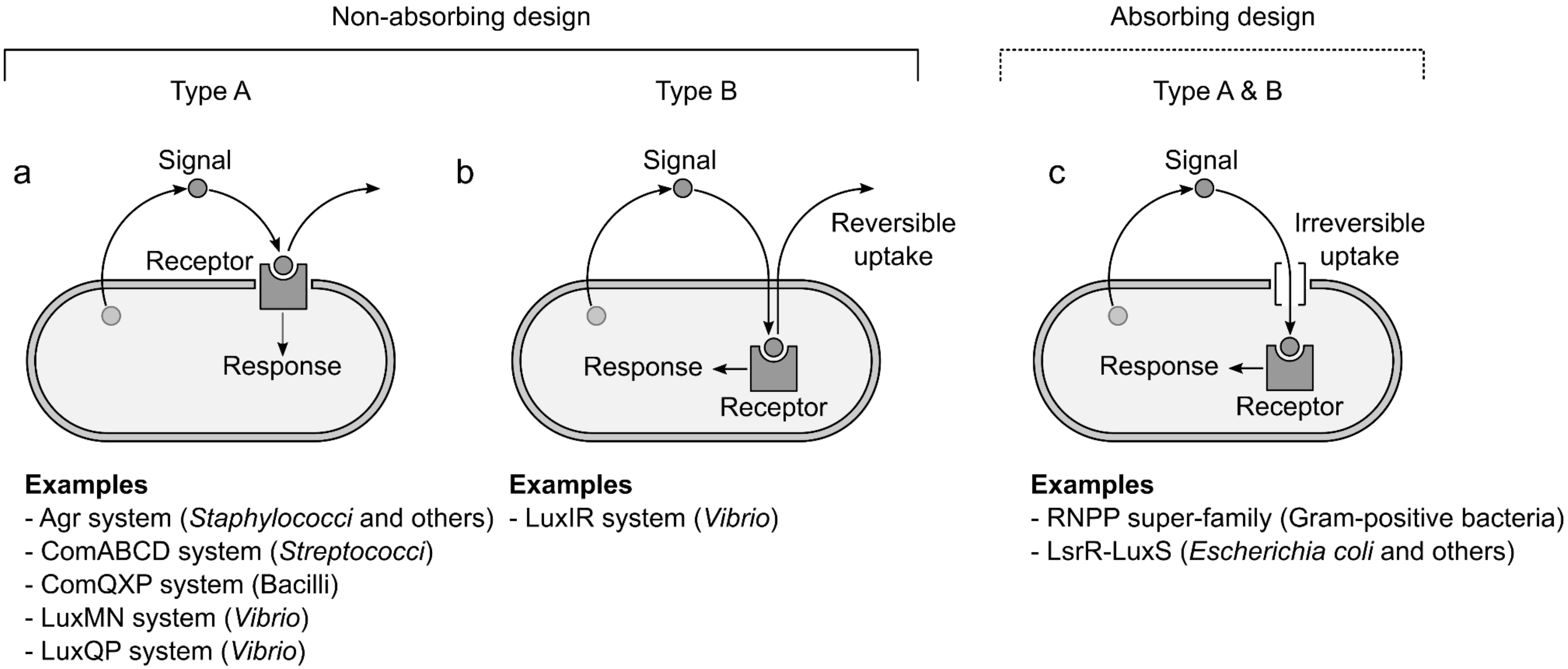

Supplementary Fig. 4. Schematic overview of absorbing and non-absorbing quorum-sensing designs. a, type A non-absorbing design with extracellular signaling. b, type B non-absorbing system with intracellular signaling and reversible signal uptake. c, absorbing quorum-sensing system with intracellular signaling and irreversible signal uptake. This category is further subdivided depending on whether the signal molecule is a small peptide (type A) or not (type B). Some well-studied families of each category are mentioned below the schemes. See Supplementary Discussion, section 1.1 for more details.

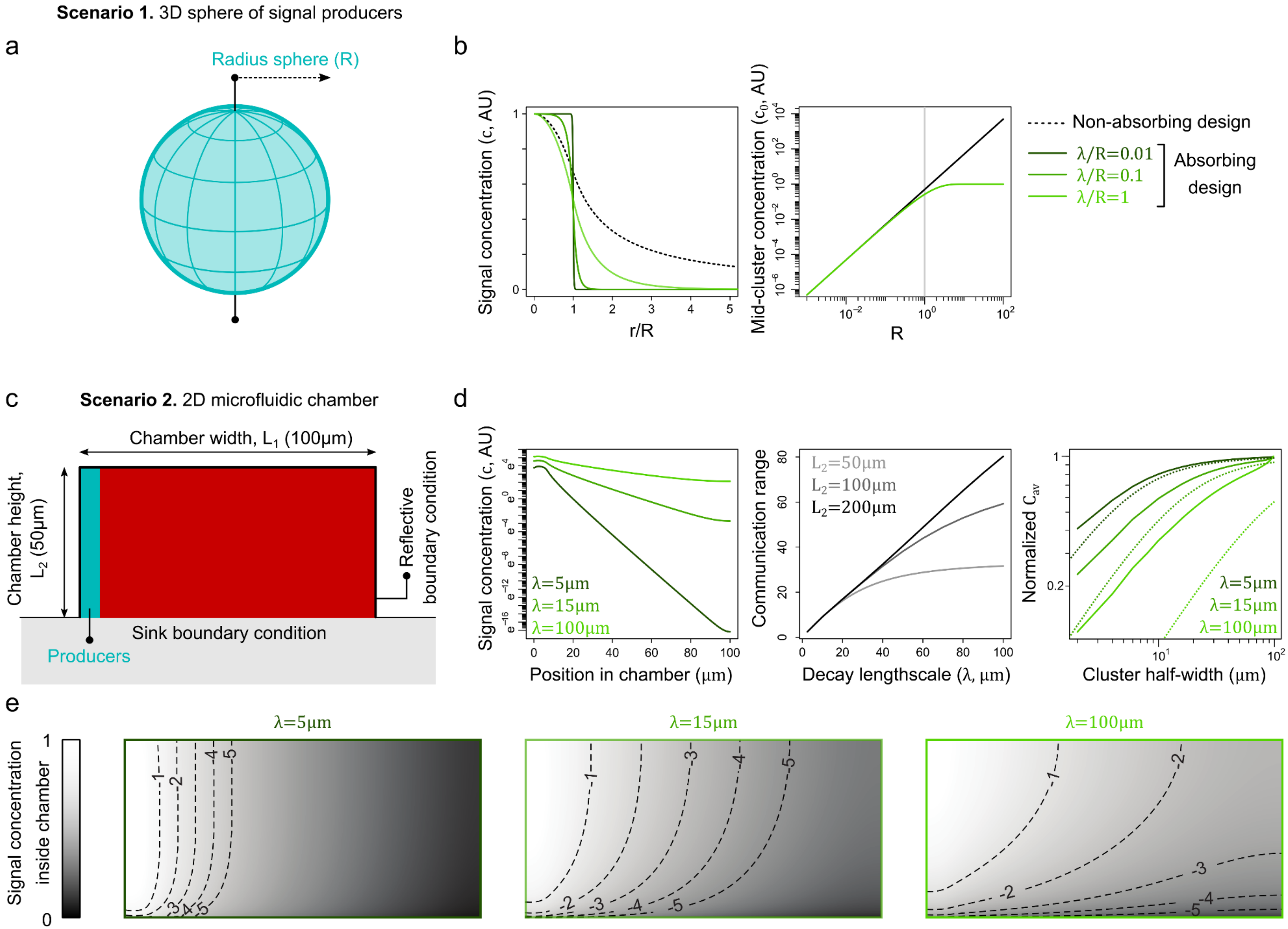

Scenario 1. 3D sphere of signal producers
a
Radius sphere (R)
b
Signal concentration (c, AU)
r/R
Mid-cluster concentration (c0, AU)
R
Non-absorbing design
λ/R=0.01
λ/R=0.1
λ/R=1
Absorbing design
c
Scenario 2. 2D microfluidic chamber
Chamber width, L1 (100μm)
Chamber height, L2 (50μm)
Reflective boundary condition
Sink boundary condition
Producers
d
Signal concentration (c, AU)
λ=5μm
λ=15μm
λ=100μm
Position in chamber (μm)
Communication range
L2=50μm
L2=100μm
L2=200μm
Decay lengthscale (λ, μm)
Normalized Cav
Cluster half-width (μm)
e
Signal concentration inside chamber
λ=5μm
λ=15μm
λ=100μm

Supplementary Fig. 5. Model predictions on communication ranges of absorbing and non-absorbing quorum-sensing designs. a, schematic depiction of model scenario 1: 3D spherical cluster of signal producers embedded in a community of cells that do not produce signal (not shown). b, left, signal concentration as a function of the distance (r) to the cluster center (normalized to cluster radius, R). b, right, concentration in cluster center (c_0) as function of cluster size (R, radius). Vertical grey line indicates communication range. For the absorbing design, we assume that the decay length-scale is $\lambda = 1$. Both absorbing and non-absorbing systems behave as $c_0 \propto R^2$ for $R \ll \lambda$, but absorbing systems saturate for $R \gg \lambda$. c, schematic depiction of model scenario 2: two dimensional community ($L_1 \times L_2 = 100\mu m \times 50\mu m$) resembling a microfluidic growth chamber, with a small zone of signal producing cells (blue) and a large zone of cells that do not produce the signal (red). d, left, signal concentration gradient (c) in the back of the chamber. d, middle, correlation between signal decay length-scale (λ) and measured communication range for different chamber dimensions (varying chamber height, L_1). d, right, average signal concentration in clusters (c_{av}), normalized to the signal concentration in the largest cluster, for clusters of varying size (i.e. half-width). Dotted lines show predicted normalized c_{av} in clusters of signal producers based on 1D model. e, signal concentration gradient inside chamber for quorum-sensing systems with different signal decay length-scales (λ). Contours mark natural log reductions from the maximal signal concentration inside the chamber. We use the Matlab PDE toolbox to solve the diffusion equation with production in the production region and decay in the entire chamber. For more details, see Supplementary Discussion, section 2.

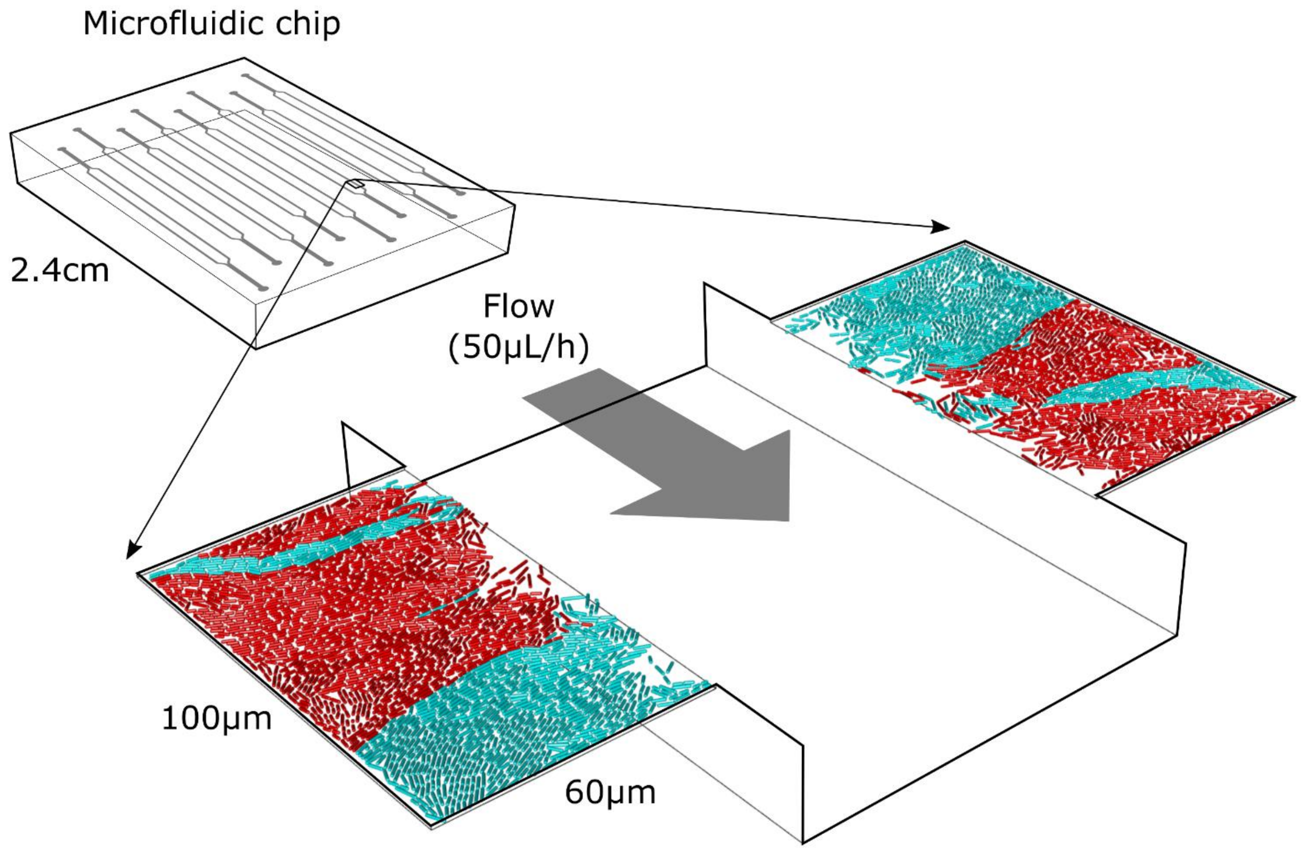

Supplementary Fig. 6. Schematic depiction of microfluidic chip. The microfluidic chip contains eight main channels that, from inlet to outlet, are 2.4 cm long. Each channel splits in two smaller parallel channels (100µm in width and 22µm in depth), from which microfluidic chambers (100µm × 60µm × 0.83µm) expand at the lateral sides. The chambers and connected channel are shown at correct proportions.

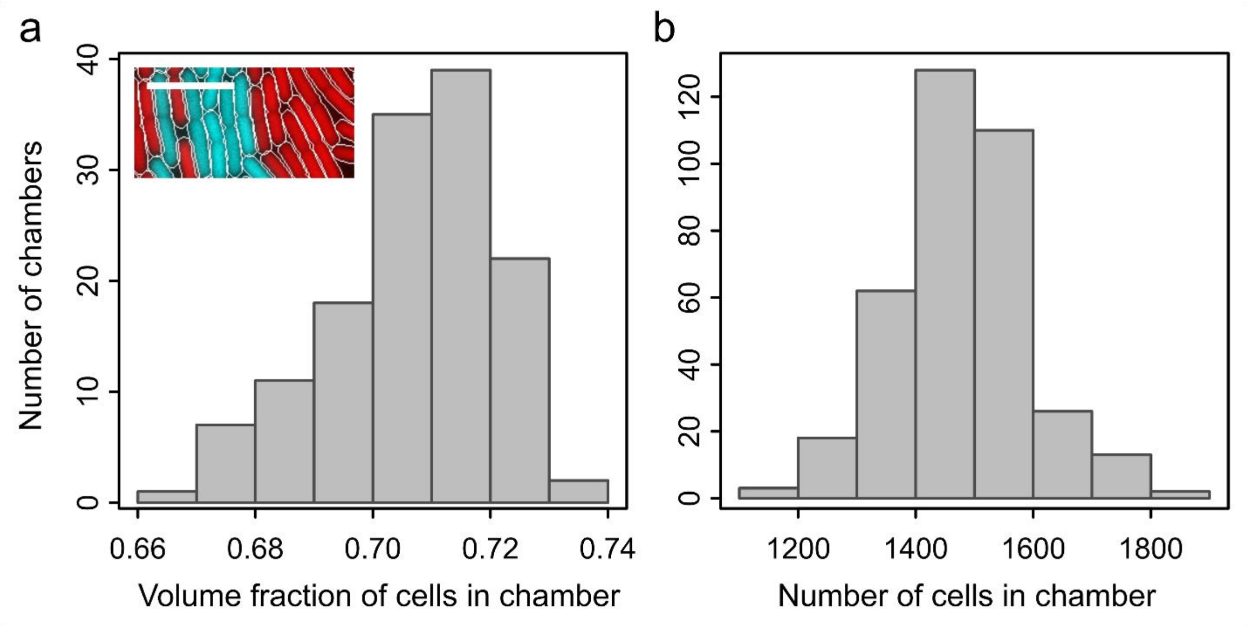

Supplementary Fig. 7. Volume fraction and number of cells inside chambers. a, volume fraction of cells inside chamber ($n = 135$ chambers), as derived from the cell outlines, assuming that parallel cells contact each other. Inset, cell outlines (white) of signal producing (blue) and receiving cells (red). Scale bar is $5\mu m$. b, number of cells in chamber ($n = 362$ chambers).

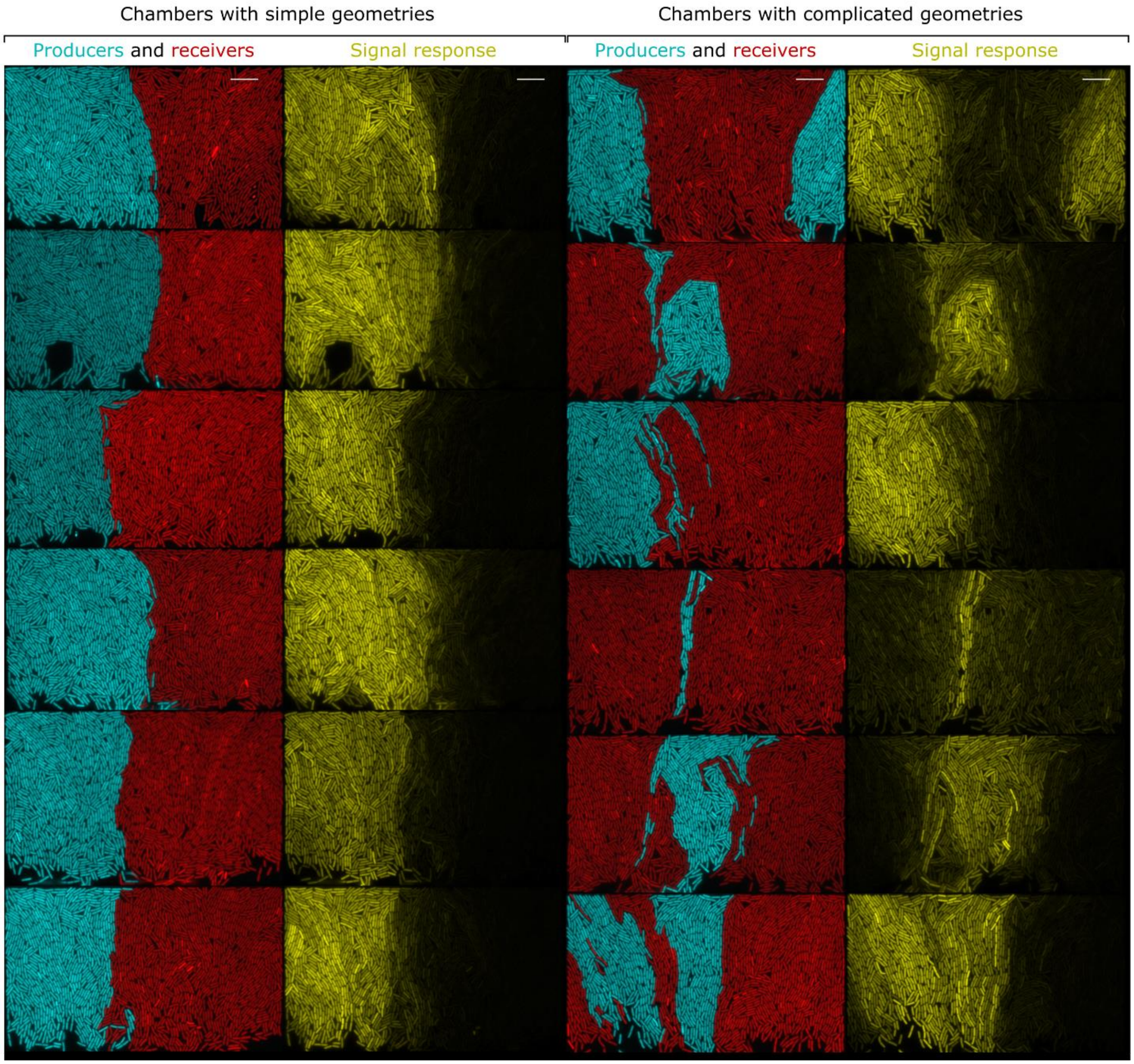

Supplementary Fig. 8. Spatial geometries of signal producing and receiving genotypes in chamber. Spatial geometries of signal producing (blue) and receiving (red) cells inside chamber and corresponding signal response (YFP expression) across chamber in PlcR-PapR quorum-sensing system. Left chambers show simple spatial geometries and right images show more complicated geometries. In all images, color contrasts are maximized, so signal response cannot be compared across chambers. Scale bar = $10\mu m$. Microscopy images show representative results from 14 independent flow channels.

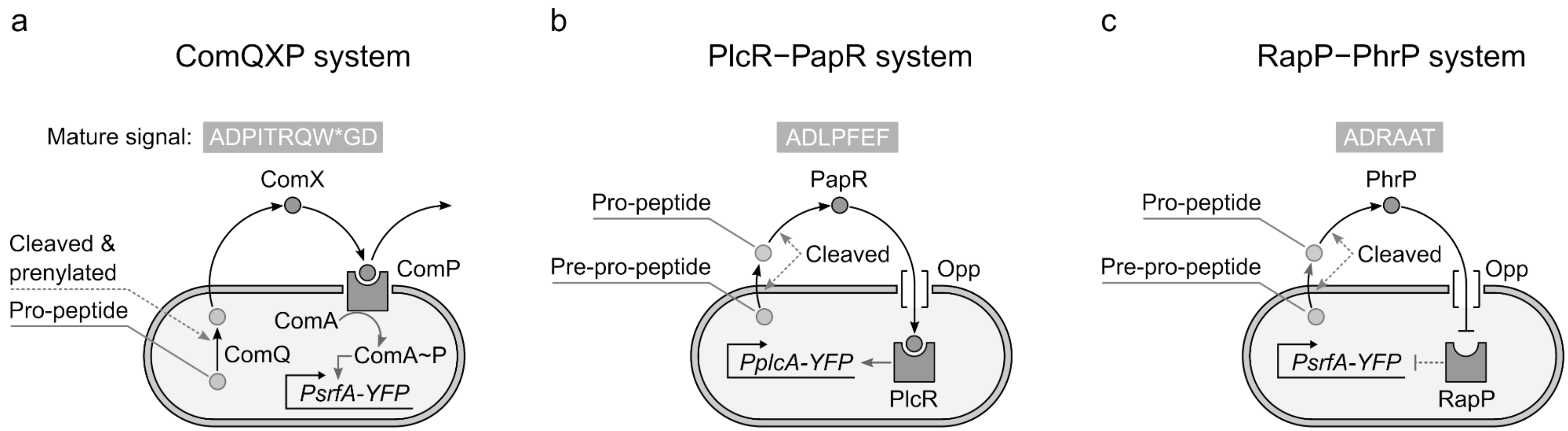

Supplementary Fig. 9. Detailed representation of absorbing and non-absorbing quorum-sensing systems examined in main text. From left to right: (a) ComQXP system, (b) PlcR-PapR system and (c) RapP-PhrP system. Grey boxes show amino-acid sequences of mature signal molecules (* = prenyl modification) Dotted line in (c) show indirect regulatory interaction. For more details, see Supplementary Discussion, section 1.2.

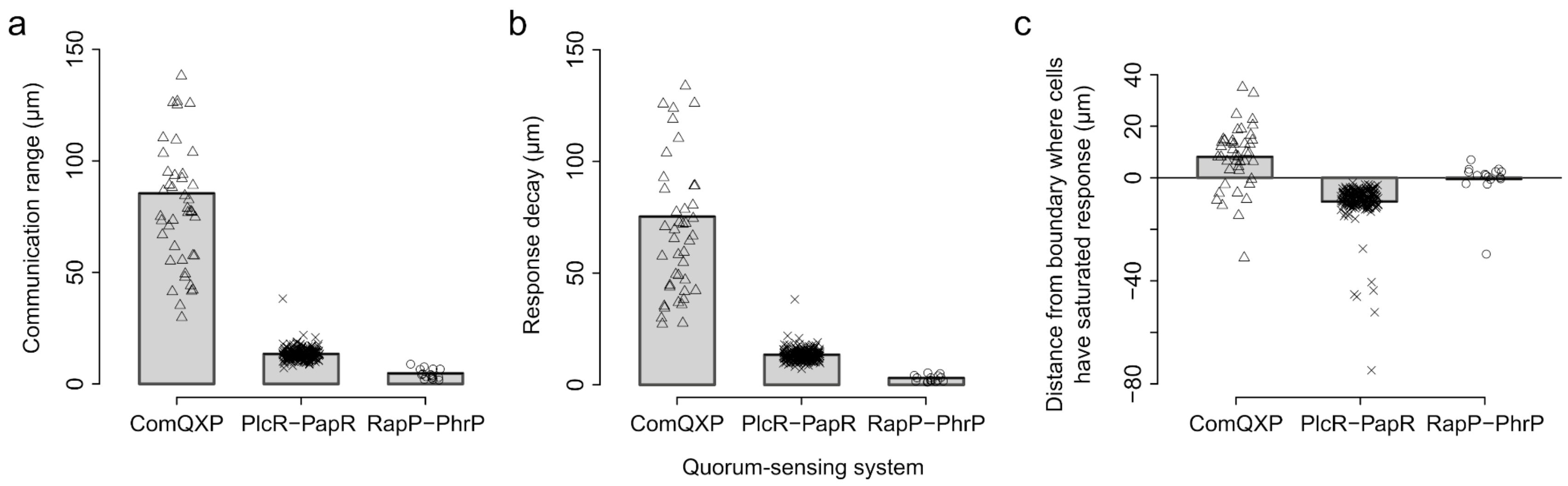

Supplementary Fig. 10. Communication ranges, response decay and saturated signal response in quorum-sensing systems. From left to right, (a) communication range, (b) response decay and (c) saturated signal response in ComQXP, PlcR-PapR and RapP-PhrP systems. Panel (a) is identical to Fig. 2E in main text. (c) ComQXP and RapP-PhrP systems show saturated signal response at boundary between signal producers and receivers, while the PlcR-PapR system does not. As a result, the communication range (a) and response decay (b) in the PlcR-PapR system are the same, while this is not the case for the other two quorum-sensing systems. See Supplementary Fig. 1 for a detailed overview of the different components that determine the communication range.

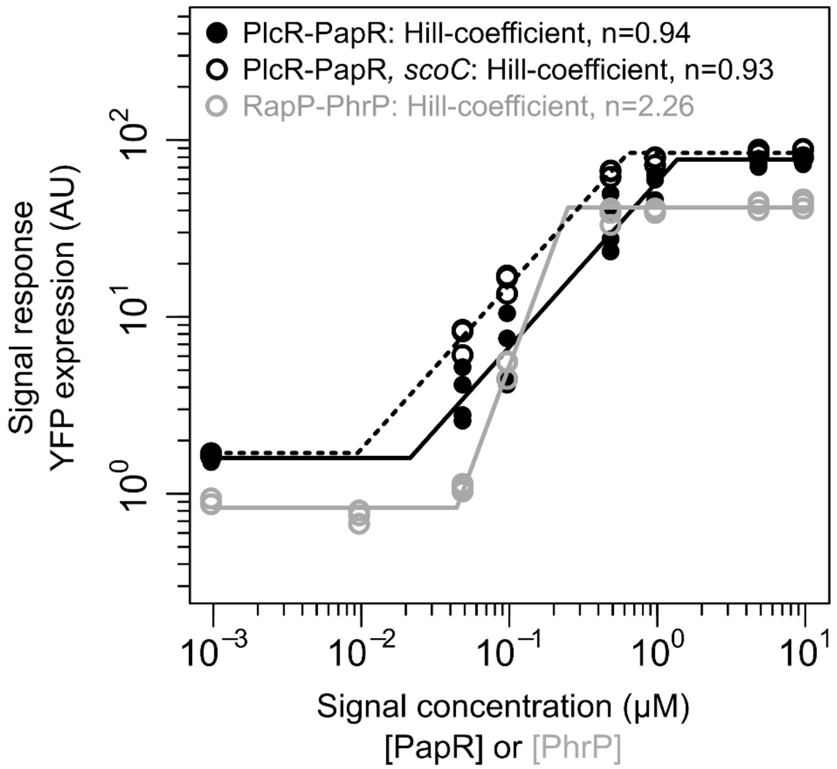

Supplementary Fig. 11. Relation between signal concentration and signal response for absorbing quorum-sensing systems. Black, signal response in PlcR-PapR quorum-sensing system for wildtype (closed circles, black solid line) and *scoC* knockout mutation (open circles, black dashed line). Grey, signal response in RapP-PhrP quorum-sensing system (grey line). Data points show signal response for each quorum-sensing system, including four replicates for each signal concentration, and lines show best fitted curves (Methods).

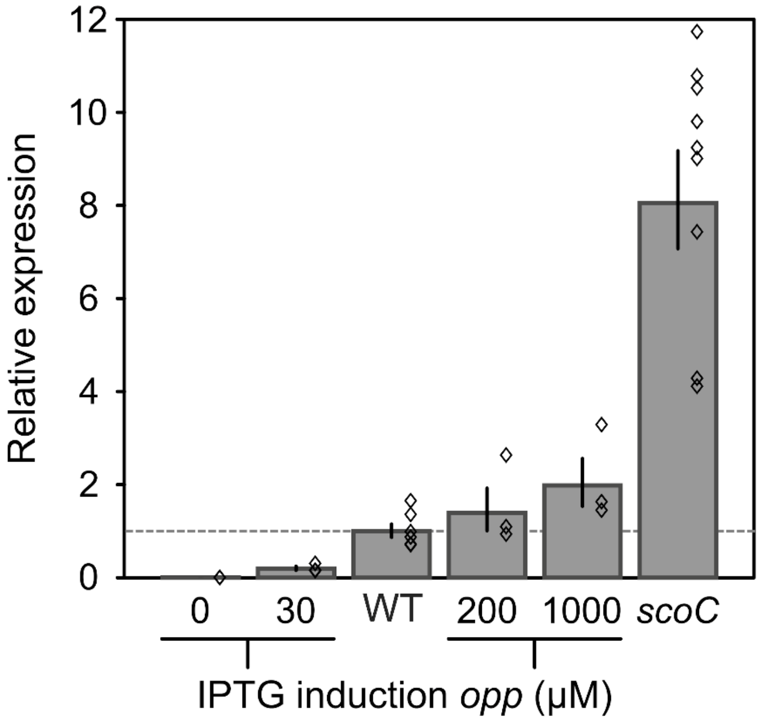

Supplementary Fig. 12. Relative *opp* expression across strains quantified using RT-qPCR. Relative *opp* expression in wildtype, IPTG-inducible *opp* strain and *scoC* mutant strain. Strains are ordered with regard to *opp* expression, where *scoC* knockout mutation has highest expression and the wildtype expression is normalized to 1. Wild type expression is intermediate between 30 and 200µM IPTG in the IPTG-inducible strain. Grey bars show average expression and whiskers show standard error ($n = 3$ replicates for IPTG-inducible *opp* strain, $n \geq 6$ replicates for the rest). RT-qPCR results were normalized with respect to housekeeping gene *rpoB*.

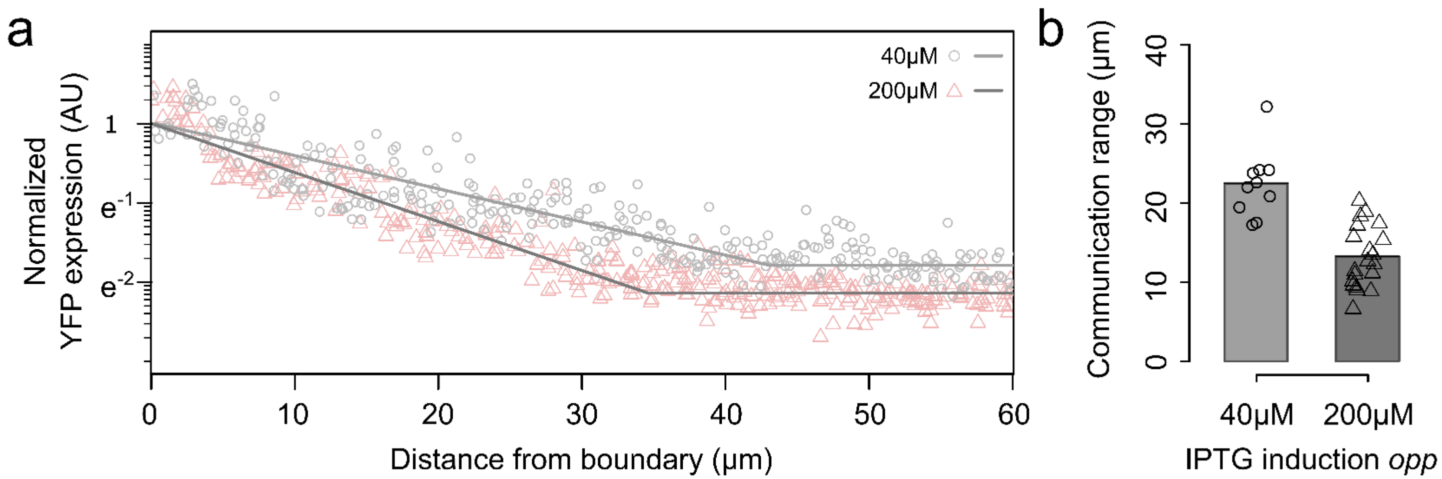

Supplementary Fig. 13. Communication range of PlcR-PapR absorbing system with low *opp* expression. In Figure 3a-b, we show how increased uptake rates decrease the communication range of the PlcR-PapR system, using a co-culture of a constitutive signal producer strain and an IPTG-inducible *opp* receiver strain. We could not use the same system to also explore the effect of low uptake rates, because this resulted in too weak signal response (YFP expression) to robustly quantify the communication range. Here, we overcome this issue by co-culturing an IPTG-inducible *opp* receiver with a strong signal producer (having *papR* expression under the control of a hyperspank promoter without LacI repressor, see strain list), thereby increasing the overall signal concentration and thus the signal response (i.e. YFP expression). Low signal uptake ($40\mu M$) in this new co-culture system results in communication ranges that are significantly lower than the intermediate signal uptake rate ($200\mu M$) examined in Figure 3b ($U = 9, p < 10^{-5}$). a, example of YFP expression gradient from boundary between signal producers and receivers. YFP expression at the boundary is normalized to 1 in both cases. b, communication range at low ($40\mu M$, $n = 11$ chambers from 3 flow channels) and intermediate ($200\mu M$, $n = 20$ chambers from 3 flow channels) induction of *opp* expression (for this analysis, we only compared images with same dynamical range in signal response, to prevent biases that could result from low signal concentrations, see Supplementary Fig. 14).

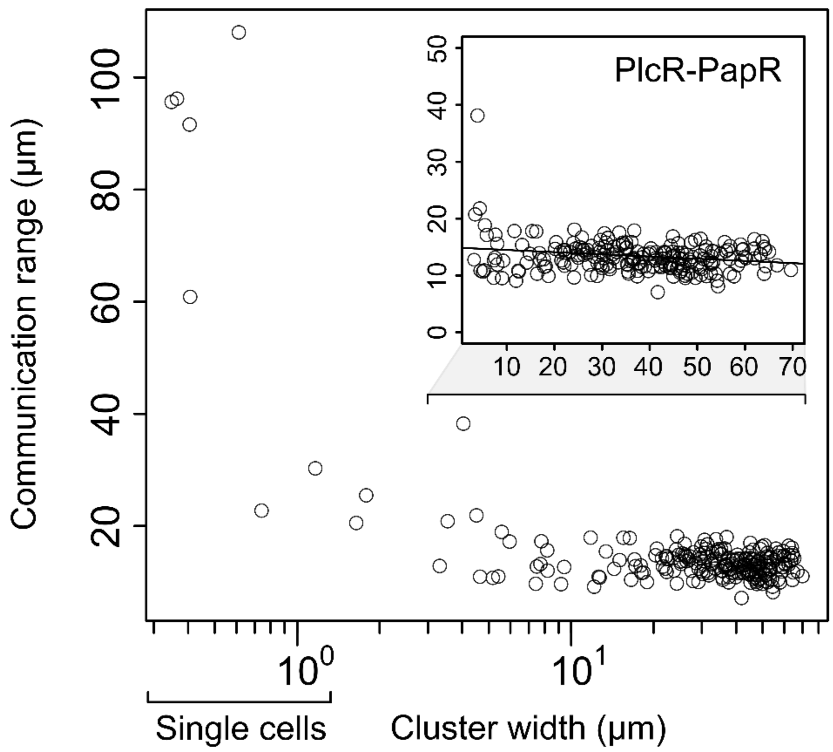

Supplementary Fig. 14. Relation between width of signal producing cluster and measured communication ranges in PlcR-PapR system. Data points show width and communication ranges from isolated clusters of signal producers, surrounded by signal receivers only ($n = 222$ clusters). Inset shows data from corresponding Fig. 3f.

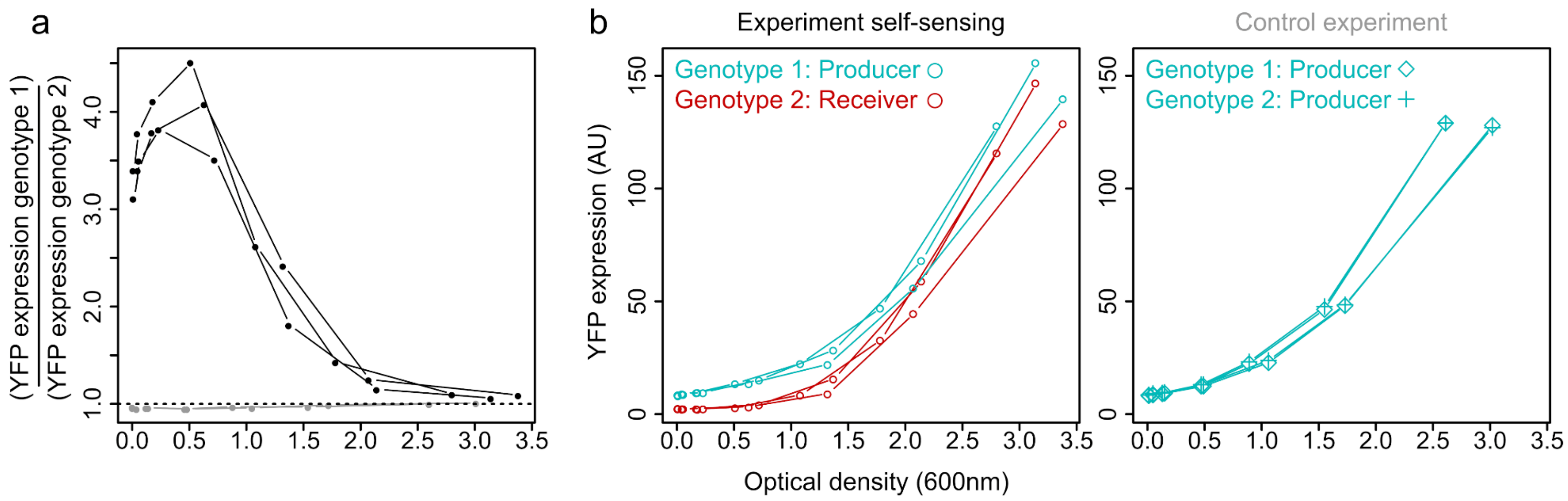

Supplementary Fig. 15. Self-sensing in PlcR-PapR system. a, relative signal response (i.e. YFP expression) of co-cultured genotypes during growth, plotted against the optical cell density. In treatment condition (black lines), genotype 1 is signal producer and genotype 2 is a signal receiver ($n = 3$). In control treatment (grey lines), both genotypes are signal producers ($n = 3$). Dotted horizontal line indicates equal signal response in both genotypes. Signal producing cells show stronger signal response than signal receiving cells, which indicates self-sensing. b, left, absolute signal response of signal producer (blue) and signal receiver (red). b, right, absolute signal response of two signal producer genotypes in control experiment. Note that in control experiment, signal-producing strains carry different fluorescent markers to discriminate them, but are here both shown in blue to indicate that these genotypes produce signal. In all cases, YFP expression is measured using flow cytometry, showing single-cell signal responses.

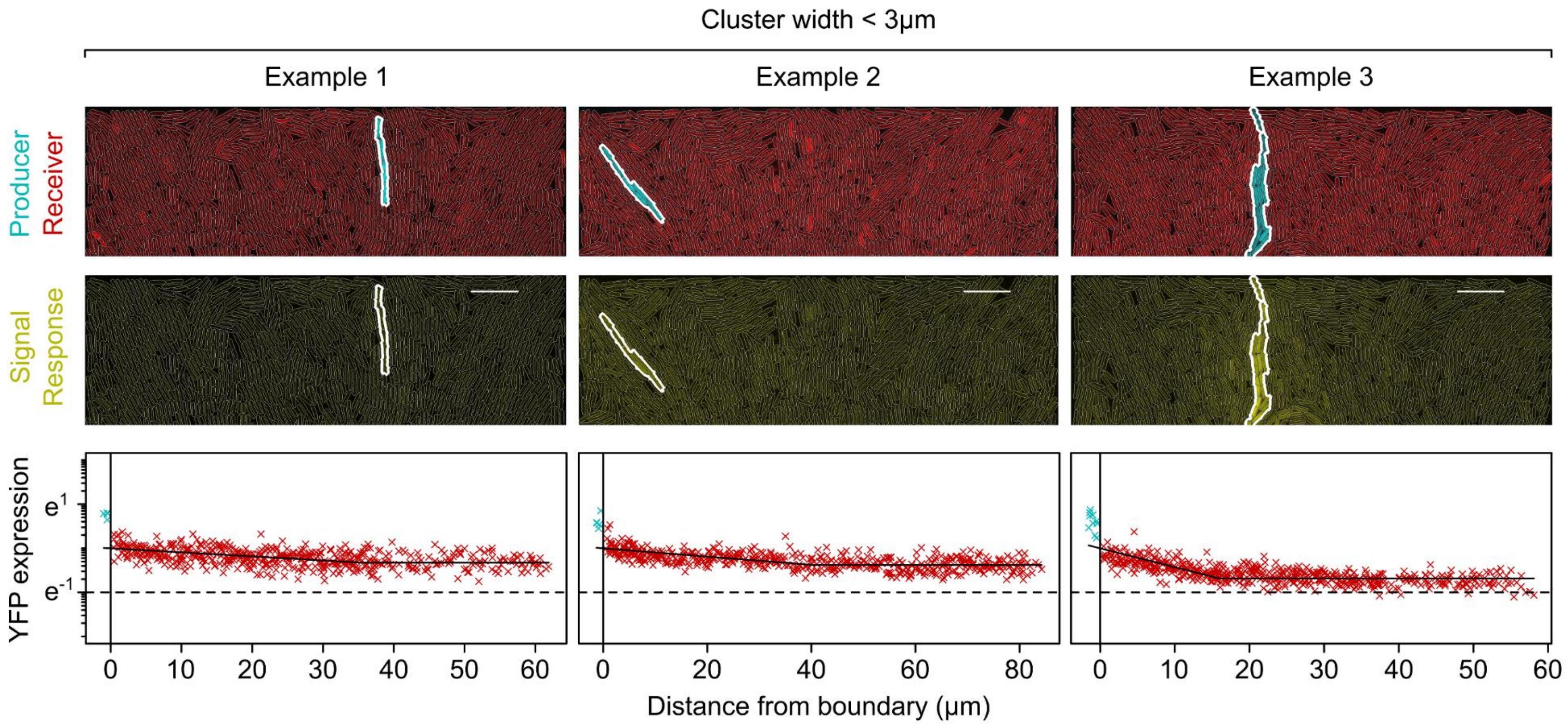

Supplementary Fig. 16. Self-sensing in PlcR-PapR quorum-sensing system in microfluidic chambers. Three examples of self-sensing in narrow clusters ($< 3\mu m$) of signal-producing cells. From top to bottom: microscopy images with distribution of signal producers (blue) and signal receivers (red) in microfluidic chamber (bold white line shows boundary between signal producers and receivers); signal response across chamber (i.e. YFP expression, AU); and, signal response of cells as a function of the distance of cells to the boundary of signal producers. Signal producing cells are marked in blue with negative distances, while receivers are marked in red with positive distances. Scale bar = $10\mu m$. Microscopy images show representative results for chambers with small clusters of signal-producing cells acquired from 14 independent flow channels.

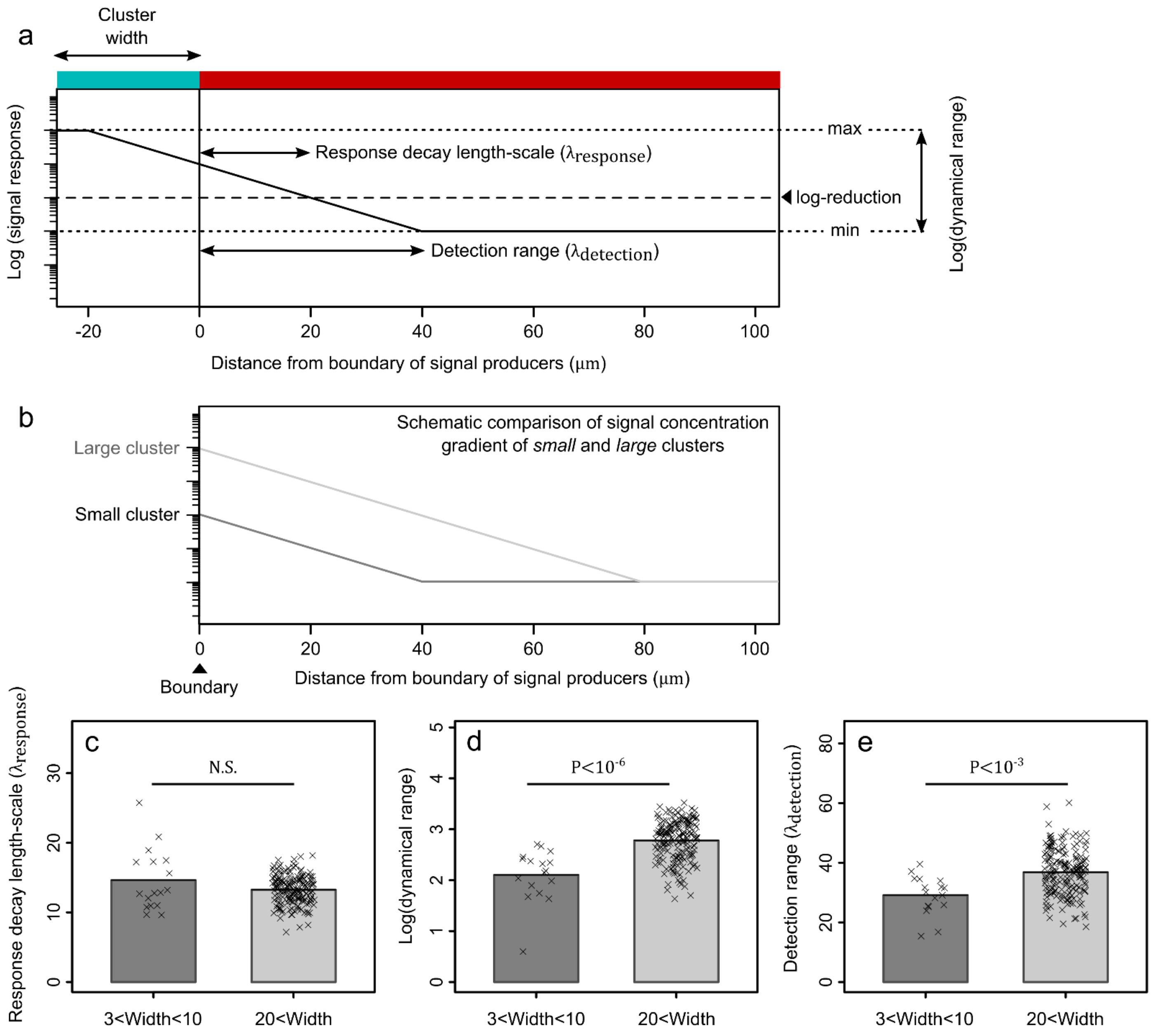
a
Cluster width
Log (signal response)
max
Response decay length-scale ($\lambda_{response}$)
log-reduction
min
Detection range ($\lambda_{detection}$)
Log(dynamical range)
-20
0
20
40
60
80
100
Distance from boundary of signal producers (μm)
b
Schematic comparison of signal concentration gradient of *small* and *large* clusters
Large cluster
Small cluster
0
20
40
60
80
100
Boundary
Distance from boundary of signal producers (μm)
c
N.S.
Response decay length-scale ($\lambda_{response}$)
0
10
20
30
3<Width<10
20<Width
d
$P<10^{-6}$
Log(dynamical range)
0
1
2
3
4
5
3<Width<10
20<Width
e
$P<10^{-3}$
Detection range ($\lambda_{detection}$)
0
20
40
60
80
3<Width<10
20<Width

Supplementary Fig. 17. Higher signal production in larger clusters result in larger detection range. a, distinction between signal response decay length-scale (one log reduction in signal response) and detection range (maximum distance from boundary with signal producers with above-background signal response). Dynamical range shows log-difference between lowest and highest signal response. b, a schematic description of expected signal response in receiving cells next to a small or large cluster of signal producers. The response decay length-scale is unaffected by cluster size (i.e. slope is the same), but the detection range is expected to increase with cluster size, since the signal concentration is higher at the boundary between signal producers and receivers. c-e, comparison of signal response in receivers next to small ($3\mu m < Width < 10\mu m; n = 17$) or large clusters ($Width > 20\mu m; n = 180$) of signal producers: (c) response decay length-scale (smaller cluster, mean=$14.6\mu m$; large clusters, mean=$13.25\mu m$; $U = 1665, p = 0.55$); (d) dynamical range (small clusters, mean = 2.1 logs; large clusters, mean = 2.7 logs; $U = 387, p = 3.7 \cdot 10^{-7}$), (e) detection range (small clusters, mean = $29.1\mu m$; large clusters, mean = $36.8\mu m$; $U = 964, p = 2.0 \cdot 10^{-4}$). Statistics show two-sided Mann-Whitney U tests without adjustments for multiple comparisons.

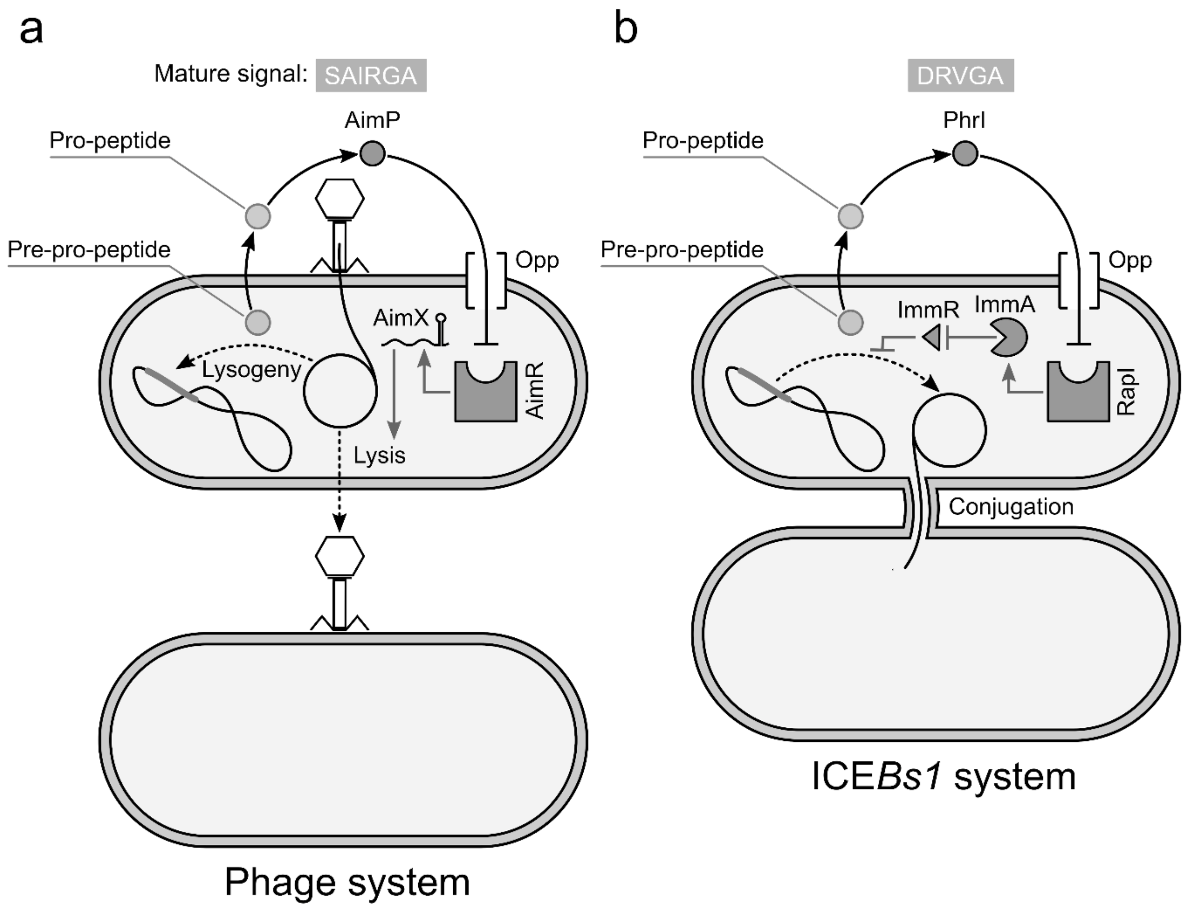

Supplementary Fig. 18. Detailed representation of absorbing quorum-sensing systems of mobile genetic elements. Schematic depiction of quorum-sensing systems in (a) ϕ3T phage and (b) ICE*Bs1* system. Grey boxes show amino-acid sequences of mature signal molecules. For more details see Supplementary Discussion, section 1.2.

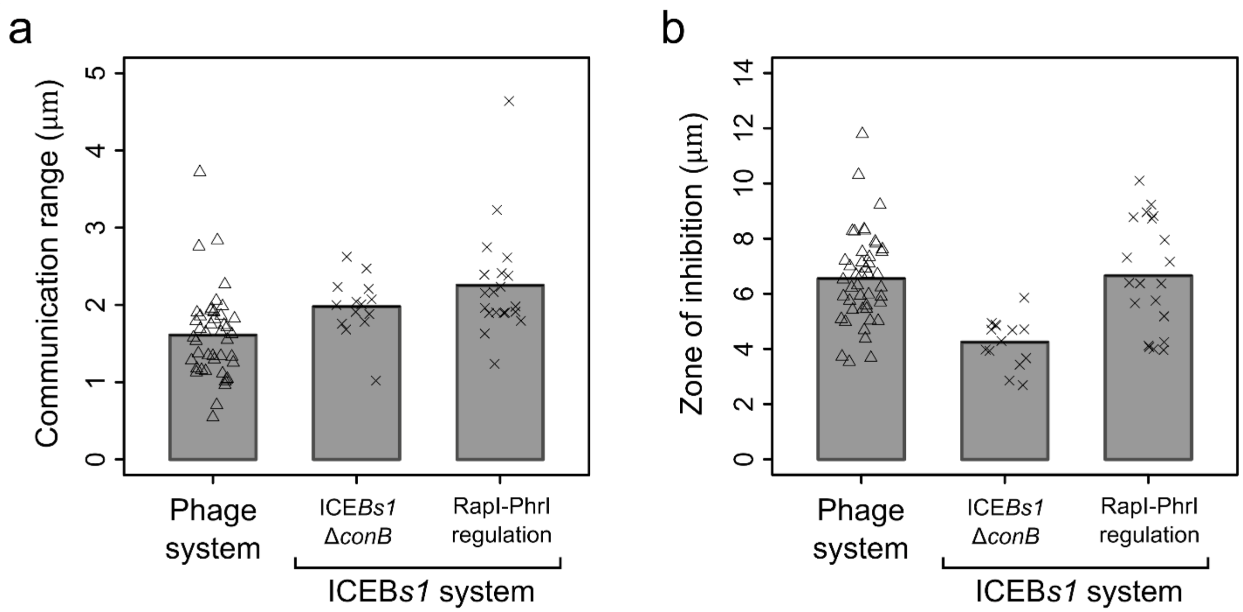

Supplementary Fig. 19. Communication range and zone of inhibition in quorum-sensing systems of mobile genetic elements. (a) Communication range and (b) zone of inhibition in ϕ3T phage ($n = 45$), Δ*conB* ICE*Bs1* ($n = 10$) and regulation-only ICE*Bs1* systems ($n = 15$). The regulatory-only ICE*Bs1* system encodes for the regulatory genes that control the induction of conjugation (*rapI* and *phrI* encoding for the quorum-sensing system and *immA* and *immR* encoding for the downstream regulatory proteins that control conjugation; see also Supplementary Fig. 18 and Methods). Communication range is determined as shown in Supplementary Fig. 1, using one natural log reduction in repression (i.e. increase in YFP expression). Zone of inhibition is defined by the distance from the boundary of signal producers where repression stops and receivers show maximized signal response, based on the best-fit curves (see Fig. 4 and Methods).

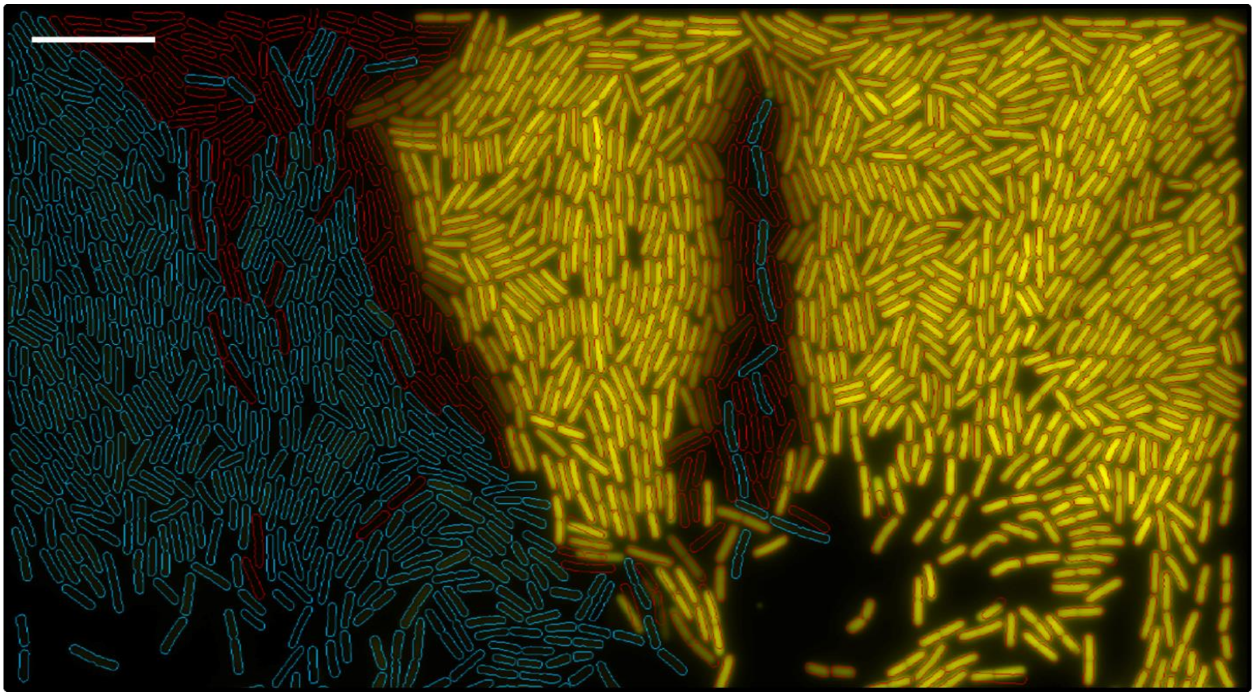

Supplementary Fig. 20. Repression of lytic lifestyle in ϕ3T phage around single cells of signal producers. Signal producing (blue outlines) and signal receiving (red outlines) cells of the arbitrium quorum sensing system. YFP indicates *aimX*-GFP expression and, thereby, reports induction of horizontal gene transfer. Producer cells that occur in isolation and are only surrounded by receiver cells already accumulate sufficient signal molecules (AimP) to suppress horizontal gene transfer in their vicinity. In other words, the cell density in our microfluidic chambers is too high for the ϕ3T phage to spread (in contrast to the ICE*Bs1* system that does induce conjugate in our microfluidic chambers; see Fig. 4). This microscopy image shows representative chamber from chambers imaged in two independent flow channels. Scale bar = 10µm.

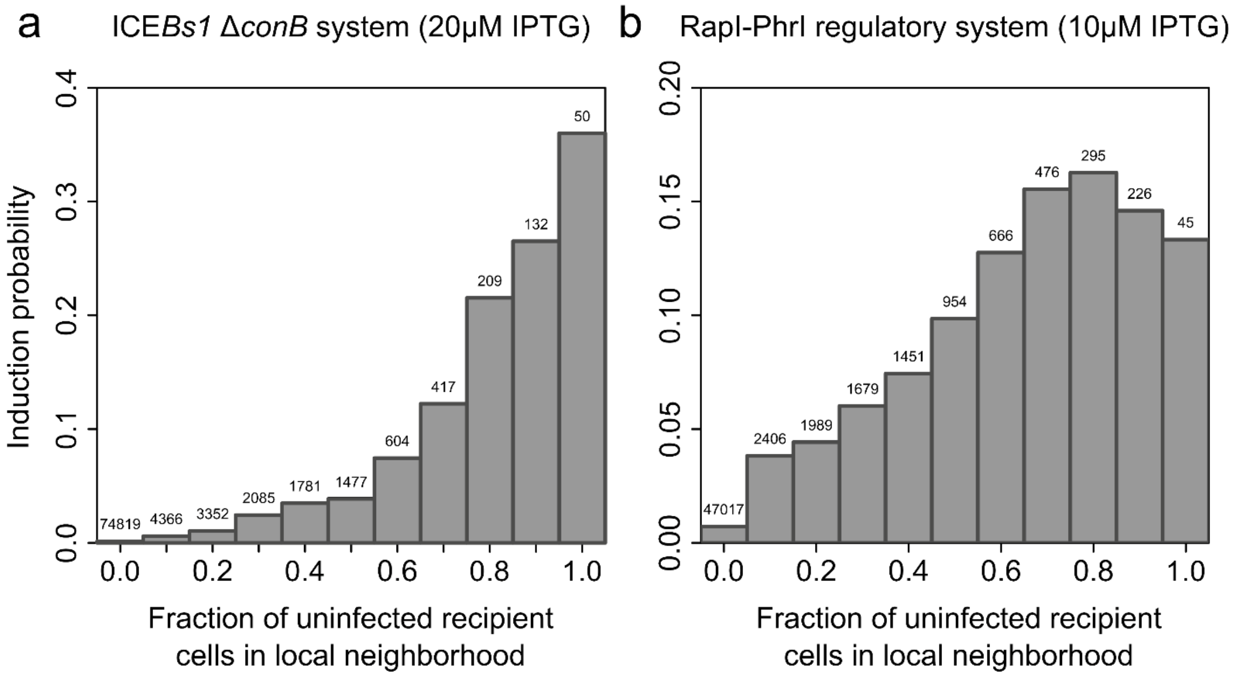

Supplementary Fig. 21. Induction probability of conjugation in ICE*Bs1* system. Induction probability of conjugation in relation to the fraction of uninfected recipient cells in neighborhood for (a) Δ*conB* ICE*Bs1* system and (b) regulatory-only ICE*Bs1* system. The regulatory-only ICE*Bs1* system only encodes for the regulatory genes that control the induction of conjugation (*rapI* and *phrI* encoding for the quorum-sensing system and *immA* and *immR* encoding for the downstream regulatory proteins that control conjugation; see also Supplementary Fig. 18 and Methods). The regulatory-only ICE*Bs1* shows the same trend in the induction probability as the Δ*conB* ICE*Bs1* system (see also Fig. 4d). Numbers above the grey bars show how many ICE*Bs1*-contiaining host cells fall into each bin.

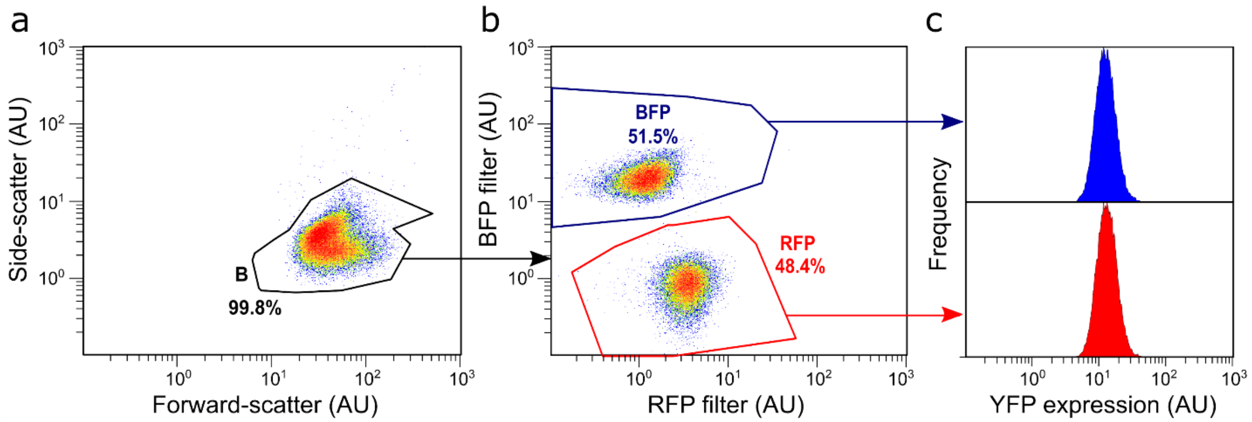

Supplementary Fig. 22. Raw flow cytometry data and gating procedure. Flow cytometry was used to distinguish between the YFP response of signal producing strains (expressing constitutive BFP) and signal receiving strains (expressing constitutive RFP) using the following gating scheme. a, cells were initially gated on their forward and side-scattering to exclude non-canonical elements. b, cells were then gated on their BFP and RFP levels to separate the two genotypes. c, finally, YFP expression histograms and medians were calculated for each of the populations and used to make the plot in Supplementary Fig. 15.

Supplementary Tables

Supplementary Table 1. Strain list.

Strain name	Genotype	Reference	Strain used in figure
AES2837	*Bacillus subtilis* PY79, wild type	BGSC‡	
DS2569	*Bacillus subtilis* 3610, plasmid cured	From Daniel Kearns lab	
AES2111	Δ*comQXP*::Tet	(Pollak et al., 2016)	
AES1334	*amyE*::(P_{srfA}-3xYFP-Spec)	(Bendori et al., 2015)	
AES5426	*lacA*::(P_{veg}-R0-mCherry-Mls)	pAEC1441 → AES2837	S21b
AES5427	*lacA*::(P_{veg}-R0-2xmTag-BFP -Mls)	pAEC1444 → AES2837	
AES6436	*lacA*::(P_{veg}-R0-mCherry-Mls); Δ*sacA*::Kan	BKK38040‡ → AES5426	4c, 4d, S21a
AES6045	*amyE*::($P_{hyperspank}$-*comP*-Spec); Δ*comQXP*::Tet; *yhdGH*::(P_{srfA}-3xYFP-Kan); *lacA*::(P_{veg}-R0-mCherry-Mls)	AES5426 → AES6039	ComX-Receiver 2B, 2E, S10
AES6149	*amyE*::($P_{hyperspank}$-*comP*-Spec); Δ*comQXP*::Tet; *yhdGH*::(P_{srfA}-3xYFP-Kan); *lacA*::(P_{veg}-R0-2xmTag-BFP-Mls); *sacA*::(P_{comQXP}-*comQX*-Cm)	AES2714 → AES6046	ComX-Full system 2B, 2E, S10
AES5923	*sacA*::(P_{srfA}-3xYFP-Cm); *amyE*::(P_{comQXP}-*rapP*^{T236N}-Spec); *lacA*::(P_{veg}-R0-mCherry-Mls)	AES5426 → AES5836	PhrP-Receiver 2D, 2E, S10, S11
AES6028	*sacA*::(P_{srfA}-3xYFP-Cm); *amyE*::(P_{comQXP}-*rapP*^{T236N}-Spec); *lacA*::(P_{veg}-R0-2xmTag-BFP-Mls); zjd-89::($P_{hyperspank}$-*phrP*-Kan,Cm,Spec)	AES4343 → AES5925	PhrP-Full system 2D, 2E, S10
AES5825	*sacA*::(P_{plcA}-3xYFP-Cm); *amyE*::(P_{43}-*plcR*$^{B. thuringiensis 407}$-Spec); *lacA*::(P_{veg}-R0-2xmTag-BFP-Mls)	AES5427 → AES5315	PapR-Receiver S11
AES5829	*sacA*::(P_{plcA}-3xYFP-Cm); *amyE*::(P_{43}-*plcR*$^{B. thuringiensis 407}$-Spec); *lacA*::(P_{veg}-R0-mCherry-Mls)	AES5426 → AES5315	PapR-Receiver 2c, 2e, S8, S10, S11, S12, S14, S15, S16, S22
AES5842	*sacA*::(P_{plcA}-3xYFP-Cm); *amyE*::(P_{43}-*plcR*$^{B. thuringiensis 407}$-Spec); *lacA*::(P_{veg}-R0-mCherry-Mls); Δ*scoC*::Tet	LFH-PCR → AES5829	PapR-Receiver Δ*scoC* 3a, 3b, S11, S12
JRL408	PB2::P_{spac}-*spo0K*	(LeDeaux and Grossman, 1995)	
AES6018	PB2::P_{spac}-*spo0K*; Δ*appA*::Tet	LFH-PCR → AES6018	
AES6055	*sacA*::(P_{plcA}-3xYFP-Cm); *amyE*::(P_{43}-*plcR*$^{B. thuringiensis 407}$-Spec); *lacA*::(P_{veg}-R0-mCherry-Mls); P_{spac}-*spo0K*; Δ*appA*::Tet	AES6018 → AES5829	PapR-Receiver P_{IPTG}-*opp* 3a, 3b, S12, S13
AES5935	*sacA*::(P_{plcA}-3xYFP-Cm); *amyE*::(P_{43}-*plcR*$^{B. thuringiensis 407}$-Spec); *lacA*::(P_{veg}-R0-mCherry-Mls); *yhdGH*::($P_{hyperspank}$-*papR*$^{B. thuringiensis 407}$-*lacI*-Kan)	pAEC1526 → AES5829	PapR-Full system S15, S22
AES5936	*sacA*::(P_{plcA}-3xYFP-Cm); *amyE*::(P_{43}-*plcR*$^{B. thuringiensis 407}$-Spec); *lacA*::(P_{veg}-R0-2xmTag-BFP-Mls); *yhdGH*::($P_{hyperspank}$-*papR*$^{B. thuringiensis 407}$-*lacI*-Kan)	pAEC1526 → AES5825	PapR-Full system 2c, 2e, 3a-f, S8, S10, S14, S15, S16, S22
AES6163	*lacA*::(P_{veg}-R0-2xmTag-BFP-Mls); *amyE*::(P_{comQXP}-*papR*$^{B. thuringiensis 407}$-Spec)	pAEC1652 → AES5427	PapR-constitutive producer 3a, 3b
AES7756	*lacA*::(P_{veg}-R0-2xmTag-BFP-Mls); *yhdGH*::($P_{hyperspank}$-*papR*$^{B. thuringiensis 407}$-Kan)	pAEC1932 → AES5427	PapR-constitutive strong producer S13
AES5919	*lacA*::(P_{veg}-R0-2xmTag-BFP-Mls); *amyE*::(P_{xis}-3xYFP-Spec)	pAEC1839 → AES5427	
AES6210	*lacA*::(P_{veg}-R0-2xmTag-BFP-Mls); *amyE*::(P_{xis}-3xYFP-Spec); Unmarked-ICEBs1; Δ*conB*::Kan	BKK04910‡ → AES6181	
AES6345	*lacA*::(P_{veg}-R0-2xmTag-BFP-Mls); *amyE*::(P_{xis}-3xYFP-Spec); Unmarked-ICEBs1; Δ*conB*::Kan; *sacA*::($P_{hyperspank}$-*rapI*-*phrI*-Cm)	pAEC1729 → AES6210	ICEBs1-Δ*conB* 4b-d, S19, S21
AES6604	*lacA*::(P_{veg}-R0-2xmTag-BFP-Mls); *sacA*::($P_{hyperspank}$-*rapI*-*phrI*-*lacI*-Cm); *amyE*:: (*immAR*-*xis*-3xYFP-Spec)	pAEC1754 → AES6568	

AES6608	*lacA*::(P_{veg}-R0-2xmTag-BFP-Mls); *sacA*::($P_{hyperspank}$-*rapI-lacI*-Cm); *amyE*:: (*immAR-xis*-3xYFP-Spec)	pAEC1754 → AES6570	PhrI-Receiver<br>4b, S19
AES6326	*yhdGH*::(P_{rapI}-*rapI-phrI*-Kan)	pJMJ354 → AES2837	
AES6728	*lacA*::(P_{veg}-R0-2xmTag-BFP-Mls); *sacA*::($P_{hyperspank}$-*rapI-phrI-lacI*-Cm); *amyE*::(*immAR-xis*-3xYFP-Spec); *yhdGH*::(P_{rapI}-*rapI-phrI*-Kan)	AES6326 → AES6604	RapI-PhrI regulatory system<br>S19, S21b
AES6528	*lacA*::(P_{veg}-R0-mCherry-Mls); *amyE*::(P_{aimR}-*aimR*-P_{aimX}-GFP-Spec)	pAEC1746 → AES5426	AimP-Receiver<br>4a, S19, S20
AES6456	*lacA*::(P_{veg}-R0-2xmTag-BFP-Mls); *amyE::*(P_{aimR}-*aimR-aimP*-P_{aimX}-*aimX*-GFP-Spec)	pAEC1745 → AES5427	AimP-Full system<br>4a, S19, S20

‡ Strain purchased form *Bacillus* Genetic Stock Center

Supplementary Table 2. Plasmid list.

Name	Description	Reference
pAEC277	pDL30::3xYFP-Spec (Amp)	Lab stock
pAEC310	ECE174::Cm (Amp)	BGSC‡
pAEC840	ECE174::P_{comQXP}-*comQX*-Cm (Amp)	(Bareia et al., 2018)
pAEC962	pDL30::P_{comQXP}-3xYFP-Spec (Amp)	Lab stock
pAEC1002	pDR111::$P_{hyperspank}$-*comP-lacI*-Spec (Amp)	(Bareia et al., 2018)
pAEC1003	ECE174::P_{srfA}3xYFP-Cm (Amp)	(Even-Tov et al., 2016)
pAEC1149§	pDL30::P_{plcA}-3xYFP-Spec (Amp)	Lab stock
pAEC1245	pDL30::P_{comQXP}-*rapP*^{T236N}-Spec (Amp)	(Bareia et al., 2018)
pAEC1272	pDR111::$P_{hyperspank}$-*phrP-lacI*-Spec (Amp)	(Bareia et al., 2018)
pAEC1413	ECE174::P_{plcA}-3xYFP-Cm (Amp)	This work
pAEC1441	pAEC1226::P_{veg}-R0-mCherry-Erm (Amp)	Lab stock
pAEC1444	pAEC1226::P_{veg}-R0-2xmTag-BFP-Erm (Amp)	Lab stock
pAEC1504	pMMH253::Kan (Amp)	From Alan Grossman
pAEC1526	pMMH253::$P_{hyperspank}$-*papR*$^{B.\ thuringiensis\ 407}$-*lacI*-Kan (Amp)	This work
pAEC1541	pMMH253::P_{srfA}-3xYFP-Kan (Amp)	This work
pAEC1652	pDL30::P_{comQXP}-*papR*$^{B.\ thuringiensis\ 407}$-Spec (Amp)	This work
pAEC1727	ECE174::$P_{hyperspank}$-*rapI-lacI*-Cm (Amp)	This work
pAEC1729	ECE174::$P_{hyperspank}$-*rapI-phrI-lacI*-Cm (Amp)	This work
pAEC1754	pDL30::*immAR-xis*-3xYFP-Spec (Amp)	This work
pJMJ354	pMMH253::P_{rapI}-*rapI-phrI*-Kan (Amp)	From Alan Grossman lab
pAEC1745	pDR111::P_{aimR}-*aimR-aimP*-P_{aimX}-*aimX*-GFP-Spec (from phi3T) (Amp)	From Rotem Sorek lab
pAEC1746	pDR111::P_{aimR}-*aimR*-P_{aimX}-GFP-Spec (from phi3T) (Amp)	
pAEC1839	pDL30::P_{xis}-3xYFP-Spec (Amp)	This study
pAEC1932	pMMH253::$P_{hyperspank}$-*papR*$^{B.\ thuringiensis\ 407}$-Kan (Amp)	This study
pAEC2148§	pDR111::$P_{hyperspank}$-*papR*$^{B.\ thuringiensis\ 407}$-*lacI*-Spec (Amp)	Lab stock
pAEC2161§	pDR111::P_{43}-*plcR*$^{B.\ thuringiensis\ 407}$-Spec (Amp)	Lab stock

§ Genomic DNA of *Bacillus thuringiensis* strain 407 is from Didier Lereclus Lab

Supplementary Table 3. Primer list.

Primer name		Sequence$	Used for
PTB161	AppA P1	TGCCCCAAGGTGCCCGAAA	*appA* deletion
PTB405	AppA del_tet P2	CCATGCCAAGTGTAATGCCCGCAGCGGCTGTTGTTTATGTAGTGTGC	*appA* deletion
PTB406	AppA del_tet P3	GGCAGTAGCGCGGTGGTCCCACCCCTTAAAGGGGAGGGAGAGC	*appA* deletion
PTB164	AppA P4	CGCGCTGGAATGCTGAATCG	*appA* deletion
PTB409	tet F	GCTGCGGGCATTACACTTGGCATGG	*appA* deletion
PTB422	tet F V2	ACACTTGGCATGGACGAACTT	*scoC* deletion
PTB410	tet R	GTGGGACCACCGCGCTACTGCC	*appA* & *scoC* deletion
PTB415	ScoC del P1	TGTGCTGAATACAATCTCAATGATCATTATTTATCCG	*scoC* deletion
PTB421	ScoC del P2	AAGTTCGTCCATGCCAAGTGTCATAGGGCGGTTCCACTCGATTC	*scoC* deletion
PTB420	ScoC del P3	GGCAGTAGCGCGGTGGTCCCACGAAGAGCTCGAACCTGTAAACAG	*scoC* deletion
PTB416	ScoC del P4	AGCCAGCAGGAAATCAGGC	*scoC* deletion
PTB332	TM2505 sacP F	CTGATTGGCATGGCGATTGC	Verify integration into *sacA*
PTB335	TM2508 ywdA R	TCCAAACATTCCGGTGTTATC	
PTB445	YhdGH loc F	AGAATAATGACCATTGTTACTCATGAAT	Verify integration into *yhdGH*
PTB446	YhdGH loc R	GGCATGGGCACCGTTGATATCATC	
PTB317	AmyE up F	CGGCGCTTTGAAGCTTGG	Verify integration into *amyE*
PTB318	AmyE down R	CCAAACGGATCATACAACTGCTCC	
PTB328	LacA loc Rev	GAACATTTTCACTTTGCTTTTCA	Verify integration into *lacA*
PTB329	LacA loc For	ATACCGGTTGCCGTCATCTTT	
PTB330	LacI KpnI R	AGT**GGTACC**CCTAACTCACATTAATTGCG	pAEC1526
PTB373	Phs Up EcorI	AGCT**GAATTC**ATTCACGAACGAAAATCG	pAEC1526
PTB449	pMMH253 KpnI F	TGAAT**GGTACC**GTCTAGAACCATTTGAGGTGATAGG	pAEC1541 and pAEC1932
PTB450	pMMH253 EcorI R	GCTTAT**GAATTC**GGCCATACAG	pAEC1541
PTB361	PsrfA EcorI F	ATGGG**GAATTC**CGTTGTAAGACGCTC	pAEC1541
PTB453	YFP KpnI R	ATTA**GGTACC**CGGACTCCTCAAACTATATGCG	pAEC1541
PTB303	pDRIII R	CGGAAGGAAATGATGACCTCGTTTCCACC	pAEC1652 and pAEC1932
PTB469	PapR SpeI F	ATGCT**ACTAGT**GCTTAAAGGTGGTGAAGATATCATG	pAEC1652
PTB320	pDL30 SphI R	ATTGC**GCATGC**CATATGATACCGTCGGGCGG	pAEC1652
PTB209	PQXP NheI R	CTTA**GCTAGC**CTCCTTGATCCGGACAGAATC	pAEC1652
PTB526	Pxis-MfeI-F	GGC**CAATTG**TCTCTGCGGCTTCTTTCTGAG	pAEC1839
PTB527	Pxis-BamHI-R	GGC**GGATCC**CACCTCCTCGTTAACTCAACT	pAEC1839
PTB474	RapI SacI F	ATGC**GAGCTC**TTCGCACAATTTTATGTAAGGATGG	pAEC1727 and pAEC1729
PTB475	RapI NheI R	TCGA**GCTAGC**AATACACTACTTAAAATCACTGCTGC	pAEC1727
PTB476	PhrI NheI R	ATCA**GCTAGC**CCAATTATCTAAGCTATGCCCCC	pAEC1729
PTB503	ImmA MfeI P1	ATGCA**CAATTG**CAAATCCAAAGCTAACGTCAACTTG	pAEC1754
PTB504	xis BamHI P2	AGCA**GGATCC**AAAATCACCCCGTTTGAGAT	pAEC1754

$ Nucleotides recognized by restriction enzymes are marked in bold

Supplementary Discussion

1 Quorum-sensing designs

We use this section of the supplementary information (1) to discuss the features of the absorbing and non-absorbing quorum-sensing designs in more detail, (2) to discuss the regulatory details of the quorum-sensing systems we examined in the main text, and (3) to discuss alternative mechanisms, besides those discussed in the main text, that could shorten the communication range.

1.1 Absorbing and non-absorbing quorum sensing systems

As described in the main text, we discriminate between two types of quorum-sensing designs, based on how signal molecules are being processed: non-absorbing quorum-sensing systems allow for continued signal propagation after sensing, while absorbing systems couple signal sensing to irreversible signal uptake, thereby limiting their diffusion. Each quorum-sensing design could further be categorized into two subtypes (Supplementary Fig. 4). Non-absorbing systems can be categorized based on the location where the signal molecules are sensed: (A) Extracellular signaling and (B) intracellular signaling with reversible signal uptake. Absorbing systems can be categorized based on the nature of the signal molecules: (A) Peptide-based uptake systems and (B) non-peptide-based uptake systems. In the following paragraphs, we briefly discuss each subtype.

1.1.1 Non-absorbing design. Type A: Extracellular signaling

Non-absorbing quorum-sensing designs with extracellular sensing are common among the Gram-positive bacteria, with three particularly well-studied systems (Supplementary Fig. 4a): ComQXP in *Bacillus subtilis*[1,2], ComABCD in *Streptococci*[3] and Agr in *Staphylococci* and other Gram-positive bacteria[4–6]. Extracellular signaling is also found in multiple *Vibrio* species[7,8]. In all cases, the receptor is membranal with an extracellular signal-binding domain. Binding of the signal to the receptor changes its kinase activity (either turning it on in Gram-positive or off in Vibrios), leading to changes in response-regulator activity and further downstream changes in gene expression. In Gram-positive bacteria, the signal molecules are modified or long peptides exported through a specific exporter[9]. The signals and receptors in Gram-positive bacteria show high intraspecific

variability, where different strains of the same species code for different signal-receptor variants that show no cross-communication[10]. Therefore, even within a co-culture of strains of the same species, one can expect to find multiple signal-receptor pairs. In Gram-negative bacteria the signals are small molecules and diversity is interspecific[11].

Unlike eukaryotic membranal receptor, there is no known direct effect of receptor binding on signal uptake or degradation. More generally, the signaling molecules of these systems are not known to be imported by cells. Gram-positive peptide signals can be extracellularly degraded, but this is most likely reduced in the Agr and ComQXP systems by the post-translational modification of the peptide.

1.1.2 Non-absorbing design. Type B: Intracellular signaling with reversible signal uptake

Intracellular sensing with reversible signal uptake is observed in the acyl homoserine lactone (AHL) LuxIR-like systems, which are prevalent among the Gram-negatives (Fig. 2b). The signal molecules in these systems are AHLs with varying tail acyl chain length and side-chain modifications[11,12]. The signal molecules are produced intracellularly by a luxI-type synthase and are subsequently exported. The LuxR-type receptor that senses the signal is intracellular, signal molecules therefore need to be imported before sensing. Signal uptake is reversible. Thus, signal molecules can be taken up and secreted multiple times. In principle, the effective signal diffusion could be reduced by this repeated import and export of signal molecules. Yet, in general, AHL producing bacteria do not show strong degradation of the signal they make, and we therefore expect type B systems to have long-range communication. For potential exceptions to this rule, see the discussion below.

1.1.3 Absorbing design. Type A: Peptide-based irreversible uptake

The largest class of absorbing quorum-sensing systems is that of the RNPP superfamily, which relies on small signal peptides for communication (Fig. 2c). The RNPP superfamily is highly abundant in the Gram-positive bacteria[13,14]. It is based on a cytoplasmic receptor which typically functions as a transcription factor. Some important exceptions occur, including the Rap-Phr systems discussed in the main text (RapP-PhrP and RapI-PhrI), where Rap proteins act as stoichiometric inhibitors or as phosphatases[15,16]. In all RNPP systems, the signal molecule is translated as a pre-pro-signal and is cleaved for the first time during secretion and a second time

extracellularly by secreted or membrane-bound proteases[17,18]. The resulting mature signal peptide has a length between 5 to 10 amino acids. The mature peptide can be imported back into the cell through the general oligopeptide permeases[19]. In a similar manner to the Gram-positive type A non-absorbing systems, these systems show very high intraspecific variability, where different strains encode for different variants of the signal-receptor pair, which show no or minimal interference[20]. Therefore, even when co-culturing strains from the same species, one expects to find different signal-receptor pairs. Quorum-sensing systems from the RNPP superfamily are also prevalent among mobile genetic elements, as described in the main text.

Since practically all Gram-positive bacteria and many Gram-negative bacteria express the oligopeptide permeases needed to import the short signal peptides[21], we expect that nearly all cells in the community, including cells lacking the quorum-sensing system, will import the signal molecules. This implies that type A absorbing quorum-sensing design is expected to express the characteristic short-range communication as discussed in the main text.

1.1.4 Absorbing design. Type B: Non-peptide-based irreversible uptake

The final subtype of quorum-sensing designs is that of the non-peptide absorbing systems (Fig. 2c). The AI-2 system in *Escherichia coli* and related species belongs to this category of quorum-sensing systems. These systems utilize the AI-2 signal molecule, which is derived from the excreted metabolite 4,5dihydroxy-2,3-pentanedione (DPD) that is made by the LuxS enzyme in the activated-methyl-cycle pathway[22]. The reception system acts like a sugar import system (e.g the Lac system): the AI-2 molecule is first imported through a specific permease and is then phosphorylated (preventing export) and subsequently degraded[23]. The phosphorylated form is sensed by the LsrK receptor which further activates the expression of the permease and associated genes as well as other bacterial traits[24,25]. Although many bacteria secrete DPD, not all bacteria have the permease needed for importing AI-2[26]. This implies that the AI-2 quorum-sensing system is expected to behave like a short-range communication system within signal-producing clusters, which all express the permease, but could simultaneously act like a long-range communication system across signal-producing clusters, because interspersing cells do not (always) import the signal. Interestingly, AI-2 is also sensed by marine *Vibrio* through a type A non-absorbing quorum-

sensing system[27]. There is no evidence for signal uptake in this system and is therefore expected to strictly function as a long-range communication system.

1.2 Absorbing and non-absorbing quorum-sensing systems discussed in the main text

In this section, we provide a detailed description of the regulatory pathways underlying the five quorum-sensing systems discussed in the main text (Supplementary Fig. 9 and 18):

1. **ComQXP system** (Supplementary Fig. 9a). The ComQXP (or ComQXPA) quorum-sensing system contains three specific constitutively expressed genes in a single operon: *comQ*, *comX* and *comP*. The pro-peptide of the ComX signal is encoded by the *comX* gene. It is cleaved and prenylated on a conserved tryptophan by the ComQ enzyme[1,2]. The modified peptide is then secreted through an unknown mechanism. ComP is a seven-transmembrane receptor kinase whose ComX binding domain is located extracellularly[28]. Upon ComX binding the receptor is phosphorylated and subsequently transfer the phosphoryl group to the ComA response regulator. ComA is also a target for repression by multiple Rap receptors, including RapP, which will be discussed next[29,30]. ComA directly regulates tens of operons, including the well-studied *srfA* operon which governs production of the surfactant surfactin[29] and indirectly the induction of competence[31]. The ComQXP system is found in all *B. subtilis* group strains and more sporadically in other Firmicutes[32,33]. There are many pherotypes of this system, with different signaling peptide length (6aa-10aa) and different modifications, always done on a tryptophan residue[9].
2. **PlcR-PapR system** (Supplementary Fig. 9b). This system is found in many *B. cereus* group isolates and is a part of the RNPP superfamily[34]. PlcR is a transcription factor that dimerizes upon signal binding and binds to a variety of promoters, including the *plcA* promoter used in this work. In *B. thuringiensis*, PlcR controls the virulence of the bacterium upon insect infection[35]. PapR is secreted through a similar pathway to that of Phr (see below), but the final peptide is 7aa long in its original host[36]. There are multiple PlcR-PapR pherotypes and the one used in this work codes for the signaling peptide ADLPFEF[36].
3. **RapP-PhrP system** (Supplementary Fig. 9c). This system is part of the Rap-Phr family of quorum-sensing systems, which is one branch of the RNPP superfamily[37]. The RapP-PhrP system is found on the pLS32 plasmid of strain NCBI3610[38,39]. The RapP receptor of this

system is mutated in a key conserved residue, which makes it insensitive to the PhrP signaling peptide and, hence, constitutively active. For this study, we made use of a RapP receptor in which the mutated residue is converted to the consensus amino-acid, which again makes it responsive to the signal molecule[38]. The RapP-PhrP system is encoded by the *rapP-phrP* operon, which is regulated by ComA. *phrP* has an internal promoter regulated by Spo0A. Interestingly, the intracellular receptor of this system, RapP, has been shown to repress both ComA and the Spo0A pathway (probably through dephosphorylation of Spo0F)[38]. Like for other Phrs, PhrP is expressed as a pre-pro-peptide encoding for a secretion signal sequence which is cleaved during secretion and a pro-domain which is cleaved extracellularly by several secreted/membrane-bound proteases[13,39]. The final product (ADRAAT) is imported into the cells through the Oligopeptide permease system (Opp), which is used for general import of peptides[40]. A second permease system capable of importing peptides, the App system, is mutated in the lab strain used in this work[41]. The Opp system is negatively regulated by the ScoC transcription factor, whose deletion is used in this work to increase the signal uptake rate (Fig. 3). It is worth noting that ScoC also regulates the extracellular proteases which govern Phr cleavage[42]. More generally, there are multiple Rap-Phr systems in the *B. subtilis* and *B. cereus* group, coding for orthogonal peptides[20]. The lab strain of *B. subtilis* has eight full Rap-Phr systems and three orphan Rap receptors. Most of these regulate ComA and Spo0F, but there are some exceptions (see RapI-PhrI system below).

4. **Arbitrium (AimR-AimP) system** (Supplementary Fig. 18a). This recently identified family is also part of the RNPP superfamily[43]. It is encoded by many *B. subtilis* and *B. cereus* phages and the AimR receptor was shown to regulate lysis-lysogeny decision[43,44]. In the ϕ3T phage, where this system is most intensely studied, AimR and AimP are expressed immediately after infection. AimR positively regulates the expression of the *aimX* gene, which activates the lytic pathway. AimP secretion, maturation and import are similar to that of other signal molecules in the RNPP superfamily (see above). AimP binding to AimR block its activity and therefore prefer the lysogenic pathway. Thus, at high infection levels, the system promotes lysogeny, while during the initial phase of infection it promotes lysis[43].

5. **RapI-PhrI system** (Supplementary Fig. 18b). This system is encoded by the ICE*Bs1* integrative and conjugative element[45]. Its regulation is similar to that of RapP-PhrP and so is the PhrI maturation pathway. In contrast to most Raps, RapI control the induction of its conjugative element. This is done through the ImmA-ImmR signal transduction pathway[45]. RapI activates ImmA catalytic activity. ImmA in turn cleaves and inactivates the ICE*Bs1* repressor, ImmR[46]. It is worth noting that ImmA is also activated by DNA damage through RecA activation[45]. We hypothesize that the few cells that induce conjugation in large clusters of signal-producing cells might actually trigger conjugation due to DNA damage, since the concentration of quorum-sensing signal in these clusters should be high (hence, suppressing conjugation).

1.3 The relation between the communication range and other spatial measures

For the purpose of our study, we operationally define the communication range as the distance over which the signal response is reduced by one order of magnitude from the boundary between signal producers and receivers, irrespective whether the response is saturated or not near the boundary (Supplementary Fig. 1). In the simplest scenario, where the absorbing systems can be described by a linear model, the communication range is identical to both the signal decay length-scale and response decay length-scale, which we detail below. In section 1.3.1, we discuss this simple linear scenario, using a one-dimensional mathematical model. We show how the diffusion rate, uptake rate and cell density affect the communication range. In addition, we discuss how the communication range relates to the detection range, which is the maximum distance over which the signal molecules can be detected from the boundary of signal producers. In section 1.3.2 we discuss how deviations from linearity affect the communication range and the other length-scale measures.

1.3.1 Linear scenario

In this section, we consider a simple one-dimensional model of signal propagation, where signal receiving cells respond to signal produced by a cluster of signal producing cells. A more general derivation of how signal concentrations are distributed in and outside of a cluster of signal producers is given in section 2 of the Supplementary Discussion (this elaborate analysis also includes a two and three dimensional evaluation of signal propagation).

The simple model assumes that signal can diffuse with a diffusion constant D and is uptaken by cells with a degradation level which is linearly proportional to the uptake rate per cell α_c and the cell density d. We assume a constant flux (η) boundary condition at $x = 0$, and an open boundary condition to the right (i.e concentration of zero at infinity). We further assume that the output response we observe is linearly proportional to the signal level (scaling with m). Defining c as the signal concentration and Y as the output. We find the following equations for the system:

[1] $\frac{\partial c}{\partial t} = D\frac{\partial^2 c}{\partial x^2} - \alpha_c dc$

[2] $D\frac{\partial c}{\partial x}\Big|_{x=0} = -\eta$

[3] $Y = f(c); f(c) = mc$

Here, we assume linearity in both signal uptake ($\alpha_c dc$) and response ($f(c) = mc$). The solution to these equations is given by:

[4] $c = c_0 \exp\left(-\frac{x}{\lambda}\right); \lambda = \sqrt{\frac{D}{\alpha_c n}}; c_0 = \frac{\eta\lambda}{D}$

[5] $Y = mc$

We can now mathematically define various range measures (Supplementary Fig. 1):

- **Communication range ($\boldsymbol{\lambda}$)**: As mentioned above, we define the communication range as the distance over which the signal response is reduced by one log scale from its value at the boundary, irrespective whether the response is saturated or not near the boundary (see a discussion below on saturation). In a linear model, the communication range is equal to both the signal decay length-scale (λ_{signal}) and response decay length-scale ($\lambda_{response}$).
- **Signal decay length-scale ($\boldsymbol{\lambda}_{signal}$)**: The signal decay length-scale is defined by the distance over which the signal concentration decays one order of magnitude (on a natural log scale). One can more generally define the local signal-decay length-scale as:

  [6] $\lambda_{signal}(x) = \frac{dx}{dlog(c)} = c\frac{dx}{dc}$

  This length-scale can be determine using the distance over which the signal concentration decays from the boundary of signal producers (Supplementary Fig. 1a).

- **Response decay length-scale ($\boldsymbol{\lambda_{response}}$)**: Similarly to the signal decay length-scale, we define the response decay length-scale as the distance over which the response reduces one natural log. We measure this length-scale in the region where the signal response is undergoing an exponential decline (so not including the region of saturated signal response). Mathematically this is described by the following equation:

  $$[7]\ \lambda_{response}(x) = \frac{dx}{dlog(Y)} = Y\frac{dx}{dY} = \frac{Y}{c}\frac{dc}{dY} \times c\frac{dx}{dc} = \left(\frac{dlog(Y)}{dlog(c)}\right)^{-1} \times \lambda_{signal}(x)$$

  We therefore find that the local response decay range is proportional to the local signal decay range at the point divided by the logarithmic sensitivity of the response to the concentration, $\frac{dlog(Y)}{dlog(c)}$. For a linear response function this is equal to $\mathbf{1}$ and therefore the two range measures are equal.

- **Detection range ($\boldsymbol{\lambda_{detection}}$):** Operationally, this is defined as the distance between the boundary and the point where response level is at the background level of expression. If the background level is Y_{bg}, then this distance is calculated from eqs. [4]-[5] as:

  $$[8]\ \lambda_{detection} = \lambda \times log\left(\frac{m \cdot c_0}{Y_{bg}}\right)$$

  The maximal response distance therefore depends logarithmically on the production rate, the threshold response and the transfer function of signal to response.

1.3.2 Non-linear scenario

The above analysis assumes that both the uptake rate and response are linear with the local signal concentration. Both of these assumptions can be violated either towards a saturated regime (sub-linear relation) or a hyper-sensitive regime (super-linear relation). We consider separately the impact of non-linearity in either signal uptake or response. The table below summarizes the main results of the analysis, where λ_x^{linear} functions as a reference of the spatial measure under the linear regime.

Scenario	Type	λ	λ_{signal}	$\lambda_{response}$	$\lambda_{detection}$
Uptake	Saturated	No steady state	No steady state	No steady state	No steady state
	Hyper-sensitive	$\lambda < \lambda^{linear}$	$\lambda_{signal} < \lambda_{signal}^{linear}$	$\lambda_{response} = \lambda_{signal}$	$\lambda_{detection} < \lambda_{detection}^{linear}$
Response	Saturated	$\lambda > \lambda^{linear}$	$\lambda_{signal} = \lambda_{signal}^{linear}$	$\lambda_{response} = \lambda_{response}^{linear}$	$\lambda_{detection} > \lambda_{detection}^{linear}$
	Hyper-sensitive	$\lambda = \lambda^{linear}$	$\lambda_{signal} = \lambda_{signal}^{linear}$	$\lambda_{response} = \frac{\lambda_{response}^{linear}}{n}$	$\lambda_{detection} < \lambda_{detection}^{linear}$

1.3.2.1 *Non-linearity in signal uptake*

The uptake rate per cell can change non-linearly with the signal concentration, for example, because the uptake machinery is saturated by signal, leading to a sub-linear uptake response. For a simple model of signal uptake, we expect a Michaelis–Menten uptake per cell dependence of $\alpha_c = \frac{\alpha_c^0}{1+c/K}$. In contrast, if the signal positively influences the uptake rate, we may also find a situation where the signal decay is super-linear, $\alpha_c = \alpha_c^0 \left(\frac{c}{K}\right)^n$. We consider the impact of each of these scenarios on the different spatial measures (Supplementary Fig. 2):

- **Saturated uptake**. If the uptake is saturated near the boundary, then we expect that the signal will decay more slowly than it would have in the non-saturated case. For a one dimensional scenario, the modified diffusion equation has no steady state if production rate is sufficiently high to saturate signal uptake near the boundary. The signal concentration near the boundary will grow logarithmically and the signal decay length-scale thereby increases as well with time. As signal concentration increases, the signal profile would approach that of a non-absorbing system. The time-scale at which this happens depends on the relative production rate and the geometry of the system, making it unlikely for this to happen in the experimental setup we considered.
- **Hyper-sensitive uptake**. Previous works in the context of morphogen gradients have analyzed this case[47]. It was shown that this type of regulation of uptake rate leads to a steady state. The maximal response distance in this case is more robust to variation in production rate. The signal decay range is short near the production region and extends

at increasing distances from the boundary. This scenario is irrelevant to absorbing systems of the RRNPP type and therefore we do not consider it further. Note that it may be relevant to the AI-2 system (see section 1.1.4).

1.3.2.2 *Non-linearity in signal response*

Non-linearity in the response does not affect directly signal propagation, therefore we expect that the signal concentration profile will remain the same as in the fully linear case, eqs. [1]-[5]. We therefore do not expect the signal decay length-scale to change. In contrast, all response-related measures would change. The response decay length-scale would change by a factor inversely proportional to the sensitivity of the response to the local concentration, $\frac{dlog(Y)}{dlog(c)}$. We can consider for example, a modified hill coefficient response curve:

$$[9]\ Y = f(c) = Y_{min} + Y_{max} \frac{c^n}{c^n + K_c^n}$$

Y_{min}, Y_{max} are the minimal and maximal corresponding values of the response, K_c is the half max change signal concentration and n is the hill coefficient of the response. For $c \ll K_c$ we find a saturated minimal response, Y_{min}, while for $c \gg K_c$ we find a saturated maximal response Y_{max}. The sensitivity of the response function is:

$$[10] \qquad \frac{dlog(Y)}{dlog(c)} = n\left(1 - \frac{Y_{min}}{Y}\right)\left(1 - \frac{(Y - Y_{min})}{Y_{max}}\right)$$

For intermediate values of the response the sensitivity approaches the hill coefficient n, while for saturating values (at either low or high signal concentration), we find that the sensitivity is approaching zero. In accordance with that and using the definitions of the various spatial measures, we find that the response decay length-scale in the region of exponential decline (i.e, $Y_{min} \ll Y \ll Y_{max}$) is equal to:

$$[11] \qquad \lambda_{response} = \frac{\lambda_{signal}}{n}.$$

Saturation would therefore affect the communication range under two different conditions (Supplementary Fig. 3):

- **High signal concentration at the boundary**: If the concentration at the boundary is high, $c_0 \gg K$, then we would first have a spatial region, whose length is $\sim\lambda \log\left(\frac{c_0}{K}\right)$, where cells show a saturated signal response. This will be followed by a region with exponential decay of the signal response with a response decay length-scale of $\frac{\lambda_{signal}}{n}$ which would end as the response approaches the minimal level.
- **Low signal concentration at the boundary**: If concentration at the boundary is very low $c_0 \ll k,\ Y \sim Y_{min} + \Delta;\ \Delta \ll Y_{min}$, then the response decay range would be $n\left(1 - \frac{Y_{min}}{Y_{min}+\Delta}\right)\left(1 - \frac{\Delta}{Y_{max}}\right) \sim n\frac{\Delta}{Y_{min}} \ll n$. That is, the response is saturated already near the boundary and the communication range is longer than λ. This explains the phenomenon of the extended communication range we observe at low expression levels of PapR (Supplementary Fig. 14).

1.4 Empirical considerations

1.4.1 Signal concentration is not expected to saturate *opp* uptake system

A recent study from Babel and colleagues[48] examined the temporal dynamics of signal response in the RapA-PhrA absorbing quorum-sensing system, where they quantified the RapA response to external PhrA levels using accurate FRET-based method. This work estimated that, in early stationary stage, the PhrA concentration is $\sim 1nM$. This concentration is the product of both signal production and signal uptake and is therefore expected to be largely independent of the cell density (which is much higher in our microfluidic chambers). They estimated that the PhrA concentration is about 100-fold lower than the affinity of the Opp uptake system. Although it is unclear if PhrA production and uptake affinity are equivalent to those of PapR, given the 100-fold margin between signal concentrations and uptake affinity, we believe it is reasonable to assume that saturation is not reached for the PlcR-PapR system either. This is furthermore supported by the fact we observe relatively short communication ranges. When signal uptake would be strongly saturated, absorbing systems are expected to behave more like non-absorbing systems, drastically increasing the communication range that is observed (see mathematical derivation in section 1.3.2).

1.4.2 Predicted communication range based on previous parameter estimates

As described below, the exponential decay length-scale, which equals the communication range in linear systems, is equal to $\lambda = \sqrt{D/\alpha}$, where D is the diffusion rate of the signal molecule and α is the effective uptake rate (molecules per second). In order to estimate this length-scale, we therefore need to estimate the diffusion and uptake rates. Using stokes equation and the estimated radius of a short peptide (0.5kDa→0.5nm radius), we estimate that signal peptides diffuse in water at a rate in the order of $D_{peptide} \sim 400\ \mu m^2 second^{-1}$. To measure the uptake rate, we need first to estimate the uptake rate per cell, which will of course depend on the level of expression of the Opp system and the specific peptide. Two papers have measured Opp uptake rate in *B. subtilis*. Lazzazera and colleagues[30] used radioactive labeling and estimated the uptake rate of CSF (PhrC) peptide to be 30 molecules per second per cell at an external concentration of $10nM \sim 6 \times 10^{12} \frac{molecules}{ml}$. The uptake rate per cell is therefore $\alpha_c = 5 \times 10^{-12} \frac{1}{\frac{cells}{ml} \times seconds}$. At a cell density of $d = 10^8 \frac{cells}{ml}$ this leads to a timescale of: $\tau_d = (\alpha_c d)^{-1} = 2000\ seconds \sim 40\ minutes.$ A more recent measurement of PhrA uptake rate using a FRET-based live reporter found numbers that are a factor of ~ 7 higher[48]. The uptake rate within the microfluidic chamber is calculated using the density of the cells in the chamber, n_c, and the uptake rate per cell, α_c. For simplicity, we could say that the volume fraction of cells in the chamber is roughly 0.5 (Supplementary Fig. 7), which implies a cell density of $d \approx 2 \times 10^{11} \frac{cells}{ml}$. Using the above parameter estimates, we find that $\alpha = \alpha_c d \sim 0.1 - 1\ second^{-1}$. Altogether, this implies a length-scale $\lambda = \sqrt{\frac{D}{\alpha}} \sim 6\mu m - 20\mu m$. This value fits very well with the length-scales observed in our work.

1.4.3 Signal-response curves of absorbing quorum-sensing systems

1. **PlcR-PapR**: The response of this system is linear for a wide range of signal concentrations (i.e. hill-coefficient = ~1; Supplementary Fig. 11). Saturation only occurs at high signal concentrations, which do not seem to occur in the microfluidic experiment, since the signal response already decays inside the cluster of signal-producing cells (in contrast to for example the ComQXP system, where signal receivers close to the producers show a saturated signal

response; Supplementary Fig. 10c). Altogether, this implies that the observed communication range is about equal to the signal decay length-scale (Supplementary Fig. 1, Scenario 1).

2. **RapP-PhrP:** This system has a non-linear response with a hill-coefficient of ~2-2.5 (Supplementary Fig. 11). Signal response profiles show a saturated signal response at the boundary of signal producers and sometimes even beyond the boundary (Supplementary Fig. 10c and Fig. 2d), suggesting high levels of PhrP production. In these cases, our measured communication range is a sum of two terms (see Supplementary Fig. 1, Scenario 2):

a. The saturation range: a distance over which the response is saturated.

b. The response decay length-scale: a distance over which the response decays by a factor of e from the point in space where the response becomes non-saturated. The response decay length-scale is equal to the signal decay length-scale divided by the hill coefficient of the non-linear response (Supplementary Fig. 1).The response decay length-scale is $\lambda_{response} = 3\mu m \pm 0.3\mu m$ ($n = 16$). We note that according to equation [11], the signal decay length-scale is a factor of 2-3 longer than the response decay range, because of the hill coefficient of the response.

3. **ICE*Bs1* and phage $\mathbf{\Phi}$3T systems**: In both systems, the response function is repressive (a negative hill coefficient). In addition to the communication range, we also define the zone of inhibition for these quorum-sensing systems (see Methods 'zone of inhibition and conjugation in the ICEBs1 system'), which is the distance from the boundary of signal producers at which the repression becomes ineffective. Supplementary Fig. 19 shows both the response decay length-scale (ICE*Bs1*: $1.98\mu m \pm 0.1\mu m$, mean ± s.e., $n = 14$; phage Φ3T: $1.6\mu m \pm 0.1\mu m$, $n = 45$) and the zone of inhibition (ICE*Bs1*: $4.2\mu m \pm 0.2\mu m$, $n = 14$; phage Φ3T: $6.6\mu m \pm 0.2\mu m$, $n = 45$). We have not measured the response hill coefficient for these systems, so we cannot deduce the signal decay length-scale.

1.4.4 Peptide length, signal uptake and communication range

We note that the communication range of the PlcR-PapR system is far longer than that of the other absorbing quorum-sensing systems we examined (i.e. RapP-PrhP, RapI-PrhI and AimR-AimP systems). In addition to the difference in the hill coefficient (Supplementary Fig. 11), there may be other biological differences that could explain the different communication ranges. The PlcR-PapR

system is exogenous and utilizes a 7-mer peptide as a signal (Bouillaut et al. 2008), while the other absorbing quorum-sensing systems are endogenous to *B. subtilis* and utilize a 5-mer or 6-mer system[38,43,45]. The lab strain of *B. subtilis* (PY79) that we used in this study only has a single oligopeptide permease system (Opp), which has a limited ability to import longer peptides[40]. The 7-mer peptide of the PlcR-PapR system might therefore be imported at a lower rate than the signal molecules of the other absorbing systems we tested, explaining slightly longer communication range of the PlcR-PapR system (still being much shorter than the communication range of the non-absorbing I ComQXP system; Fig. 2). It is however worth noting that wild strains of *B. subtilis* code for a second oligopeptide permease system (App, mutated in the lab strain[41]) which is suitable for importing longer peptides[49]. Wildtype *B. subtilis* strains as well as the natural host of the PlcR-PapR system (*B. thuringiensis*) might therefore have higher uptake rates of this peptide and express shorter communication ranges than the ones measured in this study (Fig. 2).

1.4.5 Reasons for the extended communication range of small clusters

As we noted in the main manuscript and show in Supplementary Figs. 14, 15, and 16, very small clusters of signal producers from the PlrR-PapR quorum-sensing system (width of ~1 cell) show a distinct signal response profile than larger clusters: (1) There is no continuity of expression between the producers and receivers in their signal response (Supplementary Figs. 15 and 16) and (2) the measured communication ranges associated with very small clusters are far longer than those of larger clusters (Supplementary Fig. 14). These two major differences can be explained by (a combination of) the following mechanisms:

1. Cells in small clusters produce few signal molecules and therefore approach minimal response levels, i.e. minimal reporter gene expression (associated with leakiness of the promoter). Consequently, the response curve becomes saturated in the lower range (i.e. low signal concentrations). As discussed above, this saturation results in larger estimates of the response decay length scale and, hence, larger communication ranges.
2. As apparent in liquid cultures (Supplementary Fig. 15), the PlcR-PapR system shows a self-sensing-like behavior [50], where signal-producing cells show a slightly higher intrinsic signal response (i.e. reporter gene expression) than signal-receiving cells. This form of self-sensing could explain the gap in signal response between producers and receivers that is

apparent with small clusters of signal producers (Supplementary Fig. 16). Self-sensing may also slightly skew the estimated communication range.

3. Temporal changes in the cluster size could also lead to a gap in signal response between producers and receivers. For example, a small cluster of signal producers may have been considerably larger a few cell cycles ago. This could result in temporarily elevated signal response in the cluster of signal producers compared to the surrounding receivers.
4. Finally, low signal-to-noise ratios could affect the estimated communication range. The signal response in small clusters of signal-producers is necessarily lower than those in larger clusters, because the absolute production of signal molecules is lower. As a consequence, there is a smaller dynamical range in signal response, which makes it harder to accurately infer the communication range.

1.5 Alternative mechanisms that could limit signal diffusion in space

In the main text, we focus on irreversible signal uptake as a mechanism that limits signal diffusion and hence the communication range between cells. Yet, there are alternative mechanisms that could limit diffusion. For example, there are a few cases where signal molecules are actively degraded by extracellular (type A and B non-absorbing designs) or intracellular enzymes (type B non-absorbing design)[51–53]. Although degradation is generally predicted to be weak, when degradation is sufficiently strong it could lead to limited diffusion.

For example, the fairly stable AHL molecules in type B non-absorbing quorum-sensing systems[54] can be degraded by lactonases[55] or acylases[53]. While most AHL-producing bacteria only weakly degrade AHL, there is one notable exception: *Pseudomonas putida* IsoF[56,57]. Here, the extracellular lactonases that degrade the native AHL (3-oxo-decanoyl AHL) are likely induced by quorum-sensing and lead to temporal pulse in quorum-sensing activity, where one first observes an increase in activity followed by a decrease. At relatively low cell densities (~10^9 cells/ml), the signal was shown to degrade within $\sim 1\ hour$[56,57]. As degradation is density-dependent, we expect that the rate of degradation increases a hundred-fold or more when densities approach those found in biofilms (~10^{11} cells/ml). We therefore hypothesize that the degradation rate can be sufficiently high to limit diffusion and hence result in short-range communication. Since lactonase enzymes

like signal molecules are secreted in the environment, degradation may lead to intricate spatial dynamics. Even when the lactonase enzymes are not secreted, complicated expression dynamics can emerge when quorum-sensing systems control the expression of lactonases. For example, in a synthetic quorum-sensing system in *Escherichia coli*, an intracellular lactonase was introduced under the control of a quorum-sensing system (similar to the *P. putida* case)[58]. This resulted in spatially coupled oscillations in gene expression, with a communication range of tens of microns.

Several well-studied lactonases are produced by bacteria that do not utilize AHL-based quorum-sensing[55]. The presence of such bacteria in a community with AHL producers is expected to reduce the concentration of AHL molecules in the community and could interfere with quorum-sensing (a process known as quorum quenching[55]). Yet, we do not expect that it would generally lead to short-range communication, because for this the producers should assort with the degrading cells in space, which we consider unlikely to occur.

Another option worth considering is the presence of signals whose spontaneous degradation (or modification into an inactive form) is very fast, independently of the presence of cells. In order to contribute to short range signaling, though, degradation needs to occur on a scale of seconds. There have been no attempts yet to consider the inter-cellular signaling role of such short-lived molecules, as their impact would be hard to trace in well-mixed environments.

1.6 The social context of absorbing quorum-sensing systems

The lack of crosstalk between clusters of signal producers with absorbing quorum-sensing systems relies on the fact that other cells in the community, which do not produce signal molecules, still degrade or import them. Why would they do this? We think there are two possible scenarios: (1) cells might actively take up signal molecules because they provide metabolic benefits or (2) signal molecules might exploit generic uptake machinery of cells. In the first scenario, signal molecules should be metabolically beneficial. In addition, cells should somehow recognize these signal molecules and express the appropriate uptake machinery to import them. If signal molecules are costly to produce for the signal-producing cells and provide metabolic benefits to the surrounding cells, signal production could form a cooperative trait[59]. In the second scenario, signal molecules exploit generic uptake machinery that cells broadly express across the community. In this scenario,

signal molecules must not provide metabolic benefits (although they still could), but simply make use of the fact that cells express generic uptake systems, like oligopeptide permeases, which automatically results in their uptake.

Whether signal uptake comes about through the first or second scenario, for short-range communication to happen, signal molecules should be sufficiently general to be imported by uptake systems in many different bacterial species, which inhabit the community, but–at the same time–be sufficiently specific to allow for communication between signal-producing cells only, without interference of other cells in the community. Peptides are ideal for this purpose, as they can be imported through many generic uptake systems, but also have enough sequence diversity to encode for specific signals. In principle, one expects that lactonases could have worked in the same way in the case of AHL signaling, as specificity is guided by the acyl chain length and side-chain decoration and not by the lactone ring. Yet, most species do not seem to degrade lactones, which makes short-range communication often impossible. It might be that the metabolic benefit of AHL degradation, given their general low concentrations, is insufficient for driving the spread of their degrading enzymes.

2 Mathematical modeling of quorum-sensing designs

2.1 Signal gradient properties: Analytic solutions for various geometries

The aim of this section is to present a general analysis of the impact of signal decay, either caused by signal uptake or signal degradation, on quorum sensing under several different scenarios. As dimensionality is critical in analysis of diffusive processes, we will separately analyze one-, two- and three-dimensional implementations of the model. For each condition, we will consider a cluster of signal-producing cells with a radius R. Like in our experiment, signal-producers harbor the entire quorum-sensing system. The cluster of signal producers is assumed to be embedded within a larger community of cells that do not produce the quorum-sensing signal.

While bacterial communities consist of cells, we use a continuous framework to model the community. That is, instead of modeling cells that produce and receive signal molecules explicitly, we simplify the model by assuming a constant production rate per unit volume and a decay rate per unit volume of the community. This simplification has minimal effect on the modeling

outcome, but nevertheless could make a difference. For example, the effective diffusion coefficient and effective concentration are dependent on the volume fraction filled up by the cells. This is not accounted for in the continuous modeling framework that we adopt here. See more discussion on density dependence in section 2.2 below.

In the continuous framework, the following diffusion equations describe the changes in the signal concentration in the community:

[12] $$\frac{\partial c_{in}}{\partial t} = D\nabla^2 c_{in} + \eta - \alpha_i c_{in} \quad \text{for} \quad r < R$$

[13] $$\frac{\partial c_{out}}{\partial t} = D\nabla^2 c_{out} - \alpha_o c_{out} \quad \text{for} \quad r > R$$

Where r represents the distance from the center of signal-producing cells; η shows the rate of signal production; c_{in} shows signal concentration inside the cluster and c_{out} outside the cluster; α_i shows the rate of signal decay inside the cluster and α_o outside the cluster. We assume that there is a constant production (η) of signal within the cluster ($r < R$) and none outside the cluster ($r > R$). The signal decay rate, caused by either signal degradation or signal uptake, inside (α_i) and outside (α_o) the cluster will depend on the specific quorum-sensing system examined. We assume in our analysis that the signal-producing cluster is embedded in a bacterial community that is much larger than the cluster, having a zero signal concentration in infinity ($c_{out} = 0$ for $r = \infty$). We assume continuity in both the signal concentration and signal fluxes between the inside and outside of the cluster, thereby satisfying the following cluster boundary conditions:

[14] $$c_{in}(R) = c_{out}(R);\ \nabla c_{in}(R) = \nabla c_{out}(R).$$

Our goal is to deduce from equation [12]-[14] the characteristics of the signal concentration gradient for the absorbing and non-absorbing quorum-sensing designs. For each design, we will examine both the intra-cluster and extra-cluster (or inter-cluster) properties:

- **Intra-cluster signal properties**: The signal concentration will vary within the cluster of signal producers, but will always be highest in the center of the cluster. We therefore calculate the both the signal concentration at the center, $c_{in}(0)$, and the average concentration within the cluster, c_{av}, as indicators of the status of signaling within the cluster.

- **Inter-cluster properties**: We are interested in the concentration profile at a distance r from the center of the cluster $c_{out}(r)$ to determine at which spatial distance independent clusters of signal-producing cells within the community could still sense each other. This will depend on the signal decay length-scale, which, as described in equation [6], is the distance over which the signal decays by a factor of e.

If the signal decays with rate α, the signal decay length-scale is given by $\lambda_{signal} = \sqrt{\frac{D}{\alpha}}$. We expect that the signal concentration gradient would depend on this intrinsic length-scale as well as on the geometrical length-scale of the system (i.e. the radius of the cluster, R). It is therefore important to check the solution at different extremes where $R \gg \lambda_{signal}$ and $R \ll \lambda_{signal}$.

2.1.1 Three relevant cases

We will consider three cases, corresponding to the different quorum-sensing systems discussed in section 1.1. We consider both absorbing and non-absorbing quorum-sensing systems and also vary the decay rate of signal molecules outside the cluster of signal producers:

- **Case 1. Non-absorbing systems without signal decay.** We assume that there is no signal decay inside or outside the cluster of producers: $\alpha_i = \alpha_o = 0$.
- **Case 2. Absorbing systems with signal decay inside the cluster only (type B design).** We assume that there is signal decay inside the cluster of signal producers, but not outside. This is a case where the quorum-sensing cells employ absorbing quorum-sensing signaling, but where signal molecules are not taken up or degraded outside the cluster of signal producers: $\alpha_o = 0$.
- **Case 3. Absorbing systems with signal decay inside and outside the cluster (type A design).** We assume signal decay is observed both inside and outside the cluster of signal producers. This corresponds to the absorbing quorum-sensing systems studied in the main text. For simplicity, we assume that $\alpha_i = \alpha_o \neq 0$.

Dimensional considerations. As mentioned above, dimensionality can be critical for analyzing reaction diffusion models. For one- and two-dimensional systems (or three-dimensional systems with a symmetry that can be approximated by one or two-dimensional equations), one can show

that there is no steady state for cases 1 and 2 without a sink boundary condition. That is, if there are no sinks, signal molecules will accumulate indefinitely at the producer region. Analytical solutions describing the signal concentration gradient in these cases are therefore complex and critically dependent on the geometry of the systems (i.e., assumptions on the existence and shape of the boundary conditions). We therefore do not analytically solve cases 1 and 2 for one- or two-dimensional implementations of the model. For these cases, we only consider a three-dimensional scenario. In contrast, for case 3 the concentration profile will reach a steady state for all dimensions. We therefore analyze case 3 for one-, two- and three-dimensional scenarios. Later, in section 2.3, we also examine numerical simulations to explore a two-dimensional scenario that emulates the geometry of a microfluidic chamber for both case 1 (non-absorbing design) and 3 (absorbing design). The results of various scenarios are graphically summarized in Supplementary Fig. 5 and Fig. 1 of the main manuscript.

2.1.2 Analysis of three-dimensional spherical symmetry

In three dimensions, the spherical part of the Laplacian is $\nabla^2 c = \frac{1}{r^2}\left(\frac{d}{dr}\left(r^2\frac{dc}{dr}\right)\right)$, eqs. [12] and [13] can therefore be written explicitly according to the assumptions given in each case.

2.1.2.1 Case 1. Non-absorbing systems without signal decay

The steady-state equation out of the producing region is:

[15] $$D\frac{1}{r^2}\left(\frac{d}{dr}\left(r^2\frac{dc}{dr}\right)\right) = 0 \to c_{out}(r) = \frac{A_o}{r} \text{ for } r > R$$

Within the cluster, the equation is:

[16] $$D\frac{1}{r^2}\left(\frac{d}{dr}\left(r^2\frac{dc}{dr}\right)\right) = -\eta \to c_{in}(r) = A_i - \frac{\eta}{6D}r^2 \text{ for } r < R.$$

To find the specific solution to these equations, we used the continuity of concentration and flux, eq. [14], which corresponds to:

[17] $$\frac{A_o}{R} = A_i - \frac{\eta}{6D}R^2;\ \frac{A_o}{R^2} = \frac{\eta}{3D}R$$

With the solution

[18] $A_o = \frac{\eta}{3D} R^3; A_i = \frac{\eta}{2D} R^2$

The concentration at the center of the cluster is therefore:

[19] $\boldsymbol{c_{in}(0) = \frac{\eta}{2D} R^2 = \frac{3\eta V}{8\pi D R}}$

The average concentration within the cluster is:

[20] $\boldsymbol{c_{av} = \frac{1}{V}\int_0^R 4\pi r^2 c_{in}(r) dr = \frac{3}{R^3}\frac{\eta}{2D}\int_0^R r^2\left(R^2 - \frac{1}{3}r^2\right) dr = \frac{3\eta}{8D} R^2 = \frac{3}{4} c_{in}(0)}$

While out of the cluster, the concentration is:

[21] $\boldsymbol{c_{out}(r) = \frac{\eta}{3D} R^3 \frac{1}{r} = \frac{\eta V}{4\pi D r}}$

V is the volume of the sphere and ηV is the total signal produced per time unit in the cluster.

The signal decay length-scale of the system is:

[22] $\lambda_{signal} = -\left(\frac{d log(c_{out})}{dr}\right)^{-1} = r$

That is, there is no clear intrinsic length-scale in this system and decay is simply proportional to the distance itself.

2.1.2.2 Case 2. Absorbing systems with signal decay inside the cluster only (type B design)

In case 2, the concentration function outside the cluster is the same as in case 1 (eq. [15]), but the concentration within the sphere follows the equation:

[23] $D\nabla^2 c + \eta - \alpha c = 0$

By renormalizing r by the signal decay length-scale $\lambda = \sqrt{\frac{D}{\alpha}}$ and setting $y = \frac{\eta}{\alpha} - c$, this equation reduces to, $\nabla^2 y - y = 0$, Which can be solved to yield as a finite solution:

[24] $c_{in}(r) = \frac{\eta}{\alpha} - A_i \frac{\lambda}{r} \sinh\left(\frac{r}{\lambda}\right)$

The boundary conditions on the sphere now yields:

[25] $A_i \left(R\cosh\left(\frac{R}{\lambda}\right) - \lambda \sinh\left(\frac{R}{\lambda}\right)\right) = A_o$

[26] $$\frac{\eta}{\alpha} - A_i \frac{\lambda}{R} \sinh\left(\frac{R}{\lambda}\right) = \frac{A_o}{R}$$

[27] $$A_i = \frac{\eta}{\alpha} \frac{1}{\cosh\left(\frac{R}{\lambda}\right)}; A_o = \frac{\eta}{\alpha}\left(R - \lambda \tanh\left(\frac{R}{\lambda}\right)\right)$$

Yielding a concentration at the center of the cluster:

[28] $$\boldsymbol{c_{in}(0) = \frac{\eta}{\alpha}\left(1 - \frac{1}{\cosh\left(\frac{R}{\lambda}\right)}\right) \sim \begin{cases} \frac{\eta}{\alpha} & R \gg \lambda \\ \frac{\eta}{2D} R^2 & R \ll \lambda \end{cases}}$$

And:

[29] $$\boldsymbol{c_{av} = \frac{\eta}{\alpha}\lambda\left[1 - \frac{3}{4\pi R^3}\int_0^{\frac{R}{\lambda}}\left(\frac{1}{cosh\left(\frac{R}{\lambda}\right)}\frac{\sinh(y)}{y}\right)dy\right]}$$

And out of the sphere:

[30] $$\boldsymbol{c_{out}(r) = \frac{\eta}{\alpha}\frac{\left(R - \lambda \tanh\left(\frac{R}{\lambda}\right)\right)}{r} = \frac{\eta}{D}\frac{\left(R\lambda^2 - \lambda^3 \tanh\left(\frac{R}{\lambda}\right)\right)}{r} \sim \frac{\eta}{Dr} \times \begin{cases} R\lambda^2 & R \gg \lambda \\ \frac{1}{3}R^3 & R \ll \lambda \end{cases}}$$

For each of the values, we present the limits where the signal decay length-scale is very small or very large compared to cluster radius. In the case where the decay length-scale is very large compared to cluster size, we find that the effect of signal decay is negligible, and the equations reduce to those of the first case. In the case where the decay length-scale is very small, we find that the concentration inside the cluster approaches the limit $\frac{\eta}{\alpha}$, while the concentration outside the cluster is smaller by a factor $\sim \frac{\lambda^2}{R^2}$ than the concentration expected if the cluster was a non-absorbing system.

2.1.2.3 Case 3. Absorbing systems with signal decay inside and outside the cluster (type A design)

The equation for the concentration inside the cluster remains the same as in eqs. [23] and [24], while the concentration out of the cluster can be shown to behave as:

[31] $$\boldsymbol{c_{out}(r) = A_o \lambda \frac{e^{-r/\lambda}}{r}}$$

The boundary conditions on the cluster lead to:

[32] $$A_o = \frac{\eta}{\alpha}\left(\frac{R}{\lambda}\cosh\left(\frac{R}{\lambda}\right) - \sinh\left(\frac{R}{\lambda}\right)\right)$$

[33] $$A_i = \frac{\eta}{\alpha}\left(\frac{R}{\lambda} + 1\right)e^{-\frac{R}{\lambda}}$$

Which yields:

[34] $$\boldsymbol{c_{in}(0) = \frac{\eta}{\alpha}\left(1 - \left(\frac{R}{\lambda} + 1\right)e^{-\frac{R}{\lambda}}\right) \sim \begin{cases} \frac{\eta}{\alpha} & R \gg \lambda \\ \frac{\eta}{2D}R^2 & R \ll \lambda \end{cases}}$$

[35] $$\boldsymbol{c_{out}(r) = \frac{\eta}{\alpha}\left(\frac{R}{\lambda}\cosh\left(\frac{R}{\lambda}\right) - \sinh\left(\frac{R}{\lambda}\right)\right)\frac{\lambda}{r}e^{-\frac{r}{\lambda}} \sim \begin{cases} \frac{\eta}{\alpha}\frac{R}{r}e^{-\frac{r-R}{\lambda}} = \frac{\eta}{Dr}R\lambda^2 e^{-\frac{r-R}{\lambda}} & R \gg \lambda \\ \frac{\eta}{3Dr}R^3 e^{-\frac{r}{\lambda}} & R \ll \lambda \end{cases}}$$

The length of the concentration gradient behaves almost exactly like $\lambda = \sqrt{\frac{D}{\alpha}}$:

[36] $$\lambda_{signal} = -\left(\frac{d\log(c_{out})}{dr}\right)^{-1} = \left(\frac{1}{r} + \frac{1}{\lambda}\right)^{-1} \sim \begin{cases} \lambda & r > R \gg \lambda \\ \mathrm{r} & R < r \ll \lambda \end{cases}$$

We find here that the concentration in the cluster center behaves as in case 2 – it is independent of cluster radius if this is large compared to the signal decay length-scale and behaves as in case 1 when the cluster radius is smaller than the length-scale. Out of the cluster it goes down exponentially with the signal decay length-scale, but if the length-scale is larger than the cluster radius, it will behave as a freely diffusing gradient near the cluster.

2.1.3 Solving case 3 in one and two dimensions

For case 3, we can solve the steady-state equation also for a two-dimensional (cylindrical symmetry) and one-dimensional (linear symmetry) scenarios.

Two dimensions: In a two-dimensional cylindrical symmetry, the radial part of the Laplacian can be written as, $\nabla^2 c = \frac{1}{r}\frac{d}{dr}\left(r\frac{dc}{dr}\right)$. The resulting equations for the concentration inside and outside of the producing cluster now have the form of the modified Bessel equation with $n = 0$ (that is, $x^2\frac{d^2y}{dx^2} + x\frac{dy}{dx} - (x^2 + n^2) = 0$) for either c itself (out of the circle), or for $\frac{\eta}{\alpha} - c$ within the circle. The modified Bessel equation has two solutions, a monotonically increasing solution known as $I_n(x)$, the modified Bessel function of the first kind, and a monotonically decreasing solution known as $K_n(x)$, the modified Bessel function of the second kind.

We find that the solution for the system of equations is:

[37] $$\boldsymbol{c_{in}(r) = \frac{\eta}{\alpha}\left(1 - \Phi_1\left(\frac{R}{\lambda}\right) I_0\left(\frac{r}{\lambda}\right)\right); \quad \Phi_1(x) = \left(\frac{I_0(x)}{I_0'(x)} - \frac{K_0(x)}{K_0'(x)}\right)^{-1} \frac{1}{I_0'(x)}}$$

[38] $$\boldsymbol{c_{out}(r) = \frac{\eta}{\alpha}\Phi_2\left(\frac{R}{\lambda}\right) K_0\left(\frac{r}{\lambda}\right); \quad \Phi_2(x) = \left(\frac{I_0(x)}{I_0'(x)} - \frac{K_0(x)}{K_0'(x)}\right)^{-1} \frac{1}{K_0'(x)}}$$

One can show that at the limits we find:

[39] $$\boldsymbol{c_{in}(0) = \frac{\eta}{\alpha}\left(1 - \Phi_1\left(\frac{R}{\lambda}\right)\right) = \begin{cases} \frac{\eta}{\alpha} & R \gg \lambda \\ \frac{\eta}{2D} R^2 \left(\gamma - \log\left(\frac{R}{\lambda}\right)\right) & R \ll \lambda \end{cases}}$$

The second modified Bessel function behaves at a large value as, $K_0(x) \sim 0.4 \times \frac{e^{-x}}{\sqrt{x}}$. We find that for $R \ll \lambda$, $\Phi_2\left(\frac{R}{\lambda}\right) = \frac{1}{2}\left(\frac{R}{\lambda}\right)^2$ implying that

[40] $$\boldsymbol{c_{out}(r) = 0.2 \times \frac{\eta}{D} R^2 \times \frac{e^{-\frac{r}{\lambda}}}{\sqrt{\frac{r}{\lambda}}} \text{ for } r \gg \lambda.}$$

The length-scale now behaves as:

[41] $$\lambda_{signal} = \left(\frac{1}{2r} + \frac{1}{\lambda}\right)^{-1} \sim \begin{cases} \lambda & r > R \gg \lambda \\ 2\mathrm{r} & R < r \ll \lambda \end{cases}$$

One dimension: One way to solve the diffusion equation with degradation in one dimension is by integrating over the appropriate Green function, where there is a delta function of production, $\eta(x) = \eta\delta(r - r_0)$:

[42] $$G(x, x_0) = \frac{1}{2}\frac{\eta}{D\lambda} e^{-\frac{|r - r_0|}{\lambda}}; \lambda = \sqrt{\frac{D}{\alpha_0}}$$

Within the producing cluster $(-R < r < R)$ we find:

[43] $$c_{in}(r) = \int_{-R}^{R} G(r - r_0) dx_0 = \frac{1}{2}\eta\frac{\lambda}{D}\left[\int_{-R}^{r} e^{\frac{(r_0 - r)}{\lambda}} dr_0 + \int_{r}^{R} e^{-\frac{(r_0 - r)}{\lambda}} dr_0\right] = \frac{1}{2}\frac{\eta}{\alpha}\left[\left[1 - \exp\left(-\frac{(R + |r|)}{\lambda}\right)\right] - \left[\exp\left(-\frac{(R - |r|)}{\lambda}\right) - 1\right]\right] = \frac{\eta}{\alpha}\left[1 - \frac{\exp\left(-\frac{(R+|r|)}{\lambda}\right) + \exp\left(-\frac{(R-|r|)}{\lambda}\right)}{2}\right]$$

If $R \gg \lambda$ this reduces to:

[44] $$\boldsymbol{c_{in}(0)} = \begin{cases} \frac{\eta}{\alpha} & R \gg \lambda \\ \frac{\eta}{\alpha}\frac{R}{\lambda} & R \ll \lambda \end{cases}$$

We can also calculate the average concentration in the producer cluster:

[45] $$c_{av} = \frac{\eta}{2\alpha R}\int_{-R}^{R}\left[1 - \frac{\exp\left(-\frac{(R+|r|)}{\lambda}\right)+\exp\left(-\frac{(R-|r|)}{\lambda}\right)}{2}\right]dr = \frac{\eta}{\alpha R}\left[r + \frac{\lambda}{2}\left(\exp\left(-\frac{(R+|r|)}{\lambda}\right) - \exp\left(-\frac{(R-|r|)}{\lambda}\right)\right)\right]_0^R = \frac{\eta}{\alpha R}\left[R + \frac{\lambda}{2}\left(\exp\left(-\frac{2R}{\lambda}\right) - 1\right)\right] \cong \left\{\frac{\eta}{\alpha_i \lambda}R = c_{in}(0) \quad \text{for } R \ll \lambda\right.$$

To a first approximation, the maximum level and average levels are the same, as the concentration is fairly flat within the cluster for $R \ll \lambda$.

Out of the cluster ($|r| > R$), we find:

[46] $$c_{out}(r) = \int_{-R}^{R} G(r - r_0)dr_0 = \frac{1}{2}\frac{\lambda}{D}\int_{-R}^{R} e^{-\frac{(r-r_0)}{\lambda}}dr_0 = -\eta\frac{\lambda^2}{D}\left[e^{-\frac{(r-r_0)}{\lambda}}\right]_{-R}^{R} = \frac{\eta}{\alpha_0}\exp\left(-\frac{|r|}{\lambda}\right)\frac{\exp\left(\frac{R}{\lambda}\right)-\exp\left(-\frac{R}{\lambda}\right)}{2} \cong \begin{cases} \frac{1}{2}\frac{\eta}{\alpha_0}\exp\left(-\frac{|r|-R}{\lambda}\right) & R \gg \lambda \\ R\frac{\eta}{\alpha_o \lambda}\exp\left(-\frac{|r|}{\lambda}\right) & R \ll \lambda \end{cases}$$

If cluster size is larger than λ, the system behaves as if all production is at the right side of the region with $\eta_{tot} = \lambda\eta$. If it smaller than λ, the system behaves as if all production is at the center with $\eta_{tot} = 2R\eta$.

2.2 The dependence of quorum-sensing signaling on cell density

In the above section we analyzed how various geometrical scenarios affect the quorum-sensing signal gradients, given a fixed cell density. In this section, we explore how the cell density itself can affect the signal gradient.

A variety of parameters depend on the cell density, d,which we will measure here as the volume fraction taken by cells out of the total volume. The main two parameters which linearly depend on cell density are the production rate and uptake rate per unit volume, which are the parameters we have used in the previous section:

[47] $$\eta = \frac{\eta_{cell}}{n_{cell}}d; \quad \alpha = \frac{\alpha_{cell}}{n_{cell}}d$$

Where η_{cell} and α_{cell} are the production rate and uptake rate *per cell* respectively and d_{cell} is the volume fraction of a single cell. The other two parameters which depend on cell density are the effective diffusion and effective concentration, both only weakly depending on the cell density when d is much smaller than 1 $(d \ll 1)$:

[48] $$D_{eff} = D\frac{1-d}{1+\frac{1}{2}d} \sim D\left(1-\frac{3}{2}d\right), \quad c_{eff} = c\frac{1}{1-d} \sim c(1-d)$$

We will ignore these two corrections and follow the implication of the effect of density on production and decay only.

First, we note the effect of cell density on the main effective coefficients arising in the solutions:

[49] Decay length scale: $\lambda = \sqrt{\frac{D}{\alpha}} = \sqrt{\frac{Dd_{cell}}{\alpha_{cell}}} \times \frac{1}{\sqrt{d}} \propto d^{-\frac{1}{2}}$;

[50] Maximal concentration in the presence of signal decay: $\frac{\eta}{\alpha} = \frac{\eta_{cell}}{\alpha_{cell}} \propto d^0$;

[51] Maximal concentration in the absence of signal decay: $\frac{\eta}{D} = \frac{\eta_{cell}}{Dd_{cell}} d \propto d^1$

We can now compare the concentration dependence on cell density for case 1 (non-absorbing design) and cases 2,3 (absorbing designs) in three dimensions:

[52] $c_{in}(0) = \frac{\eta}{2D}R^2 \propto d^1$ case 1

[53] $$c_{in}(0) \sim \begin{cases} \frac{\eta}{\alpha} \propto d^0 & R \gg \lambda \\ \frac{\eta}{2D}R^2 \propto d^1 & R \ll \lambda \end{cases} \quad \text{in cases 2,3}$$

We find that when signal decay is negligible (either case 1, or cases 2,3 when $R \ll \lambda$) the concentration inside the cluster depend on cell density, as expected from a quorum-sensing system. In contrast, when signal decay is strong (e.g. high signal uptake rates in the absorbing quorum-sensing design), the balance between production and decay renders the concentration inside the cluster cell-density independent, as was predicted before for well-mixed conditions[48].

Out of the cluster, the signal concentration gradient for case 3, where signal decay occurs both within and outside the signal producing cluster, behaves as:

[54] $$c_{out}(\mathrm{r}) = \frac{\eta}{Dr}R\lambda^2 \times e^{-\frac{r-R}{\lambda}}$$

The pre-factor is independent of the density, but the exponent decay length scale behaves as $d^{-\frac{1}{2}}$. For case 1, where signal does not decay, we find:

[55] $$c_{out}(r) = \frac{\eta}{3D} R^3 \frac{1}{r} \propto d^1$$

The system behaves like expected from a quorum-sensing system, with a signal proportional to the density in every position.

Another interesting question is how cell density (d) affects the minimal number of cells in a cluster (N) and the minimal cluster size (R) that is needed to obtain a certain signal concentration threshold in the middle of the cluster ($c_{in}(0)$). We can use eqs. [52], [53] to estimate the required cluster size and total number of cells:

For case 1, without signal decay, reaching a concentration c_{th} requires a cluster radius (R_{th}) and a number of cells in cluster (N_{th}):

[56] $$c_{in}(0) = c_{th} \rightarrow R_{th} = \sqrt{\frac{2Dc_{th}}{\eta}} \propto d^{-\frac{1}{2}}, \;\; N_{th} = \frac{4\pi}{3} R_{th}^3 d \propto d^{-\frac{1}{2}}$$

For the case 3, with decay in the entire community, reaching a saturated signal response c_{max} requires a cluster radius (R_{th}) and a number of cells in the cluster (N_{th}):

[57] $$c_{in}(0) = c_{max} \rightarrow R_{th} \cong \lambda \propto d^{-\frac{1}{2}}, \;\; N_{th} = \frac{4\pi}{3} R_{th}^3 d \propto d^{-\frac{1}{2}}$$

Therefore, as the density of cells *increases*, the minimal size of the cluster (R_{th}) and the number of cells in this cluster (N_{th}) needed to obtain a given signal concentration (or the maximum signal concentration, as determined for case 3) *decrease* by a factor of square root of the density irrespective if there is signal decay or not (case 1 or 3).

We note that for a two-dimensional cylindrical symmetry in the presence of signal decay (case 3) the minimal number of cells needed to obtain a given signal concentration in a cluster with radius R_{th} behaves differently:

[58] $$R_{th} = \lambda \propto d^{-\frac{1}{2}}, N_{th} = \pi R_{th}^2 \times \mathrm{n} \propto d^0$$

That is, the minimal number of cells which lead to maximal concentration is constant and independent of the density.

2.3 Numerical analysis of signal gradient properties in microfluidics chambers

The microfluidic chamber we use to experimentally study signal propagation are $100\mu m \times 60\mu m$ (chamber width x chamber height, see Supplementary Fig. 5c) with one of the long sides open to the flow channel and the other three sides being closed. To a good approximation, we can assume this sets a sink boundary condition (i.e, $c = 0$) on the open side and a closed boundary condition ($n \cdot \nabla c = 0$) on the other three sides. For the numeric analysis of signal propagation in the chamber, we assume that the chamber dimension are $L_1 \times L_2$, where L_1 is the chamber width and L_2 the height (Supplementary Fig. 5c). For simplicity, the chambers examined in Supplementary Fig. 5 are twice as wide ($L_1 = 100\mu m$) than high ($L_2 = 50\mu m$). We assume that the quorum-sensing cells occupy one side of the chamber with size $w \times L_2$. We also assume that the signal decays in the entire chamber, like for the absorbing quorum-sensing systems examined in the main text (case 3 discussed above) with a decay length-scale, λ. The equations guiding the steady-state distribution of the signal are:

[59] $0 = \nabla^2 \mathrm{c} + \frac{\eta}{D} - \lambda^2 \mathrm{c}$ Within the region of signal producers

[60] $0 = \nabla^2 \mathrm{c} - \lambda^2 \mathrm{c}$ Outside the region of signal producers

[61] $n \cdot \nabla c$ Over the closed sides

[62] $c = 0$ on the channel side.

Note that with no loss of generality, we can normalize concentration with $\frac{\eta}{D} = 1$. We can also normalize all lengths to the chamber width (L_1), but will not do it, as we wish to demonstrate the actual length-scales obtained for a chamber of size $100\mu m$.

We use the Matlab *Partial Differentiation Equation Toolbox* to calculate the distribution of the signal concentration. Supplementary Fig. 5e shows a solution for the 2D distribution of the signal for the cases where $\lambda = 5\mu m, 15\mu m, 100\mu m$. The corresponding profiles at the internal most side of the chamber (along the L_1 side opposite to the main channel) are shown on a logarithmic axis in Supplementary Fig. 5d (left panel). We define the effective length-scale of the signal

concentration gradient (i.e. communication range) as the minimal value of $\lambda_{eff} = -\left(\frac{dlogc}{dx}\right)^{-1}$ along the back of the chamber. Supplementary Fig. 5d (middle panel) shows the effective length-scale λ_{eff} (i.e. communication range) as a function of the decay length-scale, λ. We find that for small λ the two are equal, but when $\lambda \gtrsim 30\mu m$, λ_{eff} saturates at a value of $\sim 40\mu m$. We also show that the saturation level depends on the height of the chamber: saturation in λ_{eff} occurs for higher decay length-scales (λ) in larger chambers.

Finally, Supplementary Fig. 5d (right panel) shows the mean level of expression within the signal producing cell cluster as a function of the width of the cluster, for the length-scales $\lambda = 5\mu m, 15\mu m, 100\mu m$. These are compared to the expected concentration profile of a 1D system with degradation given by an appropriately normalized graph of eq. [45]. As can be seen, the concentration profile within the chamber fits well with the 1D model expectations for shorter decay length-scales, but not for the longer decay length-scales.

2.4 Absorbing systems and the averaging distance of neighbor frequency

As argued in the main text, the mobile-element associated systems are measuring the average number of neighbors producing the signal over a range which is related to the decay length-scale of the absorbing system. Here we provide the expression for this averaging term. We assume that cells are populating an area with a constant density d_c, but that the frequency of the signal producers varies in space, $f_p(\vec{r})$. The concentration of signal in every position can be calculated from the Green function of an infinitesimal producer:

[63] $$G(r';r) = \frac{\eta f(r)}{4\pi D(r'-r)} e^{-\frac{r'-r}{\lambda}} dV; \lambda = \sqrt{\frac{D}{\alpha d_c}}$$

The concentration at a given position r is therefore:

[64] $$c(r) = \int G(r;r') dV' = \frac{\eta}{4\pi D} \int \frac{f(r\prime)}{(r-r\prime)} e^{-\frac{r-r\prime}{\lambda}} dV'$$

$c(r)$ is therefore averaging the frequency of producer cells in the region of r with a weight factor of $\frac{1}{(r-r\prime)} e^{-\frac{r-r\prime}{\lambda}}$, which is exponentially decreasing with the distance from the measured position with a length-scale λ.

References

1. Magnuson, R., Solomon, J. & Grossman, A. D. Biochemical and genetic characterization of a competence pheromone from *B. subtilis*. *Cell* **77**, 207–216 (1994).
2. Schneider, K. B., Palmer, T. M. & Grossman, A. D. Characterization of *comQ* and *comX*, two genes required for production of ComX pheromone in *Bacillus subtilis*. *J Bacteriol* **184**, 410–419 (2002).
3. Håvarstein, L. S., Gaustad, P., Nes, I. F. & Morrison, D. A. Identification of the streptococcal competence-pheromone receptor. *Mol Microbiol* **21**, 863–869 (1996).
4. Ji, G., Beavis, R. & Novick, R. P. Bacterial interference caused by autoinducing peptide variants. *Science* **276**, 2027–2030 (1997).
5. Novick, R. P. & Geisinger, E. Quorum sensing in Staphylococci. *Annu Rev Genet* **42**, 541–564 (2008).
6. Wuster, A. & Babu, M. M. Conservation and evolutionary dynamics of the *agr* cell-to-cell communication system across Firmicutes. *J Bacteriol* **190**, 743–746 (2008).
7. Henke, J. M. & Bassler, B. L. Three parallel quorum-sensing systems regulate gene expression in *Vibrio harveyi*. *J Bacteriol* **186**, 6902–6914 (2004).
8. Ng, W. L. & Bassler, B. L. Bacterial quorum-sensing network architectures. *Annu Rev Genet* **43**, 197–222 (2009).
9. Ansaldi, M., Marolt, D., Stebe, T., Mandic-Mulec, I. & Dubnau, D. Specific activation of the *Bacillus* quorum-sensing systems by isoprenylated pheromone variants. *Mol Microbiol* **44**, 1561–1573 (2002).
10. Stefanic, P. *et al.* The quorum sensing diversity within and between ecotypes of *Bacillus subtilis*. *Environ Microbiol* **14**, 1378–1389 (2012).
11. Papenfort, K. & Bassler, B. L. Quorum sensing signal-response systems in gram-negative bacteria. *Nat Rev Microbiol* **14**, 576–588 (2016).
12. Schuster, M., Joseph Sexton, D., Diggle, S. P. & Peter Greenberg, E. Acyl-homoserine lactone quorum sensing: from evolution to application. *Annu Rev Microbiol* **67**, 43–63 (2013).
13. Lazazzera, B. A. & Grossman, A. D. The ins and outs of peptide signaling. *Trends Microbiol* **6**, 288–294 (1998).
14. Monnet, V. & Gardan, R. Quorum-sensing regulators in gram-positive bacteria: 'cherchez le peptide'. *Mol Microbiol* **97**, 181–184 (2015).
15. Perego, M. & Hoch, J. A. Cell-cell communication regulates the effects of protein aspartate phosphatases on the phosphorelay controlling development in *Bacillus subtilis*. *PNAS* **93**, 1549–1553 (1996).
16. Solomon, J. M., Lazazzera, B. A. & Grossman, A. D. Purification and characterization of an extracellular peptide factor that affects two different developmental pathways in *Bacillus subtilis*. *Genes Dev* **10**, 2014–2024 (1996).

17. Lanigan-Gerdes, S., Dooley, A. N., Faull, K. F. & Lazazzera, B. A. Identification of subtilisin, Epr and Vpr as enzymes that produce CSF, an extracellular signalling peptide of *Bacillus subtilis*. *Mol Microbiol* **65**, 1321–1333 (2007).
18. Stephenson, S., Mueller, C., Jiang, M. & Perego, M. Molecular analysis of Phr peptide processing in *Bacillus subtilis*. *J Bacteriol* **185**, 4861–4871 (2003).
19. Perego, M. A peptide export–import control circuit modulating bacterial development regulates protein phosphatases of the phosphorelay. *PNAS* **94**, 8612–8617 (1997).
20. Even-Tov, E., Omer Bendori, S., Pollak, S. & Eldar, A. Transient duplication-dependent divergence and horizontal transfer underlie the evolutionary dynamics of bacterial cell–cell signaling. *PLoS Biol* **14**, e2000330 (2016).
21. Higgins, C. F. & Gibson, M. M. Peptide transport in bacteria. *Methods Enzymol* **125**, 365–377 (1986).
22. Schauder, S. & Bassler, B. L. The languages of bacteria. *Genes Dev* **15**, 1468–1480 (2001).
23. Xavier, K. B. & Bassler, B. L. LuxS quorum sensing: more than just a numbers game. *Curr Opin Microbiol* **6**, 191–197 (2003).
24. Taga, M. E., Semmelhack, J. L. & Bassler, B. L. The LuxS-dependent autoinducer AI-2 controls the expression of an ABC transporter that functions in AI-2 uptake in *Salmonella typhimurium*. *Mol Microbiol* **42**, 777–793 (2001).
25. Xavier, K. B. & Bassler, B. L. Regulation of uptake and processing of the quorum-sensing autoinducer AI-2 in *Escherichia coli*. *J Bacteriol* **187**, 238–248 (2005).
26. Rezzonico, F. & Duffy, B. Lack of genomic evidence of AI-2 receptors suggests a non-quorum sensing role for *luxS* in most bacteria. *BMC Microbiol* **8**, 154 (2008).
27. Chen, X. *et al.* Structural identification of a bacterial quorum-sensing signal containing boron. *Nature* **415**, 545–549 (2002).
28. Piazza, F., Tortosa, P. & Dubnau, D. Mutational analysis and membrane topology of ComP, a quorum-sensing histidine kinase of *Bacillus subtilis* controlling competence development. *J Bacteriol* **181**, 4540–4548 (1999).
29. Comella, N. & Grossman, A. D. Conservation of genes and processes controlled by the quorum response in bacteria: characterization of genes controlled by the quorum-sensing transcription factor ComA in *Bacillus subtilis*. *Mol Microbiol* **57**, 1159–1174 (2005).
30. Lazazzera, B. A., Solomon, J. M. & Grossman, A. D. An exported peptide functions intracellularly to contribute to cell density signaling in *B. subtilis*. *Cell* **89**, 917–925 (1997).
31. D'Souza, C., Nakano, M. M. & Zuber, P. Identification of *comS*, a gene of the *srfA* operon that regulates the establishment of genetic competence in *Bacillus subtilis*. *PNAS* **91**, 9397–9401 (1994).
32. Tortosa, P. *et al.* Specificity and genetic polymorphism of the *Bacillus* competence quorum-sensing system. *J Bacteriol* **183**, 451–460 (2001).

33. Tran, L. S. P., Nagai, T. & Itoh, Y. Divergent structure of the ComQXPA quorum-sensing components: molecular basis of strain-specific communication mechanism in *Bacillus subtilis*. *Mol Microbiol* **37**, 1159–1171 (2000).
34. Slamti, L. & Lereclus, D. A cell-cell signaling peptide activates the PlcR virulence regulon in bacteria of the *Bacillus cereus* group. *EMBO J* **21**, 4550–4559 (2002).
35. Gohar, M. *et al.* The PlcR virulence regulon of *Bacillus cereus*. *PLoS One* **3**, e2793 (2008).
36. Bouillaut, L. *et al.* Molecular basis for group-specific activation of the virulence regulator PlcR by PapR heptapeptides. *Nucleic Acids Res* **36**, 3791–3801 (2008).
37. Rocha-Estrada, J., Aceves-Diez, A. E., Guarneros, G. & de la Torre, M. The RNPP family of quorum-sensing proteins in gram-positive bacteria. *Appl Microbiol Biotechnol* **87**, 913–923 (2010).
38. Bendori, S. O., Pollak, S., Hizi, D. & Eldar, A. The RapP-PhrP quorum-sensing system of *Bacillus subtilis* strain NCIB3610 affects biofilm formation through multiple targets, due to an atypical signal-insensitive allele of RapP. *J Bacteriol* **197**, 592–602 (2015).
39. Parashar, V., Jeffrey, P. D. & Neiditch, M. B. Conformational change-induced repeat domain expansion regulates Rap phosphatase quorum-sensing signal receptors. *PLoS Biol.* **11**, e1001512 (2013).
40. Solomon, J., Su, L., Shyn, S. & Grossman, A. D. Isolation and characterization of mutants of the *Bacillus subtilis* oligopeptide permease with altered specificity of oligopeptide transport. *J Bacteriol* **185**, 6425–6433 (2003).
41. Koide, A. & Hoch, J. A. Identification of a second oligopeptide transport system in *Bacillus subtilis* and determination of its role in sporulation. *Mol Microbiol* **13**, 417–426 (1994).
42. Koide, A., Perego, M. & Hoch, J. A. ScoC regulates peptide transport and sporulation initiation in *Bacillus subtilis*. *J Bacteriol* **181**, 4114–4117 (1999).
43. Erez, Z. *et al.* Communication between viruses guides lysis–lysogeny decisions. *Nature* **541**, 488–493 (2017).
44. Stokar-Avihail, A., Tal, N., Erez, Z., Lopatina, A. & Sorek, R. Widespread utilization of peptide communication in phages infecting soil and pathogenic bacteria. *Cell Host Microbe* **25**, 746-755.e5 (2019).
45. Auchtung, J. M., Lee, C. A., Monson, R. E., Lehman, A. P. & Grossman, A. D. Regulation of a *Bacillus subtilis* mobile genetic element by intercellular signaling and the global DNA damage response. *PNAS* **102**, 12554–12559 (2005).
46. Bose, B., Auchtung, J. M., Lee, C. A. & Grossman, A. D. A conserved anti-repressor controls horizontal gene transfer by proteolysis. *Mol Microbiol* **70**, 570–582 (2008).
47. Eldar, A., Rosin, D., Shilo, B.-Z. & Barkai, N. Self-enhanced ligand degradation underlies robustness of morphogen gradients. *Dev Cell* **5**, 635–646 (2003).
48. Babel, H. *et al.* Ratiometric population sensing by a pump-probe signaling system in *Bacillus subtilis*. *Nat Commun* **11**, 1–13 (2020).

49. Levdikov, V. M. *et al.* The structure of the oligopeptide-binding protein, AppA, from *Bacillus subtilis* in complex with a nonapeptide. *J Mol Biol* **345**, 879–892 (2005).
50. Bareia, T., Pollak, S. & Eldar, A. Self-sensing in *Bacillus subtilis* quorum-sensing systems. *Nat Microbiol* **3**, 83–89 (2018).
51. Henkel, M. *et al.* Kinetic modeling of the time course of *N*-butyryl-homoserine lactone concentration during batch cultivations of *Pseudomonas aeruginosa* PAO1. *Appl Microbiol Biotechnol* **97**, 7607–7616 (2013).
52. Sio, C. F. *et al.* Quorum quenching by an *N*-acyl-homoserine lactone acylase from *Pseudomonas aeruginosa* PAO1. *Infect Immun* **74**, 1673–1682 (2006).
53. Wahjudi, M. *et al.* PA0305 of *Pseudomonas aeruginosa* is a quorum quenching acylhomoserine lactone acylase belonging to the Ntn hydrolase superfamily. *Microbiology* **157**, 2042–2055 (2011).
54. Englmann, M. *et al.* The hydrolysis of unsubstituted *N*-acylhomoserine lactones to their homoserine metabolites: analytical approaches using ultra performance liquid chromatography. *J Chromatogr A* **1160**, 184–193 (2007).
55. Dong, Y. H. *et al.* Quenching quorum-sensing-dependent bacterial infection by an *N*-acyl homoserine lactonase. *Nature* **411**, 813–817 (2001).
56. Buddrus-Schiemann, K. *et al.* Analysis of *N*-acylhomoserine lactone dynamics in continuous cultures of *Pseudomonas putida* IsoF by use of ELISA and UHPLC/qTOF-MS-derived measurements and mathematical models. *Anal Bioanal Chem* **406**, 6373–6383 (2014).
57. Fekete, A. *et al.* Dynamic regulation of *N*-acyl-homoserine lactone production and degradation in *Pseudomonas putida* IsoF. *FEMS Microbiol Ecol* **72**, 22–34 (2010).
58. Danino, T., Mondragón-Palomino, O., Tsimring, L. & Hasty, J. A synchronized quorum of genetic clocks. *Nature* **463**, 326–330 (2010).
59. West, S. A., Griffin, A. S. & Gardner, A. Social semantics: altruism, cooperation, mutualism, strong reciprocity and group selection. *J Evol Biol* **20**, 415–432 (2007).
