# Supplementary material for: Short-range quorum sensing controls horizontal gene transfer at micron scale in bacterial communities
Source: Nat Commun. 2021 Apr 19;12:2324. doi: 10.1038/s41467-021-22649-4 (PMC8055654; doi:10.1038/s41467-021-22649-4)
Supplement: Supplementary file 2 — Description of Additional Supplementary Files [file 41467_2021_22649_MOESM2_ESM.pdf]

Description of Additional Supplementary Files

File Name: Supplementary Movie 1
Description: Growth chamber of non-absorbing ComQXP quorum-sensing system, with signal producers (BFP, here shown in cyan) and signal receiving cells (RFP). Signal response is shown by the expression of YFP. From top to bottom: BFP and RFP channel; BFP and YFP channel; YFP channel.

File Name: Supplementary Movie 2
Description: Growth chamber of absorbing PlcR-PapR quorum-sensing system, with signal producers (BFP, here shown in cyan) and signal receiving cells (RFP). Signal response is shown by the expression of YFP. From top to bottom: BFP and RFP channel; BFP and YFP channel; YFP channel.

File Name: Supplementary Movie 3
Description: Growth chamber of absorbing RapP-PhrP quorum-sensing system, with signal producers (BFP, here shown in cyan) and signal receiving cells (RFP). Signal response is shown by the expression of YFP. From top to bottom: BFP and RFP channel; BFP and YFP channel; YFP channel.

File Name: Supplementary Movie 4
Description: Growth chamber of absorbing arbitrium quorum-sensing system of phage ϕ3T, with signal producers (BFP, here shown in cyan) and signal receiving cells (RFP). Quorum-sensing signal inhibits GFP expression (here shown in yellow), indicative for activation of lytic lifestyle. From top to bottom: BFP and RFP channel; BFP and GFP channel; GFP channel.

File Name: Supplementary Movie 5
Description: Growth chamber of absorbing RapI-PhrI quorum-sensing system of regulatory-only ICE*Bs1* element, with signal producers (BFP, here shown in cyan) and signal receiving cells (RFP). Quorum-sensing signal inhibits YFP expression, indicative for activation of conjugation. From top to bottom: BFP and RFP channel; BFP and YFP channel; YFP channel.

File Name: Supplementary Movie 6
Description: Growth chamber with donor cells (BFP, here shown in cyan) and recipient cells (RFP). Donor cells only contain regulatory part of ICEBs1 system, including the full RapI-PhrI quorum-sensing system. YFP expression shows induction of conjugation in host cells. From top to bottom: BFP and RFP channel; BFP and YFP channel; YFP channel.

File Name: Supplementary Movie 7
Description: Growth chamber with donor cells (BFP, here shown in cyan) and recipient cells (RFP). Donor cells contain Δ*conB* ICE*Bs1* element. YFP expression shows induction of conjugation in host cells. From top to bottom: BFP and RFP channel; BFP and YFP channel; YFP channel.

File Name: Supplementary Data 1
Description: Image data and Matlab scripts.
