# Supplementary material for: Short-range quorum sensing controls horizontal gene transfer at micron scale in bacterial communities
Source: Nat Commun. 2021 Apr 19;12:2324. doi: 10.1038/s41467-021-22649-4 (PMC8055654; doi:10.1038/s41467-021-22649-4)
Supplement: Supplementary file 11 — Reporting Summary [file 41467_2021_22649_MOESM11_ESM.pdf]

nature research

Corresponding author(s): JVG

Last updated by author(s): Mar 3, 2021

Reporting Summary

Nature Research wishes to improve the reproducibility of the work that we publish. This form provides structure for consistency and transparency in reporting. For further information on Nature Research policies, see our Editorial Policies and the Editorial Policy Checklist.

Statistics

For all statistical analyses, confirm that the following items are present in the figure legend, table legend, main text, or Methods section.

n/a	Confirmed	
☐	☒	The exact sample size (*n*) for each experimental group/condition, given as a discrete number and unit of measurement
☒	☐	A statement on whether measurements were taken from distinct samples or whether the same sample was measured repeatedly
☐	☒	The statistical test(s) used AND whether they are one- or two-sided<br>*Only common tests should be described solely by name; describe more complex techniques in the Methods section.*
☒	☐	A description of all covariates tested
☒	☐	A description of any assumptions or corrections, such as tests of normality and adjustment for multiple comparisons
☐	☒	A full description of the statistical parameters including central tendency (e.g. means) or other basic estimates (e.g. regression coefficient) AND variation (e.g. standard deviation) or associated estimates of uncertainty (e.g. confidence intervals)
☐	☒	For null hypothesis testing, the test statistic (e.g. F, t, r) with confidence intervals, effect sizes, degrees of freedom and P value noted<br>*Give P values as exact values whenever suitable.*
☒	☐	For Bayesian analysis, information on the choice of priors and Markov chain Monte Carlo settings
☒	☐	For hierarchical and complex designs, identification of the appropriate level for tests and full reporting of outcomes
☒	☐	Estimates of effect sizes (e.g. Cohen's *d*, Pearson's *r*), indicating how they were calculated

Our web collection on statistics for biologists contains articles on many of the points above.

Software and code

Policy information about availability of computer code

Data collection	Microscopy data were acquired using standard workspace of Olympus IX83 inverted microscopes (CellSens software); Flow cytometry data were collected with Beckman-Coulter Gallios
Data analysis	Image data was analysed using Matlab R2019a; RT-qPCR results were analyzed using Step One™ V2.3 software

For manuscripts utilizing custom algorithms or software that are central to the research but not yet described in published literature, software must be made available to editors and reviewers. We strongly encourage code deposition in a community repository (e.g. GitHub). See the Nature Research guidelines for submitting code & software for further information.

Data

Policy information about availability of data

All manuscripts must include a data availability statement. This statement should provide the following information, where applicable:

- Accession codes, unique identifiers, or web links for publicly available datasets
- A list of figures that have associated raw data
- A description of any restrictions on data availability

All data generated or analysed during this study are included in this published article (Supplementary Data 1 and Supplementary Information).

Field-specific reporting

Life sciences study design

All studies must disclose on these points even when the disclosure is negative.

Sample size	At least three biological repeats were used for all experiments
Data exclusions	No data were excluded, except for determining the communication range of the PlcR-PapR system, where we ignore signal producing clusters smaller than 3µm in width, because these clusters showed significant self-sensing - as we quantify elsewhere in the manuscript.
Replication	Our data were collected for independent biological replicates, each was taken from a different culture. The experiments were performed in at least two separate days. For microscopy chambers, images were acquired from at least two independent flow channels.
Randomization	No randomization was necessary
Blinding	No blinding was necessary

Reporting for specific materials, systems and methods

We require information from authors about some types of materials, experimental systems and methods used in many studies. Here, indicate whether each material, system or method listed is relevant to your study. If you are not sure if a list item applies to your research, read the appropriate section before selecting a response.

Materials & experimental systems

n/a	Involved in the study	
☒	☐	Antibodies
☒	☐	Eukaryotic cell lines
☒	☐	Palaeontology and archaeology
☒	☐	Animals and other organisms
☒	☐	Human research participants
☒	☐	Clinical data
☒	☐	Dual use research of concern

Methods

n/a	Involved in the study	
☒	☐	ChIP-seq
☐	☒	Flow cytometry
☒	☐	MRI-based neuroimaging

Flow Cytometry

Plots

Confirm that:

☒ The axis labels state the marker and fluorochrome used (e.g. CD4-FITC).

☒ The axis scales are clearly visible. Include numbers along axes only for bottom left plot of group (a 'group' is an analysis of identical markers).

☒ All plots are contour plots with outliers or pseudocolor plots.

☒ A numerical value for number of cells or percentage (with statistics) is provided.

Methodology

Sample preparation	Sample of living bacteria that are growing in SMM were taken and diluted in PBS, and then measured in the flow cytometry
Instrument	Beckman-Coulter Gallios flow-cytometer equipped with four lasers (405 nm, 488 nm co-linear with 561 nm, 638 nm). The emission filters used were: BFP – 450/50, YFP – 525/40, mCherry – 620/30.
Software	Beckman-Coulter Gallios software
Cell population abundance	The cells were not sorted
Gating strategy	Cell population was gated using forward and side scatter parameters and non-cell events were excluded. Gating was calibrated once by eye for a given set of samples. Constitutive fluorescent reporters showed a clear bi-modal distribution. Insensitivity to exact decisions of gate was verified. Then, we used BFP and RFP filters to distinguish between the co-cultured genotypes.

☒ Tick this box to confirm that a figure exemplifying the gating strategy is provided in the Supplementary Information.
